# Enhancing Antileishmanial Activity of Amidoxime-Based Compounds Bearing a 4,5-Dihydrofuran Scaffold: In Vitro Screening Against *Leishmania amazonensis*

**DOI:** 10.3390/molecules29225469

**Published:** 2024-11-20

**Authors:** Fabiana Maia Santos Urbancg Moncorvo, Oscar Leonardo Avendaño Leon, Christophe Curti, Youssef Kabri, Sébastien Redon, Eduardo Caio Torres-Santos, Patrice Vanelle

**Affiliations:** 1 Laboratório de Bioquímica de Tripanosomatídeos, Instituto Oswaldo Cruz—FIOCRUZ, Av. Brasil, 4365, Rio de Janeiro 21040-900, Brazil; fabianamaia.santos@gmail.com; 2 Aix Marseille Univ, CNRS, ICR UMR 7273, Equipe Pharmaco-Chimie Radicalaire, Faculté de Pharmacie, 27 Boulevard Jean Moulin, CS30064, CEDEX 05, 13385 Marseille, France; oscar-leonardo.avendano-leon@etu.univ-amu.fr (O.L.A.L.); christophe.curti@univ-amu.fr (C.C.); youssef.kabri@univ-amu.fr (Y.K.); sebastien.redon@univ-amu.fr (S.R.); 3 Service Central de la Qualité et de l’Information Pharmaceutiques (SCQIP), Pharmacy Department, Assistance Publique—Hôpitaux de Marseille (AP-HM), 147 Bd. Baille, 13006 Marseille, France

**Keywords:** leishmaniasis, pharmacomodulation, 4,5-dihydrofuran, amidoxime, manganese (III) acetate

## Abstract

Leishmaniasis, a protozoan disease affecting humans, exposes significant shortcomings in current treatments. In continuation to our previous findings on amidoxime-based antileishmanial compounds bearing a 4,5-dihydrofuran scaffold, twelve new amidoxime derivatives substituted at position 3 with an amide bearing a nitrogen heterocycle were synthesized. This series was designed to replace the sulfone and aryl group on a previously reported HIT. The synthesis of these compounds involved the following three-step pathway: manganese (III) acetate-based cyclization of a β-ketoester, followed by amidation with LiHMDS and a final reaction with hydroxylamine. Three of them, containing either bromine, chlorine, or methyl substitutions and featuring a pyridine moiety, showed an interesting toxicity–activity relationship in vitro. They exhibited IC_50_ values of 15.0 µM, 16.0 µM, and 17.0 µM against the promastigote form of the parasite and IC_50_ values of 0.5 µM, 0.6 µM, and 0.3 µM against the intracellular amastigote form, respectively. A selectivity index (SI) greater than 300 was established between the cytotoxic concentrations (in murine macrophages) and the effective concentrations (against the intracellular form of *Leishmania amazonensis*). This SI is at least seventy times higher than that observed for Pentamidine and twenty-five times higher than that observed for the reference HIT, as previously reported.

## 1. Introduction

Leishmaniases are vector-borne diseases transmitted among mammalian hosts, including humans, via the bites of infected female phlebotomine sandflies [1]. Leishmaniasis groups diseases caused by heteroxenous protozoa from the genus Leishmania [2,3,4]. There are more than 20 well-recognized Leishmania species known to infect humans, generating three principal clinical manifestations, visceral (VL), cutaneous (CL), and mucocutaneous (ML) forms of the disease, with an important global impact on human health and well-being [5]. According to the World Health Organization (WHO), an estimated 50,000 to 90,000 new cases of VL and more than 1 million new cases of CL occur annually, particularly in tropical and subtropical regions [6]. Pharmacovigilance programs for current antileishmanial medications remain insufficient [7,8]. Thus, the efforts to follow up the safety and efficacy of treatments are hampered, especially those based on parenteral regimes such as pentavalent antimonials, pentamidine, or amphotericin B [9,10]. Furthermore, miltefosine, the only oral drug for leishmaniasis, exhibits limitations including embryo-fetal toxicity [11,12]. These constraints, including the emergence of drug-resistant strains, highlight an urgent need for new and effective antileishmanial drugs adapted to oral administration [13,14].

**Scheme 1:**
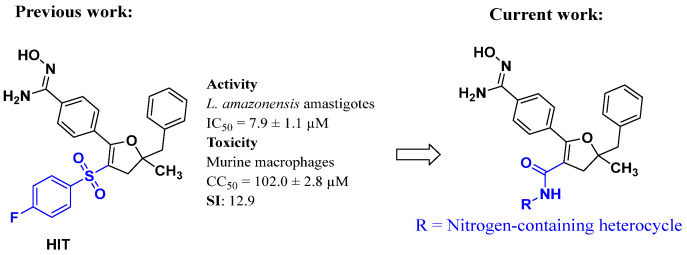
Optimization of 4,5-dihydrofuran derivatives bearing an amidoxime group against *L. amazonensis*.

Our group is interested in the design of compounds with antiparasitic activity against different species of *Leishmania*. We have developed a 4,5-dihydrofuran scaffold which has been previously evaluated against *L. donovani* and *L. amazonensis*. Furthermore, in light of the consensus that has been reached on the criteria for identifying ‘hit’ compounds for infectious diseases [15], a hit compound in this project was defined as one with an activity below 10 µM against the promastigote form of *L. donovani* and *L. amazonensis*. In this regard, a HIT compound was identified, 4-(5-benzyl-3-((4-fluorophenyl)sulfonyl)-5-methyl-4,5-dihydrofuran-2-yl)-*N′*-hydroxybenzimidamide, IC_50_ = 7.9 ± 1.1 µM, for the intracellular amastigote form of *L. amazonensis*, with an SI = 12.9 on murine macrophages [16,17]. *L. donovani* is the causative agent of visceral leishmaniasis, also known as kala-azar, the most severe form of the disease [18]. Additionally, *L. amazonensis* is a prominent cause of cutaneous and diffuse cutaneous leishmaniasis, particularly in South America [19]. The amidoxime moiety in the HIT compound, featuring both oxime and amino functionalities on the same carbon atom (RC(NH_2_)=NOH), proves a pH-responsive behavior that could enhance the potential for drug development [20,21,22], as shown in Scheme 1.

Furthermore, we have previously documented the sensitivity at position 3 of aliphatic derivatives of the 4,5-dihydrofuran scaffold, exhibiting a wide range of activity. Esters, such as ethyl carboxylate derivative, demonstrate better activity (with a promastigote IC_50_ of 6.3 µM) compared to aliphatic amides (with a promastigote IC_50_ > 43 µM) or aliphatic sulphones (with a promastigote IC_50_ > 63 µM) [23].

In the present work, we hypothesized that incorporating a nitrogen-containing heterocycle [24], such as pyridine, in place of an aryl moiety might enhance antileishmanial efficacy [25]. Comparatively, other heterocyclic moieties, like pyrazole [26], pyrazine [27], and pyridazine [28], could offer unique advantages to enhance the target selectivity [29]. Consequently, we report the in vitro biological evaluation of a series of 4,5-dihydrofurane-carboxamide against *Leishmania amazonensis* promastigotes. We also assessed the cytotoxicity and activity against the intracellular amastigote form of the most promising compounds.

## 2. Results and Discussion

### 2.1. Chemistry

**Scheme 2:**
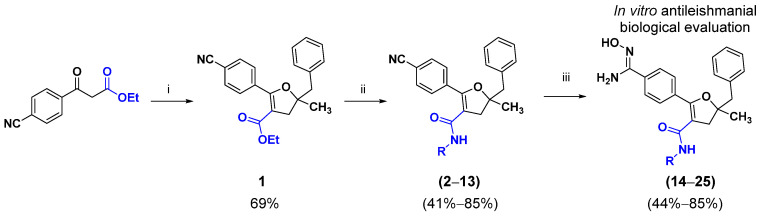
Synthetic pathways of antileishmanial compounds bearing the 4,5-dihydrofuran-3-carboxamide scaffold and an amidoxime moiety. Reagent and conditions: (i) Mn(OAc)_3_ (2.1 equiv.), Cu(OAc)_2_ (1 equiv.), 2-methyl-3-phenyl-1-propene (2 equiv.), AcOH, MW, 80 °C, 100 W, and 3 h. (ii) Amine (1.5 equiv.), LiHMDS (2.5 equiv.), Toluene, 70 °C, 15 h, and N_2_. (iii) KO*t*Bu (10 equiv.), NH_2_OH.HCl (10 equiv.), DMSO, 18 h, 0 °C—RT, and N_2_.

Twelve compounds were synthesized through a three-step process, as shown in Scheme 2. The starting material, ethyl 3-(4-cyanophenyl)-3-oxopropanoate, was cyclized with a terminal alkene, (2-methylallyl)benzene, through an oxidative free-radical reaction mediated by manganese(III) acetate [30]. The 4,5-dihydrofuran-3-carboxylate thus formed, compound **1**, was subjected to a direct amidation reaction mediated by LiHMDS, yielding 4,5-dihydrofuran-3-carboxamide intermediates, compounds **2** to **13**. LiHMDS has been used for its special selectivity to the C-O bond cleavage of unactivated esters [31].

The yield of intermediates **2**–**13** varied between 41% with the phenyl moiety (compound **7**) and 85% with pyridin-2-yl (compound **5**). Subsequently, using a well-documented reaction with hydroxylamine hydrochloride, the nitrile moieties in the 4,5-dihydrofuran-3-carboxamide intermediates were converted into the corresponding amidoxime [32,33], with yields ranging from 44% to 85%, as shown in Table 1.

**Scheme 3:**
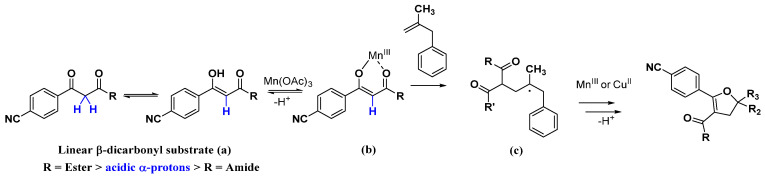
Electron transfer to generate radicals.

The cyclization reaction to access the dihydrofuran moiety was a key step, achieved using a linear β-ketoester rather than a β-ketoamide. Preliminary observations from our team indicated that the reactivity of linear β-ketoamides presents challenges in selectively forming the dihydrofuran-3-carboxamide. Regarding the mechanism of the reaction, the varying capacity to form a Mn(III)-enolate complex affects the reactivity, which is influenced by the acidity of the α-proton(s) in the β-dicarbonyl compounds that affect the rate of enolization, as shown in Scheme 3. For β-ketoester, the enolization of **a** to give **b** is fast and reversible. Moreover, the electron transfer to give the radical **c** is slow. Thus, the rate of β-ketoester cyclization depends on the alkene concentration [34]. Contrarily, for compounds with less acidic α-protons, such as linear β-ketoamides, the formation of the Mn(III)-enolate complex is slower, making this step rate-determining. Consequently, subsequent steps in the reaction could be altered. β-ketoamides can lead to the dihydrofuran product but often result in many byproducts. The linear β-ketoester allowed for the production of dihydrofuran with a good yield (69%).

To guide the selection of synthesized compounds, complementary predicted biopharmaceutic parameters were calculated using open-source software such as Swiss-ADMET^®^ (14.9.29, 2013) [35] and another licensed as ADMET-Predictor^®^ (12.0) [36,37]. To our best knowledge, amide modification could serve as a reasonable first substitution of the sulfone moiety with a view toward further oral use in humans. Most of the amidoxime compounds adhering to Lipinski’s rule of five, except for compound **21**, which bear the benzyl-trifluoromethyl group exhibited higher LogP and molecular weight values, at 5.3 and 507.39 g/mol, respectively. LogP values were calculated between 2.7 and 5.3. Nitrogen-containing modifications provided better preliminary predicted bioavailability than the sulfone-bearing HIT compound. The reference HIT compound showed a bioavailability of 30%, whereas compound **24** exhibited a bioavailability of over 90%, and the remaining compounds suggested around 80%. Moreover, these nitrogen-based modifications enabled the modulation of dissociation properties with the pH variations. The amidoxime moiety is associated with two pKa, a pKa acid around 13 and a pKa basic around 5, depending on the substituents [38]. Predicted values showed that amide substitutions modulate with additional pKa values around 11 and 2, respectively. This pH-dependent dissociation behavior is interesting, first, for a gastrointestinal solubility/absorption balance and, second, regarding a hypothetical mechanism of antileishmanial action based on the pH modulation. The parasitophorous vacuole of macrophages, which contains the amastigote form of the parasite, exhibits an acidic pH near 4.5 [39,40], in contrast to the cytoplasmic pH of 7.2 [41,42]. The promastigote form usually maintains a pH near 6.5 [43,44].

### 2.2. Biology

Initially, compounds **14** to **25** were subjected to preliminary in vitro screening against *Leishmania amazonensis* promastigotes, strain MHOM/BR/77/LTB0016. The 50% growth inhibitory concentration (IC_50_) was measured after 72 h of cultivation with each compound using a resazurin assay conducted in independent triplicate experiments. The results showed that the compounds exhibited antileishmanial activities, with IC_50_ values ranging from 15.0 to 52.5 µM, as presented in Table 2.

Compounds featuring a pyridin-2-yl moiety were the most active in the series against the promastigote form of the parasite. Derivatives **14**, **15**, and **16**, with bromine, chlorine, or methyl substitutions, respectively, exhibited IC_50_ values of 15.0 µM, 16.0 µM, and 17.0 µM. Moreover, the pyridine ring without substitutions showed a marginal decrease in activity, with an IC_50_ of 22.40 µM for compound **17**. Additionally, the pyridin-4-yl derivative, compound **18**, demonstrated similar potency, with an IC_50_ of 23.5 µM, suggesting that the position of one nitrogen heteroatom in the ring does not significantly influence the activity. In contrast, a significant reduction in activity was observed when comparing the pyridine ring to the pyrazine and pyridazine moieties, compound **22** (29.9 µM) and compound **24** (39.4 µM). These differences highlight the impact of having two nitrogen heteroatoms and their relative positioning on the activity. In addition, the derivative bearing a 6-chloropyridazin-3-yl moiety, compound **23**, showed an IC_50_ of 36.4 µM, and compound **25**, which incorporates a 1,5-dimethyl-1*H*-pyrazol-3-yl group, was the least active in the series, with an IC_50_ of 52.5 µM. Additionally, compounds **19** and **20**, featuring a phenyl moiety and used as comparatives lacking the nitrogen heteroatom substitution, exhibited a moderate activity, with IC_50_ values of 23.8 µM. Compound **21**, with the largest lipophilicity due to the trifluoromethyl group, showed an IC_50_ of 26.4 µM. Thus, a clear relationship between the activity and the influence of the electron density on the assessed modifications remains to be elucidated in further largest series. These variations in activity underscore the importance of substituent effects and the ring structure at position 3 in modulating activity against the promastigote form of the parasite.

Additionally, in comparison to our reference compound HIT, bearing a sulfone moiety, the results indicate a reduced activity of the 4-dihydrofurane-3-carboxamide derivatives against the promastigote form of the parasite. However, compounds **14**, **15**, and **16**, identified as the most active in the series, were selected to perform cytotoxicity testing in murine macrophages. The results notably revealed that these compounds do not exhibit significant toxicity. The bromine derivative showed a CC_50_ of 171.5 µM, while the chlorine and methyl derivatives displayed toxicity values > 200 µM.

Thus, an in vitro biological evaluation against the intracellular amastigote form of *Leishmania amazonensis*, which is involved in the human infection, was performed for these three compounds. Interesting antileishmanial activities were obtained, with IC_50_ values of 0.5 µM, 0.6 µM, and 0.3 µM, respectively, showing a very favorable toxicity/activity relationship. The improved activity among these compounds showed no significant differences; therefore, the advantage of the broader selectivity index (SI) is primarily due to the differing cytotoxic behaviors associated with the pyridine ring substitutions. Thus, an SI above 300 was established for compounds **14** and **15**, and above 600 for compound **16**. This SI is at least seventy times higher than that observed for Pentamidine and twenty-five times higher than the one observed for the reference HIT, as previously reported in Table 3.

As a perspective, development efforts will focus on synthesizing a chiral version of the isomer that demonstrates the most promising antileishmanial activity. In vitro tests will be conducted to evaluate the activity of individual enantiomers. Based on these results, enantioselective synthesis methods could be explored.

## 3. Experimental Section

### 3.1. Chemistry

#### 3.1.1. General

Reagents were purchased from Sigma-Aldrich (3050 Spruce Street St. Louis, MO, 63103, USA), Fluorochem (Unit 14 Graphite Way, Hadfield, Glossop SK13 1QH, UK), Fisher Scientific (168 3rd Ave, Waltham, MA 02451, USA), or TCI chemicals (9211 North Harborgate Street, Portland, OR 97203, USA) and used without further purification. Microwave reactions were performed using monomode reactors in a Biotage^®^ Initiator Classic (Uppsala, Sweden) in sealed vials, with output powers ranged up to 400 W.

Reaction monitoring was conducted using thin-layer chromatography on aluminum plates (5 × 5 cm) precoated with silica gel 60 F_254_ nm (Merck-F_254_ or Alugram^®^: Macherey-Nagel) in an appropriate eluent. Visualization was performed using ultraviolet light under a UV lamp (VL-6.CL) at 254 nm (6 W) or 365 nm (6 W).

The LC-MS analysis was conducted on a Thermo Scientific Accela High-Speed LC System^®^ (168 3rd Ave, Waltham, MA, USA) coupled with a single quadrupole mass spectrometer, the Thermo MSQ Plus^®^ (168 3rd Ave, Waltham, MA, USA), with an HPLC column, the Thermo Hypersil Gold^®^ (168 3rd Ave, Waltham, MA 02451, USA) 50 × 2.1 mm (C_18_-bonded), with particles of a diameter of 1.9 mm. The samples were injected into the column at a volume of 1 µL. A total of eight minutes was fixed for the chromatographic analysis, which was conducted using a gradient of the following solvents: methanol/water 50:50 at time zero; then, between zero and four minutes, a linear increase in the proportion of methanol to reach a methanol/water ratio of 95:5. Between four and six minutes, the methanol/water ratio was maintained at 95:5, and between six and seven minutes, a linear decrease in the proportion of methanol to return to a methanol/water ratio of 50:50. The water used was buffered with 5 mM ammonium acetate. The mobile phase had a flow rate of 0.3 mL/min. The LC-MS analysis was performed at the Faculty of Pharmacy in Marseille. The purity of the synthesized compounds was verified by the relative area in the LC chromatogram, with >90% for intermediates and >95% for tested compounds.

The melting points were determined using a Köfler melting point apparatus (Wagner & Munz GmbH, München, Germany) and were not corrected.

The following adsorbent was utilized for the column chromatography: silica gel 60 (Merck KGaA, Darmstadt, Germany, with a particle size of 0.063–0.200 mm; 70–230 mesh ASTM or Macherey-Nagel GmbH & Co. KG, Düren, Germany, with a particle size of 0.063–0.04 mm).

Flash chromatography was conducted on a PuriFlash^®^ 5.020 apparatus (Interchim, Montluçon, France) using a solid deposit in dry-load PF-DEL-F004, which contained Celite^®^ 545 CAS 68855-54-9 (Celite Corp., Lompoc, CA, USA). The maximum pressure was set at 15 bar and the flow rate was 15 mL/min. The ultraviolet–visible detector was configured at a wavelength of 254 nm.

The NMR spectra were recorded at the Faculty of Pharmacy in Marseille using a Bruker Avance NEO 400 MHz NanoBay spectrometer (Bruker, Billerica, MA, USA). Residual ^1^H and ^13^C signals in deuterated solvent (CDCl_3_) were used to calibrate the chemical shifts, obviating the need for an additional internal standard. The ^1^H NMR reference values were CDCl_3_ δ = 7.26 ppm and DMSO-*d*_6_ δ = 2.50 ppm. The ^13^C NMR reference values were CDCl_3_ δ = 77.16 ppm and DMSO-*d*_6_ δ = 39.52 ppm. The data for the ^1^H NMR are reported as follows: chemical shifts (δ) in parts per million (ppm), multiplicity (described as follows: s, singlet; bs, broad singlet; d, doublet; t, triplet; q, quadruplet; dd, doublet of doublet; ddd, doublet of doublet of doublet; m, multiplet), coupling constants (*J*) in Hertz (Hz), and integration. The data for the ^13^C NMR are reported as follows: chemical shifts (δ) in parts per million (ppm).

See Supplementary Material for NMR spectra.

#### 3.1.2. Procedure for Preparation of Ethyl 5-Benzyl-2-(4-cyanophenyl)-5-methyl-4,5-dihydrofuran-3-carboxylate (**1**)

In a 20 mL microwave vial equipped with a stirring bar, a solution of manganese (III) acetate dihydrate (2.1 equiv.) and copper(II) acetate (1 equiv.) in 10 mL of glacial acetic acid was heated at 80 °C under microwave irradiation at 100 W for 15 min. Subsequently, the reaction mixture was cooled, and a solution of ethyl 3-(4-cyanophenyl)-3-oxopropanoate (500 mg, 2.3 mmol, and 1 equiv.) and 2-methyl-3-phenyl-1-propene (608 mg, 4.6 mmol, and 2 equiv.) in 7 mL of acetic acid was added. The reaction mixture was monitored by thin-layer chromatography using cyclohexane/AcOEt (70/30), with an Rf value of 0.69. The reaction mixture was heated for 3 h under microwave irradiation using the same conditions. The resulting product was transferred to 50 mL of cold water and extracted with dichloromethane (3 × 40 mL). The organic extracts were subsequently collected and washed with saturated aqueous NaHCO_3_ (3 × 40 mL) and then dried over Na_2_SO_4_. The filtrate solvent was evaporated under reduced pressure. The crude product was purified by flash chromatography using a PuriFlash^®^ IR-20SI-F0025 column and cyclohexane/AcOEt as eluent, employing a gradient from 100/0 to 80/20 over 20 column volumes. Furthermore, the UV–VIS detector was additionally configured at 307 nm, 235, nm, and 205 nm. The product was obtained in the form of an oily white solid. Yield: 69%. The analytical data are consistent with those in the previous literature [23]. ^1^H NMR (400 MHz, CDCl_3_): δ (ppm) 7.85 (d, ^3^*J*_H-H_ = 8.6 Hz, 2H, 2CH_Ar_), 7.64 (d, ^3^*J*_H-H_ = 8.6 Hz, 2H, 2CH_Ar_), 7.30–7.19 (m, 5H, 5CH_Ar_), 4.08 (q, ^3^*J*_H-H_ = 7.1 Hz, 2H, CH_2_), 3.10 (d, ^2^*J*_H-H_ = 15.5 Hz, 1H, H-(CH_2_)), 3.02 (d, ^2^*J*_H-H_ = 15.3 Hz, 1H, H-(CH_2_)), 2.98 (d, ^2^*J*_H-H_ = 15.3 Hz, 1H, H-(CH_2_)), 2.82 (d, ^2^*J*_H-H_ = 15.5 Hz, 1H, H-(CH_2_)), 1.45 (s, 3H, CH_3_), and 1.17 (t, ^3^*J*_H-H_ = 7.1 Hz, 3H, CH_3_). ^13^C NMR (100 MHz, CDCl_3_): δ (ppm) 164.6 (C), 161.0 (C), 136.1 (C), 134.5 (C), 131.1 (2CH_Ar_), 130.2 (2CH_Ar_), 129.8 (2CH_Ar_), 128.0 (2CH_Ar_), 126.6 (CH_Ar_), 118.3 (C), 113.2 (C), 104.2 (C), 87.8 (C), 59.7 (CH_2_), 46.5 (CH_2_), 42.1 (CH_2_), 26.4 (CH_3_), and 14.0 (CH_3_). HRMS (ESI +) *m*/*z* calcd for C_22_H_21_NO_3_ [M + Na]^+^ 370.1414; found 370.1413.

#### 3.1.3. Procedure for Preparation of Compounds **2** to **13** by Amidation Reaction

A single-neck round-bottom flask of 5 mL equipped with a stirring bar was charged with the intermediate product, compound **1** (1.0 equiv.), the respective amine (1.5 equiv.), and then placed under nitrogen. The vial was subjected to three evacuation/backfilling cycles. Toluene (2 mL) and lithium bis(trimethylsilyl)amide (LiHMDS, 1.0 M in THF, and 2.5 equiv.) were added sequentially with vigorous stirring at room temperature. The reaction mixture was then stirred for 15 h at 70 °C. Subsequently, the reaction mixture was quenched with NH_4_Cl (1.0 M and 5 mL) and extracted with EtOAc (3 × 15 mL). The organic layers were combined, washed with water (1 × 20 mL) and brine (1 × 20 mL), dried over Na_2_SO_4_, and concentrated. Purification by flash chromatography using an appropriate solvent afforded the title product.

*5-Benzyl-N-(5-bromopyridin-2-yl)-2-(4-cyanophenyl)-5-methyl-4,5-dihydrofuran-3-carboxamide* (**2**).

The compound **2** was synthesized following the previously described procedure from 5-bromopyridin-2-amine (112 mg, 0.65 mmol, and 1.5 equiv.). The reaction progress was monitored by TLC (eluent: AcOEt/cyclohexane 40/60; Rf of 0.33). The crude product was purified by flash chromatography using a PuriFlash^®^ IR-50SI-F0025 column and eluted with cyclohexane/AcOEt in a gradient from 100/0 to 80/20 over 25 column volumes. Yield: 45%.

The product was obtained as a light-brown oil. ^1^H NMR (400 MHz, CDCl_3_): δ (ppm) 8.24 (d, ^4^*J*_H-H_ = 2.4 Hz, 1H, CH_Ar_), 8.12 (d, ^3^*J*_H-H_ = 8.9 Hz, 1H, CH_Ar_), 7.81 (d, ^3^*J*_H-H_ = 8.7 Hz, 2H, 2CH_Ar_), 7.75 (dd, ^4^*J*_H-H_ = 2.4 Hz, ^3^*J*_H-H_ = 8.9 Hz, 1H, CH_Ar_), 7.68 (d, ^3^*J*_H-H_ = 8.7 Hz, 2H, 2CH_Ar_), 7.67 (br s, 1H, NH), 7.32–7.22 (m, 5H, 5CH_Ar_), 3.23 (d, ^2^*J*_H-H_ = 14.3 Hz, 1H, H-(CH_2_), 3.08 (d, ^2^*J*_H-H_ = 13.8 Hz, 1H, H-(CH_2_)), 3.04 (d, ^2^*J*_H-H_ = 13.8 Hz, 1H, H-(CH_2_)), 2.92 (d, ^2^*J*_H-H_ = 14.3 Hz, 1H, H-(CH_2_), and 1.54 (s, 3H, CH_3_). ^13^C NMR (100 MHz, CDCl_3_): δ (ppm) 162.7 (C), 160.9 (C), 150.1 (C), 148.3 (CH_Ar_), 141.1 (CH_Ar_), 136.0 (C), 134.5 (C), 131.8 (2CH_Ar_), 130.5 (2CH_Ar_), 129.9 (2CH_Ar_), 128.5 (2CH_Ar_), 127.2 (CH_Ar_), 118.5 (C), 115.5 (CH_Ar_), 114.5 (C), 113.9 (C), 105.9 (C), 88.3 (C), 46.8 (CH_2_), 42.1 (CH_2_), and 27.0 (CH_3_). LC/MS ESI+ tr, 6.59 min, (*m*/*z*) calcd for C_25_H_20_BrN_3_O_2_ [M + H]^+^: found 474.96. HRMS (ESI +) *m*/*z* calcd for C_25_H_20_BrN_3_O_2_ [M + H]^+^ 476.0795; found 476.0798.

*5-Benzyl-N-(5-chloropyridin-2-yl)-2-(4-cyanophenyl)-5-methyl-4,5-dihydrofuran-3-carboxamide* (**3**).

The compound **3** was synthesized following the previously described procedure from 5-chloropyridin-2-amine (112 mg, 0.86 mmol, and 1.5 equiv.). The reaction progress was monitored by TLC (eluent: cyclohexane/AcOEt/dichloromethane 30/40/30; Rf of 0.51). The crude product was purified by flash chromatography using a PuriFlash^®^ IR-50SI-F0025 column and eluted with cyclohexane/AcOEt in a gradient from 100/0 to 70/30 over 25 column volumes. Yield: 48%.

The product was obtained as a light-yellow oil. ^1^H NMR (400 MHz, CDCl_3_): δ (ppm) 8.18 (d, ^3^*J*_H-H_ = 8.9 Hz, 1H, CH_Ar_), 8.14 (d, ^4^*J*_H-H_ = 2.4 Hz, 1H, CH_Ar_), 7.81 (d, ^3^*J*_H-H_ = 8.5 Hz, 2H, 2CH_Ar_), 7.73 (br s, 1H, NH), 7.68 (d, ^3^*J*_H-H_ = 8.5 Hz, 2H, 2CH_Ar_), 7.62 (dd, ^4^*J*_H-H_ = 2.4 Hz, ^3^*J*_H-H_ = 8.9 Hz, 1H, CH_Ar_), 7.33–7.23 (m, 5H, 5CH_Ar_), 3.23 (d, ^2^*J*_H-H_ = 14.2 Hz, 1H, H-(CH_2_), 3.08 (d, ^2^*J*_H-H_ = 13.8 Hz, 1H, H-(CH_2_)), 3.04 (d, ^2^*J*_H-H_ = 13.8 Hz, 1H, H-(CH_2_)), 2.93 (d, ^2^*J*_H-H_ = 14.2 Hz, 1H, H-(CH_2_), and 1.54 (s, 3H, CH_3_). ^13^C NMR (100 MHz, CDCl_3_): δ (ppm) 162.7 (C), 160.9 (C), 149.7 (C), 146.0 (CH_Ar_), 138.4 (CH_Ar_), 136.0 (C), 134.5 (C), 131.8 (2CH_Ar_), 130.5 (2CH_Ar_), 129.9 (2CH_Ar_), 128.5 (2CH_Ar_), 127.2 (CH_Ar_), 126.7 (C), 118.5 (C), 115.0 (CH_Ar_), 113.9 (C), 105.9 (C), 88.3 (C), 46.8 (CH_2_), 42.1 (CH_2_), and 27.0 (CH_3_). LC/MS ESI+ tr, 6.54 min, (*m*/*z*) calcd for C_25_H_20_ClN_3_O_2_ [M + H]^+^: found 430.06. HRMS (ESI+) *m*/*z* calcd for C_25_H_20_ClN_3_O_2_ [M + H]^+^ 430.1317; found 413.1314.

*5-Benzyl-2-(4-cyanophenyl)-5-methyl-N-(5-methylpyridin-2-yl)-4,5-dihydrofuran-3-carboxamide* (**4**).

The compound **4** was synthesized following the previously described procedure from 5-methylpyridin-2-amine (74 mg, 0.68 mmol, and 1.5 equiv.). The reaction progress was monitored by TLC (eluent: cyclohexane/AcOEt/dichloromethane 40/30/30; Rf of 0.55). The crude product was purified by flash chromatography using a PuriFlash^®^ IR-50SI-F0025 column and eluted with cyclohexane/AcOEt in a gradient from 100/0 to 70/30 over 25 column volumes. Yield: 66%. The product was obtained as a light-yellow oil. ^1^H NMR (400 MHz, CDCl_3_): δ (ppm) 8.13 (d, ^3^*J*_H-H_ = 8.6 Hz, 1H, CH_Ar_), 8.01 (m, 2H, NH, CH_Ar_), 7.82 (d, ^3^*J*_H-H_ = 8.4 Hz, 2H, 2CH_Ar_), 7.67 (d, ^3^*J*_H-H_ = 8.4 Hz, 2H, 2CH_Ar_), 7.53 (dd, ^4^*J*_H-H_ = 2.0 Hz, ^3^*J*_H-H_ = 8.6 Hz, 1H, CH_Ar_), 7.34–7.20 (m, 5H, 5CH_Ar_), 3.27 (d, ^2^*J*_H-H_ = 14.4 Hz, 1H, H-(CH_2_), 3.08 (d, ^2^*J*_H-H_ = 13.8 Hz, 1H, H-(CH_2_)), 3.04 (d, ^2^*J*_H-H_ = 13.8 Hz, 1H, H-(CH_2_), 2.96 (d, ^2^*J*_H-H_ = 14.4 Hz, 1H, H-(CH_2_)), 2.28 (s, 3H, CH_3_), and 1.53 (s, 3H, CH_3_). ^13^C NMR (100 MHz, CDCl_3_): δ (ppm) 162.8 (C), 160.9 (C), 149.0 (C), 145.5 (CH_Ar_), 140.5 (CH_Ar_), 136.1 (C), 134.7 (C), 131.8 (2CH_Ar_), 130.5 (2CH_Ar_), 130.0 (2CH_Ar_), 129.3 (C), 128.5 (2CH_Ar_), 127.2 (CH_Ar_), 118.6 (C), 114.4 (CH_Ar_), 113.8 (C), 106.1 (C), 88.3 (C), 46.8 (CH_2_), 42.3 (CH_2_), 27.0 (CH_3_), and 17.9 (CH_3_). LC/MS ESI+ tr, 6.10 min, (*m*/*z*) calcd for C_26_H_23_N_3_O_2_ [M + H]^+^: found 410.11. HRMS (ESI+) *m*/*z* calcd for C_26_H_23_N_3_O_2_ [M + H]^+^ 410.1863; found 410.1870.

*5-Benzyl-2-(4-cyanophenyl)-5-methyl-N-(pyridin-2-yl)-4,5-dihydrofuran-3-carboxamide* (**5**).

The compound **5** was synthesized following the previously described procedure from pyridin-2-amine (41 mg, 0.43 mmol, and 1.5 equiv.). The reaction progress was monitored by TLC (eluent: cyclohexane/AcOEt/dichloromethane 30/40/30; Rf of 0.80). The crude product was purified by flash chromatography using a PuriFlash^®^ IR-50SI-F0025 column and eluted with cyclohexane/AcOEt in a gradient from 100/0 to 70/30 over 25 column volumes. A UV–VIS detector was configurated additionally at 222 nm and 230 nm. Yield: 85%. The product was obtained as a light-brown oil. ^1^H NMR (400 MHz, CDCl_3_): δ (ppm) 8.24–8.17 (m, 2H, 2CH_Ar_), 7.89 (br s, 1H, NH), 7.83 (d, ^3^*J*_H-H_ = 8.6 Hz, 2H, 2CH_Ar_), 7.70–7.67 (m, 3H, 3CH_Ar_), 7.33–7.23 (m, 5H, 5CH_Ar_), 7.05–7.01 (m, 1H, CH_Ar_), 3.26 (d, ^2^*J*_H-H_ = 14.4 Hz, 1H, H-(CH_2_)), 3.09 (d, ^2^*J*_H-H_ = 13.8 Hz, 1H, H-(CH_2_)), 3.05 (d, ^2^*J*_H-H_ = 13.8 Hz, 1H, H-(CH_2_)), 2.96 (d, ^2^*J*_H-H_ = 14.4 Hz, 1H, H-(CH_2_)), and 1.54 (s, 3H, CH_3_). ^13^C NMR (100 MHz, CDCl_3_): δ (ppm) 162.9 (C), 160.7 (C), 151.3 (C), 146.9 (CH_Ar_), 139.1 (CH_Ar_), 136.1 (C), 134.7 (C), 131.8 (2CH_Ar_), 130.5 (2CH_Ar_), 130.0 (2CH_Ar_), 128.5 (2CH_Ar_), 127.2 (CH_Ar_), 119.7 (CH_Ar_), 118.6 (C), 114.5 (CH_Ar_), 113.8 (C), 106.2 (C), 88.2 (C), 46.8 (CH_2_), 42.3 (CH_2_), and 27.0 (CH_3_). Analysis calculated for C_25_H_21_N_3_O_2_: HRMS: *m*/*z* [M + H]^+^ calculated 396.1707; found 396.1703.

*5-Benzyl-2-(4-cyanophenyl)-5-methyl-N-(pyridin-4-yl)-4,5-dihydrofuran-3-carboxamide* (**6**).

The compound **6** was synthesized following the previously described procedure from and pyridin-4-amine (40 mg, 0.43 mmol, and 1.5 equiv.). The reaction progress was monitored by TLC (eluent: dichloromethane/methanol 96/4; Rf of 0.38). The crude product was purified by flash chromatography using a PuriFlash^®^ IR-50SI-F0025 column and eluted with dichloromethane/methanol in a gradient from 100/0 to 95/5 over 25 column volumes. A UV–VIS detector was configurated additionally at 222 nm and 230 nm. Yield: 50%. The product was obtained as a yellow oil. ^1^H NMR (400 MHz, CDCl_3_): δ (ppm) 8.35 (d, ^3^*J*_H-H_ = 6.5 Hz, 2H, 2CH_Ar_), 7.78 (d, ^3^*J*_H-H_ = 8.6 Hz, 2H, 2CH_Ar_), 7.68 (d, ^3^*J*_H-H_ = 8.6 Hz, 2H, 2CH_Ar_), 7.44 (d, ^3^*J*_H-H_ = 6.5 Hz, 2H, 2CH_Ar_), 7.39 (br s, 1H, NH), 7.32–7.23 (m, 5H, 5CH_Ar_), 3.24 (d, ^2^*J*_H-H_ = 14.5 Hz, 1H, H-(CH_2_), 3.07 (d, ^2^*J*_H-H_ = 13.9 Hz, 1H, H-(CH_2_), 3.04 (d, ^2^*J*_H-H_ = 13.9 Hz, 1H, H-(CH_2_), 2.98 (d, ^2^*J*_H-H_ = 14.5 Hz, 1H, H-(CH_2_)), and 1.55 (s, 3H, CH_3_). ^13^C NMR (100 MHz, CDCl_3_): δ (ppm) 163.2 (C), 159.9 (C), 150.5 (2CH_Ar_), 145.1 (C), 136.1 (C), 134.4 (C), 132.0 (2CH_Ar_), 130.5 (2CH_Ar_), 129.8 (2CH_Ar_), 128.5 (2CH_Ar_), 127.2 (CH_Ar_), 118.4 (C), 114.1 (C), 113.5 (2CH_Ar_), 106.5 (C), 88.3 (C), 46.9 (CH_2_), 42.3 (CH_2_), and 27.1 (CH_3_). Analysis calculated for C_25_H_21_N_3_O_2_: HRMS: *m*/*z* [M + H]^+^ calculated 396.1707; found 396.1702.

*5-Benzyl-2-(4-cyanophenyl)-5-methyl-N-phenyl-4,5-dihydrofuran-3-carboxamide* (**7**).

The compound **7** was synthesized following the previously described procedure from aniline (40 mg, 0.43 mmol, and 1.5 equiv.). The reaction progress was monitored by TLC (eluent: cyclohexane/AcOEt/dichloromethane 3/3/4; Rf of 0.92). The crude product was purified by flash chromatography using a PuriFlash^®^ IR-50SI-F0025 column and eluted with cyclohexane/AcOEt in a gradient from 100/0 to 70/30 over 25 column volumes. A UV–VIS detector was configured at 242 nm, 290 nm and 327 nm. Yield: 41%. The product was obtained as an oily yellow solid. ^1^H NMR (400 MHz, CDCl_3_): δ (ppm) 7.81 (d, ^3^*J*_H-H_ = 8.4 Hz, 2H, 2CH_Ar_), 7.67 (d, ^3^*J*_H-H_ = 8.4 Hz, 2H, 2CH_Ar_), 7.52–7.43 (m, 1H, CH_Ar_), 7.34–7.27 (m, 8H, 8CH_Ar_), 7.08 (t, ^3^*J*_H-H_ = 7.2 Hz, 1H, CH_Ar_), 6.70 (br s, 1H, NH), 3.19 (d, ^2^*J*_H-H_ = 14.7 Hz, 1H, H-(CH_2_), 3.08 (d, ^2^*J*_H-H_ = 13.8 Hz, 1H, H-(CH_2_), 3.03 (d, ^2^*J*_H-H_ = 13.8 Hz, 1H, H-(CH_2_), 2.95 (d, ^2^*J*_H-H_ = 14.7 Hz, 1H, H-(CH_2_), and 1.55 (s, 3H, CH_3_). ^13^C NMR (100 MHz, CDCl_3_): δ (ppm) 169.2 (C), 157.9 (C), 137.8 (C), 136.4 (C), 134.6 (C), 132.0 (2CH_Ar_), 130.5 (2CH_Ar_), 129.7 (2CH_Ar_), 129.2 (2CH_Ar_), 128.5 (2CH_Ar_), 127.1 (CH_Ar_), 124.5 (CH_Ar_), 120.0 (2CH_Ar_), 118.5 (C), 113.7 (C), 107.3 (C), 87.6 (C), 47.0 (CH_2_), 42.8 (CH_2_), and 27.1 (CH_3_). Analysis calculated for C_26_H_22_N_2_O_2_: HRMS: *m*/*z* [M + H]^+^ calculated 395.1754; found 395.1753.

*5-Benzyl-2-(4-cyanophenyl)-N-(4-fluorophenyl)-5-methyl-4,5-dihydrofuran-3-carboxamide* (**8**).

The compound **8** was synthesized following the previously described procedure from 4-fluoroaniline (45 mg, 0.40 mmol, and 1.5 equiv.). The reaction progress was monitored by TLC (eluent: cyclohexane/AcOEt 50/00; Rf of 0.29). The crude product was purified by flash chromatography using a PuriFlash^®^ IR-50SI-F0025 column and eluted with cyclohexane/ethyl acetate in a gradient from 100/0 to 60/40 over 25 column volumes. A UV–VIS detector configured additionally at 225 nm and 230 nm. Yield: 68%. The product was obtained as a light-brown oil. ^1^H NMR (400 MHz, CDCl_3_): δ (ppm) 7.81 (d, ^3^*J*_H-H_ = 8.6 Hz, 2H, 2CH_Ar_), 7.67 (d, ^3^*J*_H-H_ = 8.6 Hz, 2H, 2CH_Ar_), 7.34–7.25 (m, 7H, 7CH_Ar_), 7.02–6.95 (m, 2H, 2CH_Ar_), 6.66 (br s, 1H, NH), 3.18 (d, ^2^*J*_H-H_ = 14.5 Hz, 1H, H-(CH_2_), 3.08 (d, ^2^*J*_H-H_ = 13.7 Hz, 1H, H-(CH_2_), 3.03 (d, ^2^*J*_H-H_ = 13.7 Hz, 1H, H-(CH_2_), 2.93 (d, ^2^*J*_H-H_ = 14.5 Hz, 1H, H-(CH_2_), and 1.55 (s, 3H, CH_3_). ^13^C NMR (100 MHz, CDCl_3_): δ (ppm) 162.9 (C), 159.5 (d, *J_C-F_* = 246.3 Hz, C), 158.3 (C), 136.8 (C), 136.3 (C), 133.7 (d, *J_C-F_* = 3.1 Hz, C), 130.5 (2CH_Ar_), 129.8 (2CH_Ar_), 128.5 (2CH_Ar_), 127.3 (2CH_Ar_), 127.2 (CH_Ar_), 121.1 (d, *J_C-F_* = 8.6 Hz, 2CH_Ar_), 118.5 (C), 115.9 (d, *J_C-F_* = 21.6 Hz, 2CH_Ar_), 113.7 (C), 106.9 (C), 87.6 (C), 47.0 (CH_2_), 42.8 (CH_2_), and 27.1 (CH_3_). ^19^F NMR (376.5 MHz, CDCl_3_): δ (ppm) −103.3. LC/MS ESI+ tr, 5.10 min, (*m*/*z*) calcd for C_26_H_21_FN_2_O_2_ [M + H]^+^: found 413.06. HRMS (ESI +) *m*/*z* calcd for C_26_H_21_FN_2_O_2_ [M + H]^+^ 413.1660; found 413.1662.

*5-Benzyl-2-(4-cyanophenyl)-5-methyl-N-(4-(trifluoromethyl)benzyl)-4,5-dihydrofuran-3-carboxamide* (**9**).

The compound **9** was synthesized following the previously described procedure from (4-(trifluoromethyl)phenyl)methanamine (76 mg, 0.4 mmol, and 1.5 equiv.). The reaction progress was monitored by TLC (eluent: cyclohexane/AcOEt 70/30; Rf of 0.19). The crude product was purified by flash chromatography using a PuriFlash^®^ IR-50SI-F0025 column and eluted with cyclohexane/ethyl acetate in a gradient from 100/0 to 70/30 over 25 column volumes. A UV–VIS detector was configured additionally in 225 nm and 230 nm. Yield: 47%. The product was obtained as a light-brown oil. ^1^H NMR (400 MHz, CDCl_3_): δ (ppm) 7.81 (d, ^3^*J*_H-H_ = 8.4 Hz, 2H, 2CH_Ar_), 7.62 (d, ^3^*J*_H-H_ = 8.4 Hz, 2H, 2CH_Ar_), 7.58 (d, ^3^*J*_H-H_ = 8.1 Hz, 2H, 2CH_Ar_), 7.32 (d, ^3^*J*_H-H_ = 8.1 Hz, 2H, 2CH_Ar_), 7.27–7.22 (m, 5H, 5CH_Ar_), 5.44 (br s, 1H, NH), 4.50–4.42 (m, 2H, CH_2_), 3.07 (d, ^2^*J*_H-H_ = 14.5 Hz, 1H, H-(CH_2_)), 3.04 (d, ^2^*J*_H-H_ = 13.8 Hz, 1H, H-(CH_2_)), 2.99 (d, ^2^*J*_H-H_ = 13.8 Hz, 1H, H-(CH_2_)), 2.82 (d, ^2^*J*_H-H_ = 14.5 Hz, 1H, H-(CH_2_)), and 1.51 (s, 3H, CH_3_). ^13^C NMR (100 MHz, CDCl_3_): δ (ppm) 164.7 (C), 158.0 (C), 142.4 (C), 136.3 (C), 134.7 (C), 131.7 (2CH_Ar_), 130.5 (2CH_Ar_), 130.2 (q, ^2^*J_C-F_* = 34.6 Hz, C), 129.8 (2CH_Ar_), 128.4 (2CH_Ar_), 128.1 (2CH_Ar_), 127.1 (CH_Ar_), 125.8 (q, ^3^*J_C-F_* = 3.7 Hz, 2CH_Ar_), 124.2 (q, ^1^*J_C-F_* = 271.8 Hz, CF_3_), 118.6 (C), 113.5 (C), 106.1 (C), 87.4 (C), 47.0 (CH_2_), 43.0 (CH_2_), 42.7 (CH_2_), and 27.1 (CH_3_). ^19^F NMR (376.5 MHz, CDCl_3_): δ (ppm) −62.5. Analysis calculated for C_28_H_23_F_3_N_2_O_2_: HRMS: *m*/*z* [M + H]^+^ calculated 477.1784; found 477.1786.

*5-Benzyl-2-(4-cyanophenyl)-5-methyl-N-(pyridazin-3-yl)-4,5-dihydrofuran-3-carboxamide* (**10**).

The compound **10** was synthesized following the previously described procedure from pyridazin-3-amine (41 mg, 0.43 mmol, and 1.5 equiv.). The reaction progress was monitored by TLC (eluent: cyclohexane/AcOEt/dichloromethane 2/6/2; Rf of 0.35). The crude product was purified by flash chromatography using a PuriFlash^®^ IR-50SI-F0025 column and eluted with cyclohexane/ethyl acetate in a gradient from 100/0 to 60/40 over 25 column volumes. A UV–VIS detector was configured to 241 nm, 212 nm, and 315 nm. Yield: 43%. The product was obtained as a yellow oil. ^1^H NMR (400 MHz, CDCl_3_): δ (ppm) 8.84 (d, ^3^*J*_H-H_ = 12.1 Hz, 1H, CH_Ar_), 8.68 (br s, 1H, NH), 8.50 (d, ^3^*J*_H-H_ = 8.7 Hz, 1H, CH_Ar_), 7.81 (d, ^3^*J*_H-H_ = 8.4 Hz, 2H, 2CH_Ar_), 7.68 (d, ^3^*J*_H-H_ = 8.4 Hz, 2H, 2CH_Ar_), 7.61 (dd, ^3^*J*_H-H_ = 12.1 Hz, ^3^*J*_H-H_ = 8.7 Hz, 1H, CH_Ar_), 7.35–7.20 (m, 5H, 5CH_Ar_), 3.32 (d, ^2^*J*_H-H_ = 14.3 Hz, 1H, H-(CH_2_)), 3.10 (d, ^2^*J*_H-H_ = 13.8 Hz, 1H, H-(CH_2_)), 3.06 (d, ^2^*J*_H-H_ = 13.8 Hz, 1H, H-(CH_2_)), 3.02 (d, ^2^*J*_H-H_ = 14.3 Hz, 1H, H-(CH_2_)), and 1.56 (s, 3H, CH_3_). ^13^C NMR (100 MHz, CDCl_3_): δ (ppm) 163.6 (C), 162.1 (C), 155.3 (C), 152.0 (C), 148.2 (CH_Ar_), 135.9 (C), 134.5 (C), 131.8 (2CH_Ar_), 130.5 (2CH_Ar_), 130.0 (2CH_Ar_), 128.5 (2CH_Ar_), 128.1 (CH_Ar_), 127.2 (CH_Ar_), 119.7 (CH_Ar_), 118.4 (C), 105.7 (C), 88.7 (C), 46.8 (CH_2_), 41.9 (CH_2_), and 27.0 (CH_3_). Analysis calculated for C_24_H_20_N_4_O_2_: HRMS: *m*/*z* [M + H]^+^ calculated 397.1659; found 397.1657.

*5-Benzyl-N-(6-chloropyridazin-3-yl)-2-(4-cyanophenyl)-5-methyl-4,5-dihydrofuran-3-carboxamide* (**11**).

The compound **11** was synthesized following the previously described procedure from 6-chloropyridazin-3-amine (86 mg, 0.67 mmol, and 1.5 equiv.). The reaction progress was monitored by TLC (eluent: cyclohexane/AcOEt/dichloromethane 6/2/2; Rf of 0.41). The crude product was purified by flash chromatography using a PuriFlash^®^ IR-50SI-F0025 column and eluted with cyclohexane/AcOEt in a gradient from 100/0 to 70/30 over 25 column volumes. A UV–VIS detector was configured additionally to 210 nm and 230 nm. Yield: 59%. ^1^H NMR (400 MHz, CDCl_3_): δ (ppm) 8.53 (d, ^3^*J*_H-H_ = 9.5 Hz, 1H, CH_Ar_), 7.80 (d, ^3^*J*_H-H_ = 8.6 Hz, 2H, 2CH_Ar_), 7.69 (d, ^3^*J*_H-H_ = 8.6 Hz, 2H, 2CH_Ar_), 7.48 (d, ^3^*J*_H-H_ = 9.5 Hz, 1H, CH_Ar_), 7.35–7.20 (m, 5H, 5CH_Ar_), 3.31 (d, ^2^*J*_H-H_ = 14.3 Hz, 1H, H-(CH_2_), 3.11 (d, ^2^*J*_H-H_ = 13.9 Hz, 1H, H-(CH_2_), 3.05 (d, ^2^*J*_H-H_ = 13.9 Hz, 1H, H-(CH_2_), 3.01 (d, ^2^*J*_H-H_ = 14.3 Hz, 1H, H-(CH_2_), and 1.58 (s, 3H, CH_3_). NH not observed. ^13^C NMR (100 MHz, CDCl_3_): δ (ppm) 163.3 (C), 154.4 (C), 152.0 (C), 135.9 (2C), 134.4 (C), 131.9 (2CH_Ar_), 130.6 (CH_Ar_), 130.5 (2CH_Ar_), 130.0 (2CH_Ar_), 128.5 (2CH_Ar_), 127.3 (CH_Ar_), 122.0 (CH_Ar_), 118.4 (C), 114.2 (C), 105.3 (C), 89.0 (C), 46.8 (CH_2_), 41.7 (CH_2_), and 27.1 (CH_3_). Analysis calculated for C_24_H_19_ClN_4_O_2_: HRMS: *m*/*z* [M + Na]^+^ calculated 431.1183; found 431.1184.

*5-Benzyl-2-(4-cyanophenyl)-5-methyl-N-(pyrazin-2-yl)-4,5-dihydrofuran-3-carboxamide* (**12**).

The compound **12** was synthesized following the previously described procedure from pyrazin-2-amine (41 mg, 0.43 mmol, and 1.5 equiv.). The reaction progress was monitored by TLC (eluent: cyclohexane/AcOEt/dichloromethane 3/4/3; Rf of 0.57). The crude product was purified by flash chromatography using a PuriFlash^®^ IR-50SI-F0025 column and eluted with cyclohexane/AcOEt in a gradient from 100/0 to 70/30 over 25 column volumes. A UV–VIS detector was configured additionally to 210 nm and 230 nm. Yield: 59%. The product was obtained as light-yellow oil. ^1^H NMR (400 MHz, CDCl_3_): δ (ppm) 9.48 (s, 1H, NH), 8.28 (d, ^3^*J*_H-H_ = 2.4 Hz, 1H, CH_Ar_), 8.17 (d, ^3^*J*_H-H_ = 2.4 Hz, 1H, CH_Ar_), 7.82 (d, ^3^*J*_H-H_ = 8.5 Hz, 2H, 2CH_Ar_), 7.70 (d, ^3^*J*_H-H_ = 8.5 Hz, 2H, 2CH_Ar_), 7.49 (br s, 1H, CH_Ar_), 7.34–7.20 (m, 5H, 5CH_Ar_), 3.24 (d, ^2^*J*_H-H_ = 14.3 Hz, 1H, H-(CH_2_)), 3.10 (d, ^2^*J*_H-H_ = 13.8 Hz, 1H, H-(CH_2_)), 3.04 (d, ^2^*J*_H-H_ = 13.8 Hz, 1H, H-(CH_2_)), 2.95 (d, ^2^*J*_H-H_ = 14.3 Hz, 1H, H-(CH_2_)), and 1.56 (s, 3H, CH_3_). ^13^C NMR (100 MHz, CDCl_3_): δ (ppm) 162.5 (C), 161.2 (C), 148.2 (C), 141.9 (CH_Ar_), 140.0 (CH_Ar_), 137.2 (CH_Ar_), 135.9 (C), 134.4 (C), 132.0 (2CH_Ar_), 130.5 (2CH_Ar_), 130.0 (2CH_Ar_), 128.5 (2CH_Ar_), 127.3 (CH_Ar_), 118.4 (C), 114.2 (C), 105.6 (C), 88.4 (C), 46.8 (CH_2_), 42.1 (CH_2_), and 27.1 (CH_3_). Analysis calculated for C_24_H_20_N_4_O_2_: HRMS: *m*/*z* [M + Na]^+^ calculated 419.1478; found 419.1480.

*5-Benzyl-2-(4-cyanophenyl)-N-(1,5-dimethyl-1H-pyrazol-3-yl)-5-methyl-4,5-dihydrofuran-3-carboxamide* (**13**).

The compound **13** was synthesized following the previously described procedure from 1,5-dimethyl-1*H*-pyrazol-3-amine (48 mg, 0.43 mmol, and 1.5 equiv.). The reaction progress was monitored by TLC (eluent: cyclohexane/AcOEt/dichloromethane 3/4/3; Rf of 0.29). The crude product was purified by flash chromatography using a PuriFlash^®^ IR-50SI-F0025 column and eluted with cyclohexane/ethyl acetate in a gradient from 100/0 to 70/30 over 25 column volumes. A UV–VIS detector was configured to 238 nm and 320 nm. Yield: 66%. The product was obtained as a light-brown oil. ^1^H NMR (400 MHz, CDCl_3_): δ (ppm) 7.84 (d, ^3^*J*_H-H_ = 8.4 Hz, 2H, 2CH_Ar_), 7.64 (d, ^3^*J*_H-H_ = 8.4 Hz, 2H, 2CH_Ar_), 7.60 (br s, 1H, NH), 7.30–7.18 (m, 5H, 5CH_Ar_), 6.43 (s, 1H, CH_Ar_), 3.62 (s, 3H, CH_3_), 3.15 (d, ^2^*J*_H-H_ = 14.4 Hz, 1H, H-(CH_2_), 3.04 (d, ^2^*J*_H-H_ = 14.2 Hz, 1H, H-(CH_2_), 3.00 (d, ^2^*J*_H-H_ = 14.2 Hz, 1H, H-(CH_2_), 2.85 (d, ^2^*J*_H-H_ = 14.4 Hz, 1H, H-(CH_2_), 2.21 (s, 3H, CH_3_), and 1.49 (s, 3H, CH_3_). ^13^C NMR (100 MHz, CDCl_3_) δ (ppm) 161.7 (C), 159.2 (C), 145.3 (C), 139.9 (C), 136.1 (C), 134.7 (C), 131.7 (2CH_Ar_), 130.4 (2CH_Ar_), 129.8 (2CH_Ar_), 128.4 (2CH_Ar_), 127.0 (CH_Ar_), 118.6 (C), 113.4 (C), 106.0 (C), 97.4 (CH_Ar_), 87.6 (C), 46.7 (CH_2_), 42.4 (CH_2_), 35.6 (CH_3_), 26.9 (CH_3_), and 11.4 (CH_3_). Analysis calculated for C_25_H_24_N_4_O_2_: HRMS: *m*/*z* [M + H]^+^ calculated 413.1972; found 413.1972.

#### 3.1.4. General Procedure for Amidoxime Synthesis

Hydroxylamine hydrochloride (10 equiv.) in a solvent mixture of dimethyl sulfoxide was stirred under an inert atmosphere of nitrogen while being cooled to 0 °C. Gradual addition of potassium *tert*-butoxide (10 equiv.) followed, and was subsequently stirred for 30 min. Then, one equivalent of the required nitrile was added, and the reaction mixture was stirred to reach room temperature for 18 h. Upon completion of the reaction, the mixture was poured into cold water and extracted with EtOAc (3 × 15 mL). The combined organic phases were washed sequentially with water (1 × 20 mL) and brine (2 × 20 mL), then dried over Na_2_SO_4_ and concentrated. The final product was obtained through purification via flash or column chromatography, employing an appropriate solvent.

*5-Benzyl-N-(5-bromopyridin-2-yl)-2-(4-(N′-hydroxycarbamimidoyl)phenyl)-5-methyl-4,5-dihydrofuran-3-carboxamide* (**14**).

The compound **14** was synthesized following the previously described procedure from 2 (82 mg, 0.17 mmol, and 1 equiv.). The reaction progress was monitored by TLC (eluent: cyclohexane/AcOEt/dichloromethane 4/3/3; Rf of 0.26). The crude product was purified by flash chromatography using a PuriFlash^®^ IR-50SI-F0025 column and eluted with cyclohexane/AcOEt in a gradient from 100/0 to 70/30 over 25 column volumes. A UV–VIS detector was configured additionally to 222 nm and 230 nm. Yield: 68%. The product was obtained as a light brown oil. ^1^H NMR (400 MHz, CDCl_3_): δ (ppm) 8.21 (d, ^4^*J*_H-H_ = 2.5 Hz, 1H, CH_Ar_), 8.12 (d, ^3^*J*_H-H_ = 8.9 Hz, 1H, CH_Ar_), 7.80 (br s, 1H, NH), 7.72 (dd, ^4^*J*_H-H_ = 2.5 Hz, ^3^*J*_H-H_ = 8.9 Hz, 1H, CH_Ar_), 7.69 (d, ^3^*J*_H-H_ = 8.7 Hz, 2H, 2CH_Ar_), 7.66 (d, ^3^*J*_H-H_ = 8.7 Hz, 2H, 2CH_Ar_), 7.33–7.19 (m, 5H, 5CH_Ar_), 5.10 (br s, 2H, NH_2_), 3.17 (d, ^2^*J*_H-H_ = 14.3 Hz, 1H, H-(CH_2_), 2.99 (s, 2H, CH_2_), 2.87 (d, ^2^*J*_H-H_ = 14.3 Hz, 1H, H-(CH_2_), and 1.46 (s, 3H, CH_3_). OH not observed. ^13^C NMR (100 MHz, CDCl_3_): δ (ppm) 163.4 (C), 161.5 (C), 152.6 (C), 150.4 (C), 148.6 (CH_Ar_), 140.8 (CH_Ar_), 136.3 (C), 133.9 (C), 131.9 (C), 130.5 (2CH_Ar_), 129.6 (2CH_Ar_), 128.4 (2CH_Ar_),127.1 (CH_Ar_), 126.0 (2CH_Ar_), 115.5 (CH_Ar_), 114.2 (C), 105.2 (C), 87.8 (C), 46.8 (CH_2_), 42.3 (CH_2_), and 26.8 (CH_3_). Analysis calculated for C_25_H_23_BrN_4_O_3_: HRMS: *m*/*z* [M + H]^+^ calculated 509.1010; found 509.1010.

*5-Benzyl-N-(5-chloropyridin-2-yl)-2-(4-(N′-hydroxycarbamimidoyl)phenyl)-5-methyl-4,5-dihydrofuran-3-carboxamide* (**15**).

The compound **15** was synthesized following the previously described procedure from 3 (90 mg, 0.21 mmol, and 1 equiv.). The reaction progress was monitored by TLC (eluent: cyclohexane/AcOEt/dichloromethane 4/3/3; Rf of 0.59). The crude product was purified by flash chromatography using a PuriFlash^®^ IR-50SI-F0025 column and eluted with cyclohexane/AcOEt in a gradient from 100/0 to 70/30 over 25 column volumes. A UV–VIS detector was configured additionally to 222 nm and 230 nm. Yield: 55%. The product was obtained as a light-yellow oil. ^1^H NMR (400 MHz, CDCl_3_): δ (ppm) 8.17 (d, ^3^*J*_H-H_ = 8.9 Hz, 1H, CH_Ar_), 8.12 (d, ^4^*J*_H-H_ = 2.6 Hz, 1H, CH_Ar_), 7.88 (br s, 1H, NH), 7.69 (d, ^3^*J*_H-H_ = 8.9 Hz, 2H, 2CH_Ar_), 7.66 (d, ^3^*J*_H-H_ = 8.9 Hz, 2H, 2CH_Ar_), 7.58 (dd, ^4^*J*_H-H_ = 2.6 Hz, ^3^*J*_H-H_ = 8.9 Hz, 1H, CH_Ar_), 7.31–7.18 (m, 5H, 5CH_Ar_), 5.06 (br s, 2H, NH_2_), 3.15 (d, ^2^*J*_H-H_ = 14.4 Hz, 1H, H-(CH_2_), 3.00 (d, ^2^*J*_H-H_ = 14.2 Hz, 1H, H-(CH_2_), 2.95 (d, ^2^*J*_H-H_ = 14.2 Hz, 1H, H-(CH_2_), 2.86 (d, ^2^*J*_H-H_ = 14.4 Hz, 1H, H-(CH_2_), and 1.43 (s, 3H, CH_3_). OH not observed. ^13^C NMR (100 MHz, CDCl_3_): δ (ppm) 163.4 (C), 161.4 (C), 152.5 (C), 150.3 (C), 146.3 (CH_Ar_), 138.1 (CH_Ar_), 136.3 (C), 134.0 (C), 131.8 (C), 130.5 (2CH_Ar_), 129.6 (2CH_Ar_), 128.4 (2CH_Ar_),127.0 (CH_Ar_), 126.4 (C), 125.9 (2CH_Ar_), 115.5 (CH_Ar_), 105.1 (C), 87.8 (C), 46.8 (CH_2_), 42.3 (CH_2_), and 26.8 (CH_3_). Analysis calculated for C_25_H_23_ClN_4_O_3_: HRMS: *m*/*z* [M + H]^+^ calculated 463.1531; found 463.1531.

*5-Benzyl-2-(4-(N′-hydroxycarbamimidoyl)phenyl)-5-methyl-N-(5-methylpyridin-2-yl)-4,5-dihydrofuran-3-carboxamide* (**16**).

The compound **16** was synthesized following the previously described procedure from 5 (85 mg, 0.21 mmol, and 1 equiv.). The reaction progress was monitored by TLC (eluent: cyclohexane/AcOEt/dichloromethane 4/3/3; Rf of 0.23). The crude product was purified by flash chromatography using a PuriFlash^®^ IR-50SI-F0025 column and eluted with cyclohexane/AcOEt in a gradient from 100/0 to 70/30 over 25 column volumes. A UV–VIS detector was configured additionally to 222 nm and 230 nm. Yield: 85%. The product was obtained as a light-yellow oil. ^1^H NMR (400 MHz, CDCl_3_): δ (ppm) 8.22–8.10 (m, 2H, 2CH_Ar_), 8.04 (br s, 1H, NH), 7.74 (d, ^3^*J*_H-H_ = 8.5 Hz, 2H, 2CH_Ar_), 7.68 (d, ^3^*J*_H-H_ = 8.5 Hz, 2H, 2CH_Ar_), 7.49 (d, ^3^*J*_H-H_ = 8.8 Hz, 1H, CH_Ar_), 7.32–7.18 (m, 5H, 5CH_Ar_), 4.98 (br s, 2H, NH_2_), 3.15 (d, ^2^*J*_H-H_ = 14.4 Hz, 1H, H-(CH_2_)), 2.96 (d, ^2^*J*_H-H_ = 13.8 Hz, 1H, H-(CH_2_)), 2.88 (d, ^2^*J*_H-H_ = 13.4 Hz, 1H, H-(CH_2_)), 2.86 (d, ^2^*J*_H-H_ = 14.4 Hz, 1H, H-(CH_2_)), 2.28 (s, 3H, CH_3_), and 1.36 (s, 3H, CH_3_). OH not observed. ^13^C NMR (100 MHz, CDCl_3_): δ (ppm) 163.6 (C), 160.9 (C), 152.3 (C), 149.5 (C), 147.1 (CH_Ar_), 139.3 (CH_Ar_), 136.4 (C), 134.2 (C), 131.9 (C), 130.5 (2CH_Ar_), 129.5 (2CH_Ar_), 128.8 (C), 128.3 (2CH_Ar_), 126.9 (CH_Ar_), 125.8 (2CH_Ar_), 114.2 (CH_Ar_), 105.3 (C), 87.4 (C), 46.7 (CH_2_), 42.6 (CH_2_), 26.5 (CH_3_), and 17.9 (CH_3_). Analysis calculated for C_26_H_26_N_4_O_3_: HRMS: *m*/*z* [M + H]^+^ calculated 443.2078; found 443.2076.

*5-Benzyl-2-(4-(N′-hydroxycarbamimidoyl)phenyl)-5-methyl-N-(pyridin-2-yl)-4,5-dihydrofuran-3-carboxamide* (**17**).

The compound **17** was synthesized following the previously described procedure from 5 (74 mg, 0.19 mmol, and 1 equiv.). The reaction progress was monitored by TLC (eluent: dichloromethane/MeOH 97/3; Rf of 0.23). The crude product was purified by flash chromatography using a PuriFlash^®^ IR-50SI-F0025 column and eluted with dichloromethane/MeOH in a gradient from 100/0 to 98/2 over 25 column volumes. A UV–VIS detector was configured additionally to 222 nm and 230 nm. Yield: 55%. The product was obtained as a light-gray oil. ^1^H NMR (400 MHz, CDCl_3_): δ (ppm) 8.46 (br s, 1H, NH), 7.23 (d, ^3^*J*_H-H_ = 8.6 Hz, 1H, CH_Ar_), 8.17–8.14 (m, 1H, CH_Ar_), 7.75–7.62 (m, 5H, 5CH_Ar_), 7.31–7.19 (m, 5H, 5CH_Ar_), 7.04–6.97 (m, 1H, CH_Ar_), 5.65 (br s, 2H, NH_2_), 3.24 (d, ^2^*J*_H-H_ = 14.4 Hz, 1H, H-(CH_2_)), 3.01 (d, ^2^*J*_H-H_ = 14.2 Hz, 1H, H-(CH_2_)), 2.98 (d, ^2^*J*_H-H_ = 14.2 Hz, 1H, H-(CH_2_)), 2.96 (d, ^2^*J*_H-H_ = 14.4 Hz, 1H, H-(CH_2_)), and 1.45 (s, 3H, CH_3_). OH not observed. ^13^C NMR (100 MHz, CDCl_3_): δ (ppm) 163.8 (C), 161.9 (C), 157.3 (C), 151.2 (C), 141.7 (C), 139.3 (CH_Ar_), 136.3 (C), 132.3 (C), 130.6 (2CH_Ar_), 129.7 (2CH_Ar_), 128.4 (2CH_Ar_), 127.0 (CH_Ar_), 126.8 (CH_Ar_), 126.3 (2CH_Ar_), 119.5 (CH_Ar_), 115.1 (CH_Ar_), 105.2 (C), 88.1 (C), 46.8 (CH_2_), 42.3 (CH_2_), and 26.8 (CH_3_). Analysis calculated for C_25_H_24_N_4_O_3_: HRMS: *m*/*z* [M + H]^+^ calculated 429.1921; found 429.1925.

*5-Benzyl-2-(4-(N′-hydroxycarbamimidoyl)phenyl)-5-methyl-N-(pyridin-4-yl)-4,5-dihydrofuran-3-carboxamide* (**18**).

The compound **18** was synthesized following the previously described procedure from 6 (67 mg, 0.17 mmol, and 1 equiv.). The reaction progress was monitored by TLC (eluent: dichloromethane/methanol 95/5; Rf of 0.19). The crude product was purified by flash chromatography using a PuriFlash^®^ IR-50SI-F0025 column and eluted with dichloromethane/MeOH in a gradient 100/0 to 96/4 over 25 column volumes. A UV–VIS detector was configured to 254 nm, 375 nm, and 327 nm. Yield: 70%. The product was obtained as a white oil. ^1^H NMR (400 MHz, CDCl_3_): δ (ppm) 8.34 (d, ^3^*J*_H-H_ = 6.4 Hz, 2H, 2CH_Ar_), 7.72 (d, ^3^*J*_H-H_ = 8.5 Hz, 2H, 2CH_Ar_), 7.59 (d, ^3^*J*_H-H_ = 8.5 Hz, 2H, 2CH_Ar_), 7.34–7.22 (m, 5H, 5CH_Ar_), 7.17 (d, ^3^*J*_H-H_ = 6.4 Hz, 2H, 2CH_Ar_), 7.08 (br s, 1H, NH), 4.95 (br s, 2H, NH_2_), 3.19 (d, ^2^*J*_H-H_ = 14.8 Hz, 1H, H-(CH_2_), 3.06 (d, ^2^*J*_H-H_ = 13.8 Hz, 1H, H-(CH_2_), 3.01 (d, ^2^*J*_H-H_ = 13.8 Hz, 1H, H-(CH_2_), 2.93 (d, ^2^*J*_H-H_ = 14.8 Hz, 1H, H-(CH_2_)), and 1.52 (s, 3H, CH_3_). OH not observed. ^13^C NMR (100 MHz, CDCl_3_): δ (ppm) 163.8 (C), 160.0 (C), 151.7 (C), 150.3 (2CH_Ar_), 145.3 (C), 136.4 (C), 135.1 (C), 131.3 (C), 130.6 (2CH_Ar_), 129.3 (2CH_Ar_), 128.4 (2CH_Ar_), 127.0 (CH_Ar_), 126.2 (2CH_Ar_), 113.3 (2CH_Ar_), 106.5 (C), 88.1 (C), 46.9 (CH_2_), 42.3 (CH_2_), and 27.0 (CH_3_). Analysis calculated for C_25_H_24_N_4_O_3_: HRMS: *m*/*z* [M + H]^+^ calculated 429.1921; found 419.1920.

*5-Benzyl-2-(4-(N′-hydroxycarbamimidoyl)phenyl)-5-methyl-N-phenyl-4,5-dihydrofuran-3-carboxamide* (**19**).

The compound **19** was synthesized following the previously described procedure from 7 (60 mg, 0.15 mmol, and 1 equiv.). The reaction progress was monitored by TLC (eluent: dichloromethane/MeOH 96/4; Rf of 0.47). The crude product was purified by column chromatography (eluent: dichloromethane/MeOH 98/2). Yield: 44%. The product was obtained as an oily yellow solid. ^1^H NMR (400 MHz, CDCl_3_): δ (ppm) 7.69 (d, ^3^*J*_H-H_ = 8.4 Hz, 2H, 2CH_Ar_), 7.67 (d, ^3^*J*_H-H_ = 8.4 Hz, 2H, 2CH_Ar_), 7.32–7.22 (m, 9H, 9CH_Ar_), 7.04 (t, ^3^*J*_H-H_ = 6.9 Hz, 1H, CH_Ar_), 6.83 (br s, 1H, NH), 4.96 (s, 2H, NH_2_), 3.19 (d, ^2^*J*_H-H_ = 14.8 Hz, 1H, H-(CH_2_), 3.06 (d, ^2^*J*_H-H_ = 13.8 Hz, 1H, H-(CH_2_), 3.03 (d, ^2^*J*_H-H_ = 13.8 Hz, 1H, H-(CH_2_), 2.95 (d, ^2^*J*_H-H_ = 14.8 Hz, 1H, H-(CH_2_), and 1.53 (s, 3H, CH_3_). OH not observed. ^13^C NMR (100 MHz, CDCl_3_): δ (ppm) 163.5 (C), 158.3 (C), 152.1 (C), 138.0 (C), 136.6 (C), 134.1 (C), 131.8 (C), 130.6 (2CH_Ar_), 129.4 (2CH_Ar_), 129.1 (2CH_Ar_), 128.3 (2CH_Ar_), 126.9 (CH_Ar_), 126.0 (2CH_Ar_), 124.0 (CH_Ar_), 119.6 (2CH_Ar_), 106.7 (C), 87.4 (C), 47.0 (CH_2_), 42.7 (CH_2_), and 27.0 (CH_3_). Analysis calculated for C_26_H_25_N_3_O_3_: HRMS: *m*/*z* [M + H]^+^ calculated 428.1969; found 428.1967.

*5-Benzyl-N-(4-fluorophenyl)-2-(4-(N′-hydroxycarbamimidoyl)phenyl)-5-methyl-4,5-dihydrofuran-3-carboxamide* (**20**).

The compound **20** was synthesized following the previously described procedure from 8 (70 mg, 0.17 mmol, and 1 equiv.). The reaction progress was monitored by TLC (eluent: dichloromethane/MeOH 96/4; Rf of 0.47). The crude product was purified by column chromatography (eluent: dichloromethane/MeOH 98/2). Yield: 49%. The product was obtained as an oily brown solid. ^1^H NMR (400 MHz, CDCl_3_): δ (ppm) 7.70–7.59 (m, 4H, 4CH_Ar_), 7.29–7.17 (m, 7H, 7CH_Ar_), 6.91 (t, ^3^*J*_H-H_ = 8.6 Hz, 2H, 2CH_Ar_), 6.73 (br s, 1H, NH), 4.92 (br s, 2H, NH_2_), 3.15 (d, ^2^*J*_H-H_ = 14.8 Hz, 1H, H-(CH_2_), 3.04 (d, ^2^*J*_H-H_ = 13.9 Hz, 1H, H-(CH_2_), 2.99 (d, ^2^*J*_H-H_ = 13.9 Hz, 1H, H-(CH_2_), 2.91 (d, ^2^*J*_H-H_ = 14.8 Hz, 1H, H-(CH_2_), and 1.50 (s, 3H, CH_3_). OH not observed. ^13^C NMR (100 MHz, CDCl_3_): δ (ppm) 163.4 (C), 159.3 (d, *J_C-F_* = 243.0 Hz, C), 158.6 (C), 158.1 (C), 136.6 (C), 134.2 (C), 134.0 (d, *J_C-F_* = 2.8 Hz, C), 131.8 (C), 130.6 (2CH_Ar_), 129.4 (2CH_Ar_), 128.4 (2CH_Ar_), 127.0 (CH_Ar_), 126.0 (2CH_Ar_), 121.4 (d, *J_C-F_* = 7.7 Hz, 2CH_Ar_), 115.7 (d, *J_C-F_* = 22.5 Hz, 2CH_Ar_), 106.4 (C), 87.5 (C), 47.0 (CH_2_), 42.7 (CH_2_), and 27.0 (CH_3_). ^19^F NMR (376.5 MHz, CDCl_3_): δ (ppm) −104.5. Analysis calculated for C_26_H_24_FN_3_O_3_: HRMS: *m*/*z* [M + H]^+^ calculated 446.1874; found 446.1874.

*5-Benzyl-2-(4-(N′-hydroxycarbamimidoyl)phenyl)-5-methyl-N-(4-(trifluoromethyl)benzyl)-4,5-dihydrofuran-3-carboxamide* (**21**).

The compound **21** was synthesized following the previously described procedure from 9 (70 mg, 0.15 mmol, and 1 equiv.). The reaction progress was monitored by TLC (eluent: cyclohexane/AcOEt 70/30; Rf of 0.21). The crude product was purified by flash chromatography using a PuriFlash^®^ IR-50SI-F0025 column and eluted with cyclohexane/AcOEt in a gradient from 100/0 to 70/30 over 25 column volumes. A UV–VIS detector configured additionally to 225 nm and 230 nm. Yield: 57%. The product was obtained as a brown oil. ^1^H NMR (400 MHz, CDCl_3_): δ (ppm) 7.65–7.52 (m, 6H, 6CH_Ar_), 7.33–7.16 (m, 7H, 7CH_Ar_), 5.42 (t, ^3^*J*_H-H_ = 5.8 Hz, 1H, NH), 4.87 (br s, 2H, NH_2_), 4.46–4.35 (m, 2H, CH_2_), 3.09 (d, ^2^*J*_H-H_ = 14.7 Hz, 1H, H-(CH_2_)), 3.03 (d, ^2^*J*_H-H_ = 13.8 Hz, 1H, H-(CH_2_)), 2.99 (d, ^2^*J*_H-H_ = 13.8 Hz, 1H, H-(CH_2_)), 2.85 (d, ^2^*J*_H-H_ = 14.7 Hz, 1H, H-(CH_2_)), and 1.49 (s, 3H, CH_3_). OH not observed. ^13^C NMR (100 MHz, CDCl_3_): δ (ppm) 165.4 (C), 158.5 (C), 152.0 (C), 142.6 (C), 136.6 (C), 134.1 (C), 131.9 (C), 130.5 (2CH_Ar_), 129.3 (2CH_Ar_), 129.7 (q, ^2^*J_C-F_* = 32.5 Hz, C), 128.3 (2CH_Ar_), 128.1 (2CH_Ar_), 126.9 (CH_Ar_), 125.7 (2CH_Ar_), 125.6 (q, ^3^*J_C-F_* = 3.7 Hz, 2CH_Ar_), 124.5 (q, ^1^*J_C-F_* = 271.5 Hz, CF_3_), 105.4 (C), 82.7 (C), 46.9 (CH_2_), 42.9 (CH_2_), 42.7 (CH_2_), and 26.9 (CH_3_). ^19^F NMR (376.5 MHz, CDCl_3_): δ (ppm) -63.1. Analysis calculated for C_28_H_26_F_3_N_3_O_3_: HRMS: *m*/*z* [M + H]^+^ calculated 510.1999; found 510.1997.

*5-Benzyl-2-(4-(N′-hydroxycarbamimidoyl)phenyl)-5-methyl-N-(pyridazin-3-yl)-4,5-dihydrofuran-3-carboxamide* (**22**).

The compound **22** was synthesized following the previously described procedure from 10 (62 mg, 0.16 mmol, and 1 equiv.). The reaction progress was monitored by TLC (eluent: dichloromethane/MeOH 95/5; Rf of 0.32). The crude product was purified by flash chromatography using a PuriFlash^®^ IR-50SI-F0025 column and eluted with dichloromethane/MeOH in a gradient from 100/0 to 98/2 over 25 column volumes. A UV–VIS detector was configured to 241 nm, 212 nm, and 315 nm. Yield: 60%. The product was obtained as a light-yellow oil. ^1^H NMR (400 MHz, CDCl_3_): δ (ppm) 8.80 (d, ^3^*J*_H-H_ = 5.2 Hz, 1H, CH_Ar_), 8.53 (br s, 1H, NH), 8.40 (d, ^3^*J*_H-H_ = 9.6 Hz, 1H, CH_Ar_), 7.67 (d, ^3^*J*_H-H_ = 8.8 Hz, 2H, 2CH_Ar_), 7.67 (d, ^3^*J*_H-H_ = 8.8 Hz, 2H, 2CH_Ar_), 7.38 (dd, ^3^*J*_H-H_ = 9.6 Hz, ^3^*J*_H-H_ = 5.2 Hz, 1H, CH_Ar_), 7.28–7.19 (m, 5H, 5CH_Ar_), 5.17 (br s, 2H, NH_2_), 3.19 (d, ^2^*J*_H-H_ = 14.4 Hz, 1H, H-(CH_2_)), 3.00 (d, ^2^*J*_H-H_ = 14.3 Hz, 1H, H-(CH_2_)), 2.96 (d, ^2^*J*_H-H_ = 14.3 Hz, 1H, H-(CH_2_)), 2.90 (d, ^2^*J*_H-H_ = 14.4 Hz, 1H, H-(CH_2_)), and 1.43 (s, 3H, CH_3_). OH not observed. ^13^C NMR (100 MHz, CDCl_3_): δ (ppm) 164.0 (C), 162.2 (C), 155.5 (C), 152.4 (C), 148.1 (CH_Ar_), 136.2 (C), 134.5 (C), 131.5 (C), 130.5 (2CH_Ar_), 129.4 (2CH_Ar_), 128.4 (3CH_Ar_), 127.0 (CH_Ar_), 126.1 (2CH_Ar_), 119.2 (CH_Ar_), 104.9 (C), 88.0 (C), 46.7 (CH_2_), 42.1 (CH_2_), and 27.6 (CH_3_). Analysis calculated for C_24_H_23_N_5_O_3_: HRMS: *m*/*z* [M + H]^+^ calculated 430.1874; found 430.1875.

*5-Benzyl-N-(6-chloropyridazin-3-yl)-2-(4-(N′-hydroxycarbamimidoyl)phenyl)-5-methyl-4,5-dihydrofuran-3-carboxamide* (**23**).

The compound **23** was synthesized following the previously described procedure from 11 (55 mg, 0.33 mmol, and 1 equiv.). The reaction progress was monitored by TLC (eluent: AcOEt/cyclohexane/dichloromethane 4/3/3; Rf of 0.19). The crude product was purified by flash chromatography using a PuriFlash^®^ IR-50SI-F0025 column and eluted with AcOEt/cyclohexane in a gradient from 10/90 to 60/40 over 25 column volumes. A UV–VIS detector was configured to 241 nm, 212 nm, and 315 nm. Yield: 83%. The product was obtained as a light-brown oil. ^1^H NMR (400 MHz, CDCl_3_): δ (ppm) 8.65 (s, 1H, NH), 8.47 (d, ^3^*J*_H-H_ = 9.2 Hz, 1H, CH_Ar_), 7.66 (s, 4H, 4CH_Ar_), 7.42 (d, ^3^*J*_H-H_ = 9.2 Hz, 1H, CH_Ar_), 7.30–7.20 (m, 5H, 5CH_Ar_), 5.08 (br s, 2H, NH_2_), 3.19 (d, ^2^*J*_H-H_ = 14.4 Hz, 1H, H-(CH_2_)), 3.00 (s, 2H, CH_2_), 2.90 (d, ^2^*J*_H-H_ = 14.4 Hz, 1H, H-(CH_2_)), and 1.46 (s, 3H, CH_3_). OH not observed. ^13^C NMR (100 MHz, CDCl_3_): δ (ppm) 163.8 (C), 162.6 (C), 154.6 (C), 151.9 (C), 148.2 (C), 136.2 (C), 134.0 (C), 132.0 (C), 130.7 (2CH_Ar_), 130.0 (CH_Ar_), 129.6 (2CH_Ar_), 128.5 (2CH_Ar_), 127.2 (2CH_Ar_), 126.4 (CH_Ar_), 121.5 (CH_Ar_), 105.0 (C), 88.4 (C), 46.8 (CH_2_), 42.0 (CH_2_), and 26.9 (CH_3_). Analysis calculated for C_24_H_22_ClN_5_O_3_: HRMS: *m*/*z* [M + H]^+^ calculated 464.1484; found 464.1484.

*5-Benzyl-2-(4-(N′-hydroxycarbamimidoyl)phenyl)-5-methyl-N-(pyrazin-2-yl)-4,5-dihydrofuran-3-carboxamide* (**24**).

The compound **24** was synthesized following the previously described procedure from 12 (77 mg, 0.19 mmol, and 1 equiv.). The reaction progress was monitored by TLC (eluent: cyclohexane/AcOEt/dichloromethane 30/40/30; Rf of 0.15). The crude product was purified by column chromatography (eluent: cyclohexane/ethyl acetate/dichloromethane 30/40/30). Yield: 59%. The product was obtained as an oily yellow solid. ^1^H NMR (400 MHz, CDCl_3_): δ (ppm) 9.50 (s, 1H, NH), 8.27 (d, ^3^*J*_H-H_ = 2.7 Hz, 1H, CH_Ar_), 8.14 (d, ^3^*J*_H-H_ = 2.7 Hz, 1H, CH_Ar_), 7.78 (s, 1H, CH_Ar_), 7.70 (s, 4H, 4CH_Ar_), 7.31–7.24 (m, 5H, 5CH_Ar_), 5.14 (br s, 2H, NH_2_), 3.20 (d, ^2^*J*_H-H_ = 14.4 Hz, 1H, H-(CH_2_)), 3.02 (s, 2H, CH_2_), 2.91 (d, ^2^*J*_H-H_ = 14.4 Hz, 1H, H-(CH_2_)), and 1.48 (s, 3H, CH_3_). OH not observed. ^13^C NMR (100 MHz, CDCl_3_): δ (ppm) 163.2 (C), 161.9 (C), 152.4 (C), 148.4 (C), 141.9 (CH_Ar_), 139.7 (CH_Ar_), 137.3 (CH_Ar_), 136.2 (C), 134.2 (C), 131.7 (C), 130.5 (2CH_Ar_), 129.6 (2CH_Ar_), 128.4 (2CH_Ar_), 127.1 (CH_Ar_), 126.1 (2CH_Ar_), 104.9 (C), 88.0 (C), 46.8 (CH_2_), 42.3 (CH_2_), and 26.8 (CH_3_). Analysis calculated for C_24_H_23_N_5_O_3_: HRMS: *m*/*z* [M + H]^+^ calculated 430.1874; found 430.1871.

*5-Benzyl-N-(1,5-dimethyl-1H-pyrazol-3-yl)-2-(4-(N′-hydroxycarbamimidoyl)phenyl)-5-methyl-4,5-dihydrofuran-3-carboxamide* (**25**).

The compound **25** was synthesized following the previously described procedure from 13 (88 mg, 0.21 mmol, and 1 equiv.). The reaction progress was monitored by TLC (eluent: dichloromethane/MeOH 30/40/30; Rf of 0.31). The crude product was purified by flash chromatography using a PuriFlash^®^ IR-50SI-F0025 column and eluted with dichloromethane/MeOH in a gradient from 100/0 to 90/10 over 25 column volumes. A UV–VIS detector was configured at 238 nm and 320 nm. Yield: 60%. The product was obtained as a light-brown oil. ^1^H NMR (400 MHz, CDCl_3_): δ (ppm) 7.90 (s, 1H, NH), 7.71 (d, ^3^*J*_H-H_ = 8.4 Hz, 2H, 2CH_Ar_), 7.64 (d, ^3^*J*_H-H_ = 8.4 Hz, 2H, 2CH_Ar_), 7.30–7.18 (m, 5H, 5CH_Ar_), 6.47 (s, 1H, CH_Ar_), 5.00 (br s, 2H, NH_2_), 3.63 (s, 3H, CH_3_), 3.06 (d, ^2^*J*_H-H_ = 14.5 Hz, 1H, H-(CH_2_)), 2.93 (d, ^2^*J*_H-H_ = 13.7 Hz, 1H, H-(CH_2_)), 2.86 (d, ^2^*J*_H-H_ = 13.7 Hz, 1H, H-(CH_2_)), 2.78 (d, ^2^*J*_H-H_ = 14.5 Hz, 1H, H-(CH_2_)), 2.21 (s, 3H, CH_3_), and 1.33 (s, 3H, CH_3_). OH not observed. ^13^C NMR (100 MHz, CDCl_3_): δ (ppm) 162.5 (C), 159.7 (C), 152.2 (C), 145.8 (C), 139.5 (C), 136.5 (C), 134.2 (C), 131.7 (C), 130.5 (2CH_Ar_), 129.3 (2CH_Ar_), 128.2 (2CH_Ar_), 126.8 (CH_Ar_), 125.7 (2CH_Ar_), 105.0 (C), 97.5 (CH_Ar_), 87.0 (C), 46.6 (CH_2_), 42.7 (CH_2_), 35.5 (CH_3_), 26.5 (CH_3_), and 11.4 (CH_3_). Analysis calculated for C_25_H_27_N_5_O_3_: HRMS: *m*/*z* [M + H]^+^ calculated 446.2187; found 446.2187.

### 3.2. Biology

#### 3.2.1. Parasite Cultivation

*Leishmania amazonensis* (MHOM/BR/77/LTB0016) was cultured as promastigotes at 26 °C using Schneider’s insect medium (Sigma-Aldrich, St Louis, MO, USA), supplemented with 10% heat-inactivated fetal calf serum (HIFCS), 100 μg/mL streptomycin, and 100 U/mL penicillin. The parasites were sustained up to the 10th passage, after which fresh cultures were derived from infected host animals.

#### 3.2.2. Antipromastigote Activity Assay

*L. amazonensis* promastigotes were cultured in Schneider’s insect medium with 10% HIFCS, as described above, with or without various concentrations of test substances. The initial cell density was 1.0 × 10^6^ cells/mL, and the culture was kept at 26 °C for 72 h. Resazurin reduction was used to evaluate the cell viability. The concentration required to inhibit 50% of the promastigote activity (IC_50_) was calculated using logarithmic regression analysis via GraphPrism 5 software.

#### 3.2.3. Cytotoxicity Assay

Murine peritoneal macrophages were collected by flushing the peritoneal cavity with 5 mL of RPMI 1640 medium containing 10% HIFCS, 2 mM L-glutamine, 1 mM pyruvate, 100 μg/mL streptomycin, and 100 U/mL penicillin. The macrophage suspension was adjusted to 1.0 × 10^6^ cells/mL and then plated in triplicate in 96-well plates (Falcon Co., Franklin Lakes, NJ, USA). Cultures were incubated at 37 °C in a 5% CO_2_ environment for 1 h. Nonadherent cells were subsequently removed, and the remaining cells were exposed to varying concentrations of test compounds (0–200 μM) for 72 h. Viability was assessed using a resazurin assay, and the concentration that caused 50% cytotoxicity (CC_50_) was determined using logarithmic regression analysis with GraphPrism 5 software.

#### 3.2.4. Antiamastigote Activity Assay

Resident peritoneal macrophages were seeded in RPMI medium (Sigma-Aldrich) at 1 × 10^6^ cells/mL (0.4 mL/well) on Lab-Tek eight-chamber slides (Nunc) and incubated at 37 °C in 5% CO_2_ for 1 h. After this period, nonadherent cells were removed, and the adherent macrophages were co-cultured with *L. amazonensis* promastigotes at a 5:1 parasite-to-cell ratio. After 4 h, the monolayers were washed to eliminate free parasites. Cells were then treated with different concentrations of the compounds and incubated for 72 h at 37 °C in 5% CO_2_. Following incubation, the slides were stained using the Instant Prov hematological dye (Newprov, Curitiba, Brazil), and antileishmanial activity was assessed microscopically. Amastigote numbers were determined by counting at least 100 macrophages per sample, and the results were expressed as an infection index (II), calculated as: II = (percentage of infected cells) × (number of amastigotes/total macrophages). Dose–response curves for the antiamastigote activity were generated using nonlinear regression in GraphPad Prism 5.0, and IC_50_ values were calculated.

## 4. Conclusions

A Hit-to-Lead process enhanced the antileishmanial properties of the 4,5-dihydrofuran derivatives bearing an amidoxime group. While the activity against the promastigote form of the parasite was moderate (with IC_50_ values ranging from 15 to 52 μM), three of the assessed 4,5-dihydrofuran-3-carboxamide derivatives displayed significantly improved activity against the intracellular amastigote form of the parasite (with IC_50_ values between 0.3 and 0.6 μM), which is relevant given that it is the infective form of the parasite. Notably, pyridine-type derivatives demonstrated greater enhancement of the antileishmanial activity compared to the other nitrogen-containing assessed compounds. The improved antiamastigote activity may suggest a hypothetical balance between lipophilicity and solubility, modulated by the varying pH environments of the parasite–macrophage interaction, thus warranting further investigation. Moreover, the incorporation of the pyridine moiety not only enhanced the biological activity but also provided a promising toxicity/activity ratio, with a selectivity index (SI) greater than 300. The wide range of concentrations at which these new potential lead compounds remain non-toxic makes them interesting for further modulations and in vivo testing, especially compound **16**, with better predicted parameters for biological activity and biopharmaceuticals.

## Figures and Tables

**Table 1:** Synthesis of 4,5-dihydrofuran-3-carboxamide intermediates and amidoximes from nitriles.

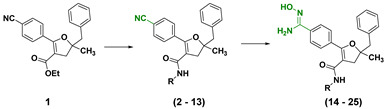				
**R**	**Nitrile Intermediate**		**Amidoxime Product**	
	**Compound**	**Yield (%)**	**Compound**	**Yield (%)**
-5-bromopyridin-2-yl	**2**	45	**14**	68
-5-chloropyridin-2-yl	**3**	48	**15**	55
-5-methylpyridin-2-yl	**4**	66	**16**	85
-pyridin-2-yl	**5**	85	**17**	55
-pyridin-4-yl	**6**	50	**18**	70
-phenyl	**7**	41	**19**	44
-4-fluorophenyl	**8**	68	**20**	49
-4-(trifluoromethyl)benzyl	**9**	47	**21**	57
-pyridazin-3-yl	**10**	43	**22**	60
-6-chloropyridazin-3-yl	**11**	59	**23**	83
-pyrazin-2-yl	**12**	59	**24**	59
-1,5-dimethyl-1*H*-pyrazol-3-yl	**13**	66	**25**	60

**Table 2:** Antileishmanial activity of the 4,5-dihydrofuran-3-carboxamide derivatives on the promastigote form of *L. amazonensis*.

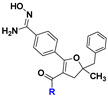	**Compound**	**Structure**	**Activity IC_50_ (µM/72 h) ^a^**	**Compound**	**Structure**	**Activity IC_50_ (µM/72 h) ^a^**
	**14**	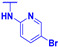	15.0 ± 1.03	**20**	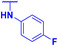	23.8 ± 1.02
	**15**	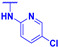	16.0 ± 1.03	**21**	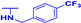	26.4 ± 1.02
	**16**	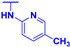	17.0 ± 1.02	**22**	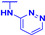	29.9 ± 1.02
	**17**	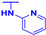	22.4 ± 1.02	**23**	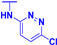	36.4 ± 1.03
	**18**	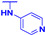	23.5 ± 1.04	**24**	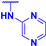	39.4 ± 1.02
	**19**	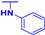	23.8 ± 1.02	**25**	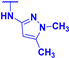	52.5 ± 1.03

^a^ The IC_50_ (µM/72 h) values represent the mean ± SD from three independent experiments.

**Table 3:** Antileishmanial activity, cytotoxicity, and selectivity index of the 4,5-dihydrofuran-3-carboxamide carrying an amidoxime group.

Compound	Structure	Cytotoxicity CC_50_ (µM/72 h) ^a^	Intracellular Amastigotes IC_50_ (µM/72 h) ^a^	SI = CC_50_/IC_50_ Intracellular Amastigotes
**14**	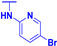	171.5 ± 1.19	0.5 ± 0.15	343
**15**	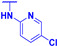	>200	0.6 ± 0.20	>333.3
**16**	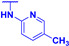	>200	0.3 ± 0.07	>666.7

^a^ Values represent the mean ± SD from three independent experiments.

## Data Availability

Data are contained within the article and Supplementary Materials.

## Supplementary Materials

The following supporting information can be downloaded at: https://www.mdpi.com/article/10.3390/molecules29225469/s1, NMR spectra.
