# Supplementary material for: Enhancing Antileishmanial Activity of Amidoxime-Based Compounds Bearing a 4,5-Dihydrofuran Scaffold: In Vitro Screening Against Leishmania amazonensis
Source: Molecules. 2024 Nov 20;29(22):5469. doi: 10.3390/molecules29225469 (PMC11597885; doi:10.3390/molecules29225469)

Supporting Information

Enhancing Antileishmanial Activity of Amidoxime-Based Compounds Bearing a 4,5-Dihydrofuran Scaffold: In Vitro Screening Against *Leishmania amazonensis*

Fabiana Maia Santos Urbancg Moncorvo [1,†], Oscar Leonardo Avendaño Leon [2,†], Christophe Curti [2,3], Youssef Kabri [2], Sébastien Redon [2], Eduardo Caio Torres-Santos [1,*] and Patrice Vanelle [2,3,*]

1 Laboratório de Bioquímica de Tripanosomatídeos, Instituto Oswaldo Cruz—FIOCRUZ, Av. Brasil, 4365,
Rio de Janeiro 21040-900, Brazil; fabianamaia.santos@gmail.com

2 Aix Marseille Univ, CNRS, ICR UMR 7273, Equipe Pharmaco-Chimie Radicalaire, Faculté de Pharmacie, 27 Boulevard Jean Moulin, CS30064, CEDEX 05, 13385 Marseille, France;
oscar-leonardo.avendano-leon@etu.univ-amu.fr (O.L.A.L.); christophe.curti@univ-amu.fr (C.C.);
youssef.kabri@univ-amu.fr (Y.K.); sebastien.redon@univ-amu.fr (S.R.)

3 Service Central de la Qualité et de l'Information Pharmaceutiques (SCQIP), Pharmacy Department,
Assistance Publique—Hôpitaux de Marseille (AP-HM), 147 Bd Baille, 13006, Marseille, France

* Correspondence: ects@ioc.fiocruz.br (E.C.T.-S.); patrice.vanelle@univ-amu.fr (P.V.);
Tel.: +55-21-987476937 (E.C.T.-S.); +33-4-9183-5580 (P.V.)

† These authors contributed equally to this work.

Ethyl 5-benzyl-2-(4-cyanophenyl)-5-methyl-4,5-dihydrofuran-3-carboxylate (1) - $C_{22}H_{21}NO_3$.

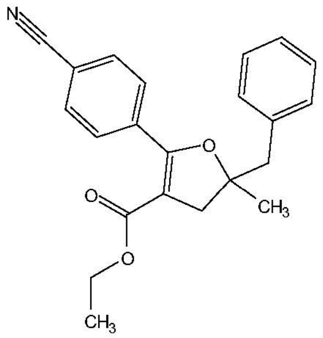

^{1}H NMR (400 MHz, $CDCl_3$): δ (ppm) 7.85 (d, $^3J_{H\text{-}H}$ = 8.6 Hz, 2H, $2CH_{Ar}$), 7.64 (d, $^3J_{H\text{-}H}$ = 8.6 Hz, 2H, $2CH_{Ar}$), 7.30 -7.19 (m, 5H, $5CH_{Ar}$), 4.08 (q, $^3J_{H\text{-}H}$ = 7.1 Hz, 2H, CH_2), 3.10 (d, $^2J_{H\text{-}H}$ = 15.5 Hz, 1H, H-(CH_2)), 3.02 (d, $^2J_{H\text{-}H}$ = 15.3 Hz, 1H, H-(CH_2)), 2.98 (d, $^2J_{H\text{-}H}$ = 15.3 Hz, 1H, H-(CH_2)), 2.82 (d, $^2J_{H\text{-}H}$ = 15.5 Hz, 1H, H-(CH_2)), 1.45 (s, 3H, CH_3), 1.17 (t, $^3J_{H\text{-}H}$ = 7.1 Hz, 3H, CH_3).

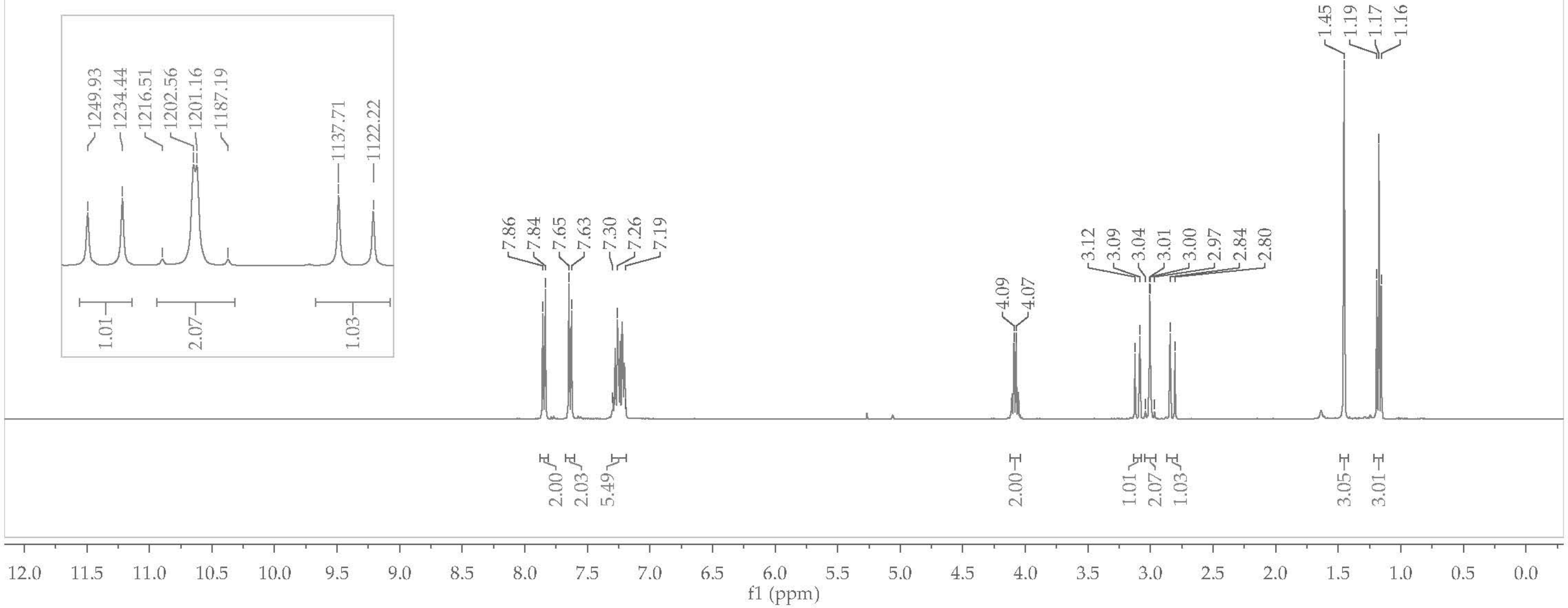

Ethyl 5-benzyl-2-(4-cyanophenyl)-5-methyl-4,5-dihydrofuran-3-carboxylate (1) - $C_{22}H_{21}NO_3$.

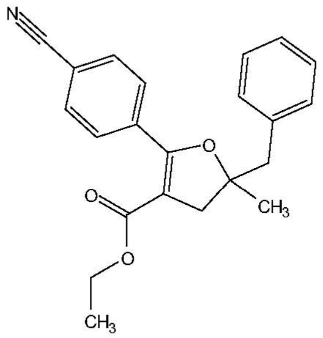

^{13}C NMR (100 MHz, $CDCl_3$): δ (ppm) 164.6 (C), 161.0 (C), 136.1 (C), 134.5 (C), 131.1 ($2CH_{Ar}$), 130.2 ($2CH_{Ar}$), 129.8 ($2CH_{Ar}$), 128.0 ($2CH_{Ar}$), 126.6 (CH_{Ar}), 118.3 (C), 113.2 (C), 104.2 (C), 87.8 (C), 59.7 (CH_2), 46.5 (CH_2), 42.1 (CH_2), 26.4 (CH_3), 14.0 (CH_3).

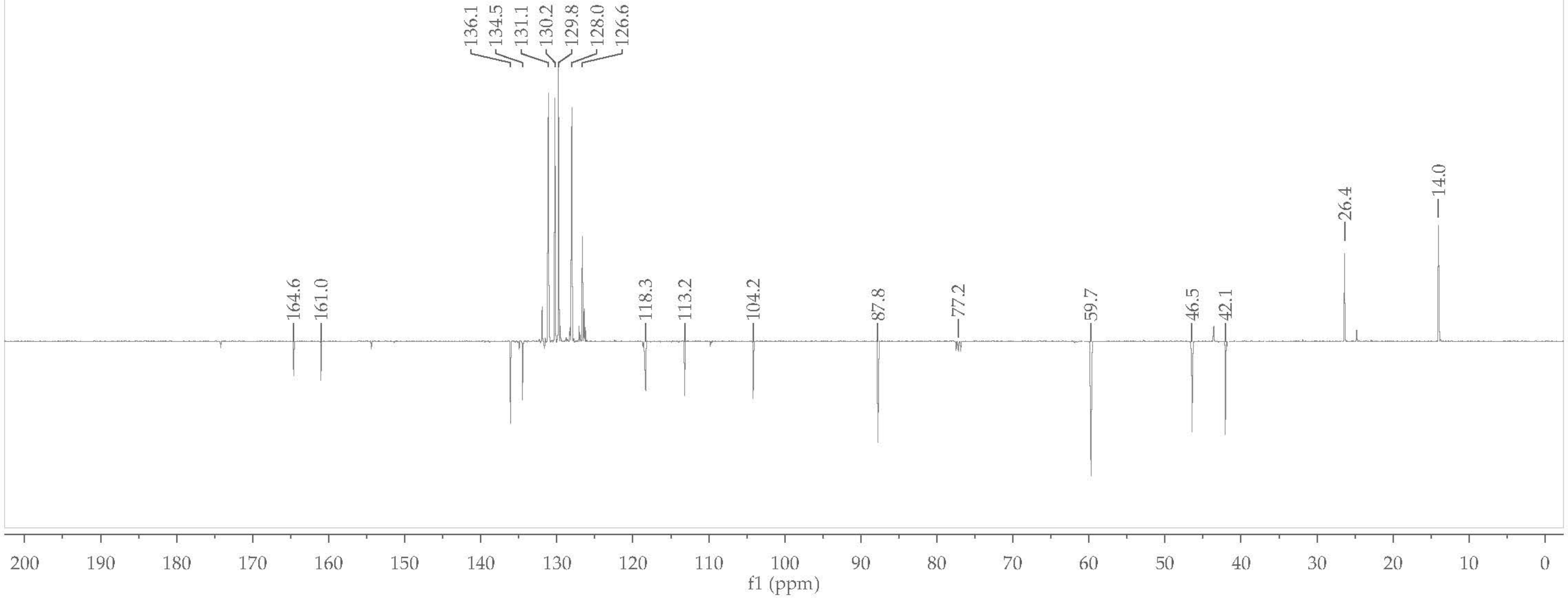

5-Benzyl-*N*-(5-bromopyridin-2-yl)-2-(4-cyanophenyl)-5-methyl-4,5-dihydrofuran-3-carboxamide (2) - $C_{25}H_{20}BrN_3O_2$

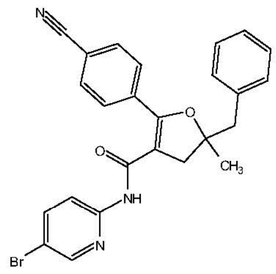

^{1}H NMR (400 MHz, $CDCl_3$): δ (ppm) 8.24 (d, $^4J_{H\text{-}H}$ = 2.4 Hz, 1H, CH_{Ar}), 8.12 (d, $^3J_{H\text{-}H}$ = 8.9 Hz, 1H, CH_{Ar}), 7.81 (d, $^3J_{H\text{-}H}$ = 8.7 Hz, 2H, $2CH_{Ar}$), 7.75 (dd, $^4J_{H\text{-}H}$ = 2.4 Hz, $^3J_{H\text{-}H}$ = 8.9 Hz, 1H, CH_{Ar}), 7.68 (d, $^3J_{H\text{-}H}$ = 8.7 Hz, 2H, $2CH_{Ar}$), 7.67 (br s, 1H, NH), 7.32-7.22 (m, 5H, $5CH_{Ar}$), 3.23 (d, $^2J_{H\text{-}H}$ = 14.3 Hz, 1H, H-(CH_2), 3.08 (d, $^2J_{H\text{-}H}$ = 13.8 Hz, 1H, H-(CH_2)), 3.04 (d, $^2J_{H\text{-}H}$ = 13.8 Hz, 1H, H-(CH_2)), 2.92 (d, $^2J_{H\text{-}H}$ = 14.3 Hz, 1H, H-(CH_2), 1.54 (s, 3H, CH_3).

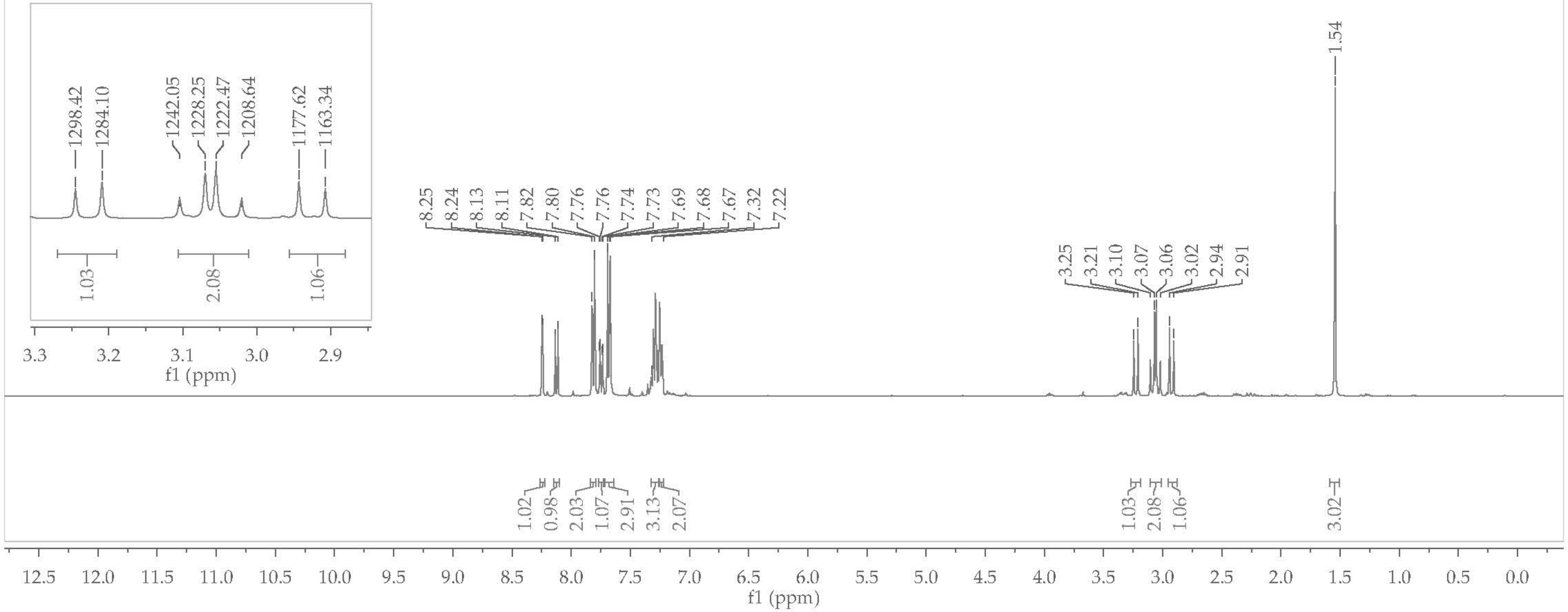

5-Benzyl-*N*-(5-bromopyridin-2-yl)-2-(4-cyanophenyl)-5-methyl-4,5-dihydrofuran-3-carboxamide (2) - $C_{25}H_{20}BrN_3O_2$

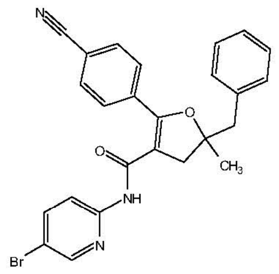

^{13}C NMR (100 MHz, $CDCl_3$): δ (ppm) 162.7 (C), 160.9 (C), 150.1 (C), 148.3 (CH_{Ar}), 141.1 (CH_{Ar}), 136.0 (C), 134.5 (C), 131.8 ($2CH_{Ar}$), 130.5 ($2CH_{Ar}$), 129.9 ($2CH_{Ar}$), 128.5 ($2CH_{Ar}$), 127.2 (CH_{Ar}), 118.5 (C), 115.5 (CH_{Ar}), 114.5 (C), 113.9 (C), 105.9 (C), 88.3 (C), 46.8 (CH_2), 42.1 (CH_2), 27.0 (CH_3).

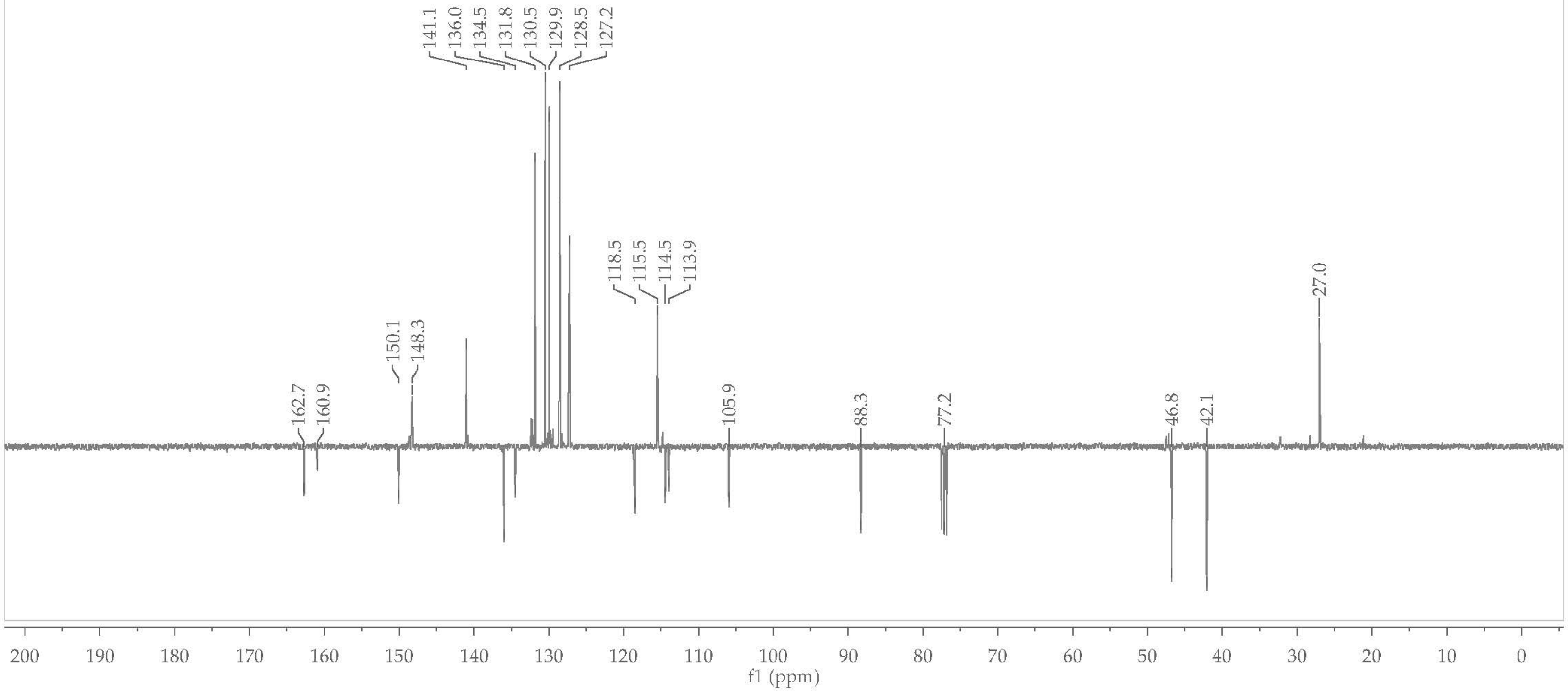

5-Benzyl-*N*-(5-chloropyridin-2-yl)-2-(4-cyanophenyl)-5-methyl-4,5-dihydrofuran-3-carboxamide (3) - $C_{25}H_{20}ClN_3O_2$

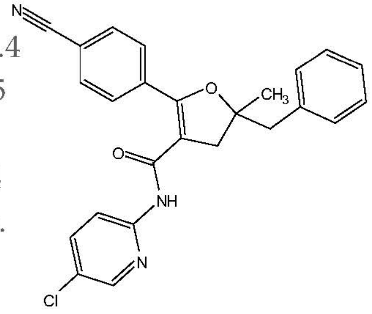

^{1}H NMR (400 MHz, $CDCl_3$): δ (ppm) 8.18 (d, $^3J_{H\text{-}H}$ = 8.9 Hz, 1H, CH_{Ar}), 8.14 (d, $^4J_{H\text{-}H}$ = 2.4 Hz, 1H, CH_{Ar}), 7.81 (d, $^3J_{H\text{-}H}$ = 8.5 Hz, 2H, $2CH_{Ar}$), 7.73 (br s, 1H, NH), 7.68 (d, $^3J_{H\text{-}H}$ = 8.5 Hz, 2H, $2CH_{Ar}$), 7.62 (dd, $^4J_{H\text{-}H}$ = 2.4 Hz, $^3J_{H\text{-}H}$= 8.9 Hz, 1H, CH_{Ar}), 7.33-7.23 (m, 5H, $5CH_{Ar}$), 3.23 (d, $^2J_{H\text{-}H}$ = 14.2 Hz, 1H, H-(CH_2), 3.08 (d, $^2J_{H\text{-}H}$ = 13.8 Hz, 1H, H-(CH_2)), 3.04 (d, $^2J_{H\text{-}H}$ = 13.8 Hz, 1H, H-(CH_2)), 2.93 (d, $^2J_{H\text{-}H}$ = 14.2 Hz, 1H, H-(CH_2), 1.54 (s, 3H, CH_3).

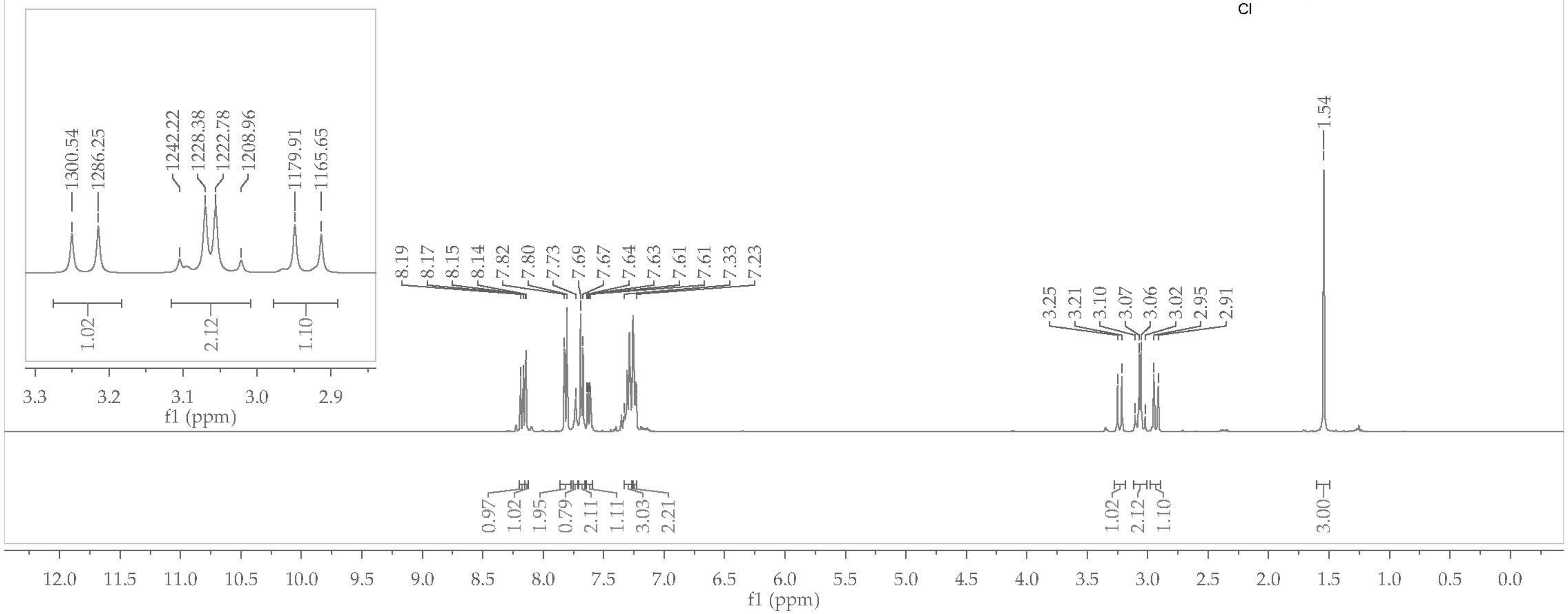

5-Benzyl-*N*-(5-chloropyridin-2-yl)-2-(4-cyanophenyl)-5-methyl-4,5-dihydrofuran-3-carboxamide (3) - $C_{25}H_{20}ClN_3O_2$

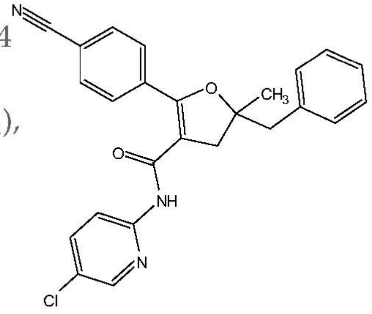

^{13}C NMR (100 MHz, $CDCl_3$): δ (ppm) 162.7 (C), 160.9 (C), 149.7 (C), 146.0 (CH_{Ar}), 138.4 (CH_{Ar}), 136.0 (C), 134.5 (C), 131.8 ($2CH_{Ar}$), 130.5 ($2CH_{Ar}$), 129.9 ($2CH_{Ar}$), 128.5 ($2CH_{Ar}$), 127.2 (CH_{Ar}), 126.7 (C), 118.5 (C), 115.0 (CH_{Ar}), 113.9 (C), 105.9 (C), 88.3 (C), 46.8 (CH_2), 42.1 (CH_2), 27.0 (CH_3).

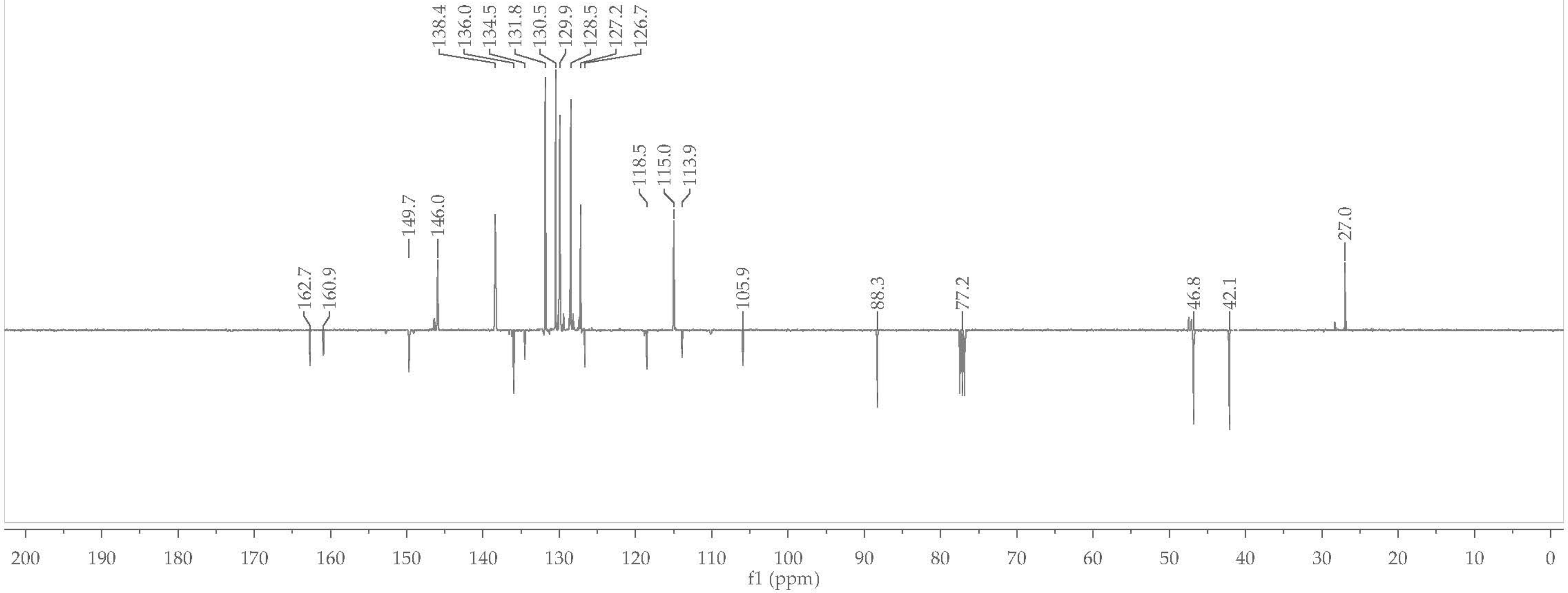

5-Benzyl-2-(4-cyanophenyl)-5-methyl-*N*-(5-methylpyridin-2-yl)-4,5-dihydrofuran-3-carboxamide (4) - $C_{26}H_{23}N_3O_2$

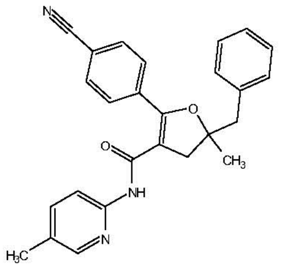

^{1}H NMR (400 MHz, $CDCl_3$): δ (ppm) 8.13 (d, $^3J_{H\text{-}H}$ = 8.6 Hz, 1H, CH_{Ar}), 8.01 (m, 2H, NH, CH_{Ar}), 7.82 (d, $^3J_{H\text{-}H}$ = 8.4 Hz, 2H, 2CH_{Ar}), 7.67 (d, $^3J_{H\text{-}H}$ = 8.4 Hz, 2H, 2CH_{Ar}), 7.53 (dd, $^4J_{H\text{-}H}$ = 2.0 Hz, $^3J_{H\text{-}H}$= 8.6 Hz, 1H, CH_{Ar}), 7.34-7.20 (m, 5H, 5CH_{Ar}), 3.27 (d, $^2J_{H\text{-}H}$ = 14.4 Hz, 1H, H-(CH_2), 3.08 (d, $^2J_{H\text{-}H}$ = 13.8 Hz, 1H, H-(CH_2)), 3.04 (d, $^2J_{H\text{-}H}$ = 13.8 Hz, 1H, H-(CH_2), 2.96 (d, $^2J_{H\text{-}H}$ = 14.4 Hz, 1H, H-(CH_2)), 2.28 (s, 3H, CH_3), 1.53 (s, 3H, CH_3).

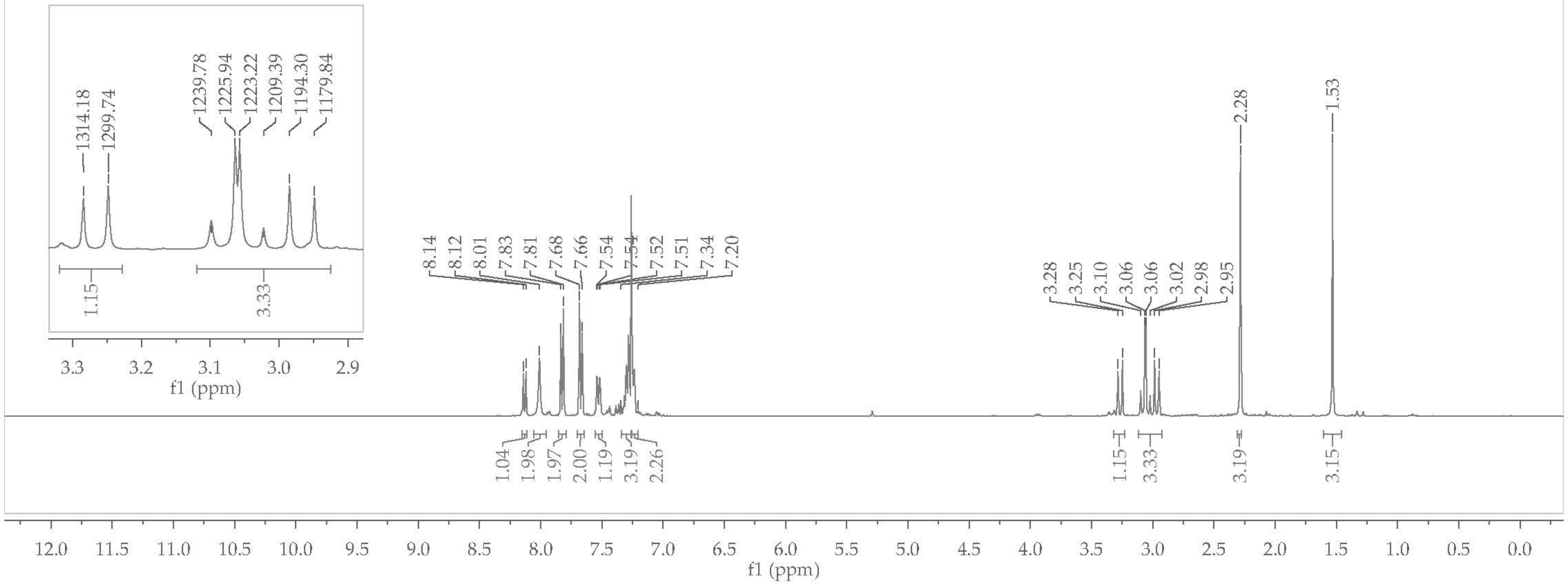

5-Benzyl-2-(4-cyanophenyl)-5-methyl-*N*-(5-methylpyridin-2-yl)-4,5-dihydrofuran-3-carboxamide (4) - $C_{26}H_{23}N_3O_2$

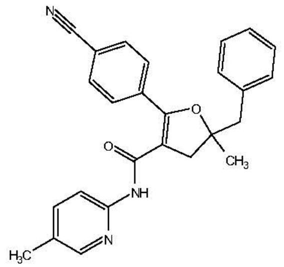

^{13}C NMR (100 MHz, $CDCl_3$): δ (ppm) 162.8 (C), 160.9 (C), 149.0 (C), 145.5 (CH_{Ar}), 140.5 (CH_{Ar}), 136.1 (C), 134.7 (C), 131.8 (2CH_{Ar}), 130.5 (2CH_{Ar}), 130.0 (2CH_{Ar}), 129.3 (C), 128.5 (2CH_{Ar}), 127.2 (CH_{Ar}), 118.6 (C), 114.4 (CH_{Ar}), 113.8 (C), 106.1 (C), 88.3 (C), 46.8 (CH_2), 42.3 (CH_2), 27.0 (CH_3), 17.9 (CH_3).

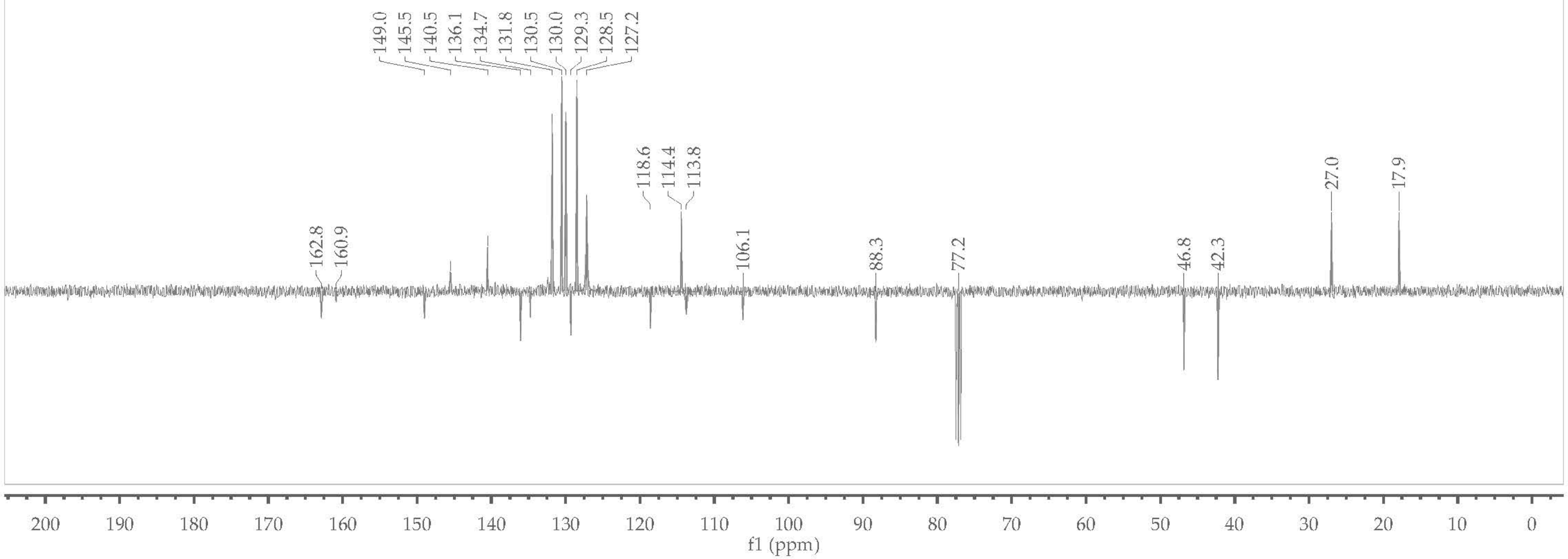

5-benzyl-2-(4-cyanophenyl)-5-methyl-N-(pyridin-2-yl)-4,5-dihydrofuran-3-carboxamide (5) - $C_{25}H_{21}N_3O_2$

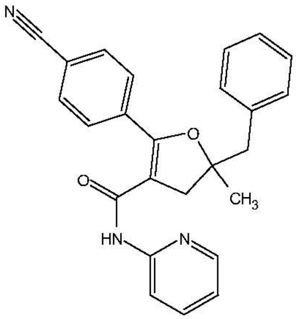

^{1}H NMR (400 MHz, $CDCl_3$): δ (ppm) 8.24-8.17 (m, 2H, $2CH_{Ar}$), 7.89 (br s, 1H, NH), 7.83 (d, $^3J_{H\text{-}H}$ = 8.6 Hz, 2H, $2CH_{Ar}$), 7.70-7.67 (m, 3H, $3CH_{Ar}$), 7.33-7.23 (m, 5H, $5CH_{Ar}$), 7.05-7.01 (m, 1H, CH_{Ar}), 3.26 (d, $^2J_{H\text{-}H}$ = 14.4 Hz, 1H, H-(CH_2)), 3.09 (d, $^2J_{H\text{-}H}$ = 13.8 Hz, 1H, H-(CH_2)), 3.05 (d, $^2J_{H\text{-}H}$ = 13.8 Hz, 1H, H-(CH_2)), 2.96 (d, $^2J_{H\text{-}H}$ = 14.4 Hz, 1H, H-(CH_2)), 1.54 (s, 3H, CH_3).

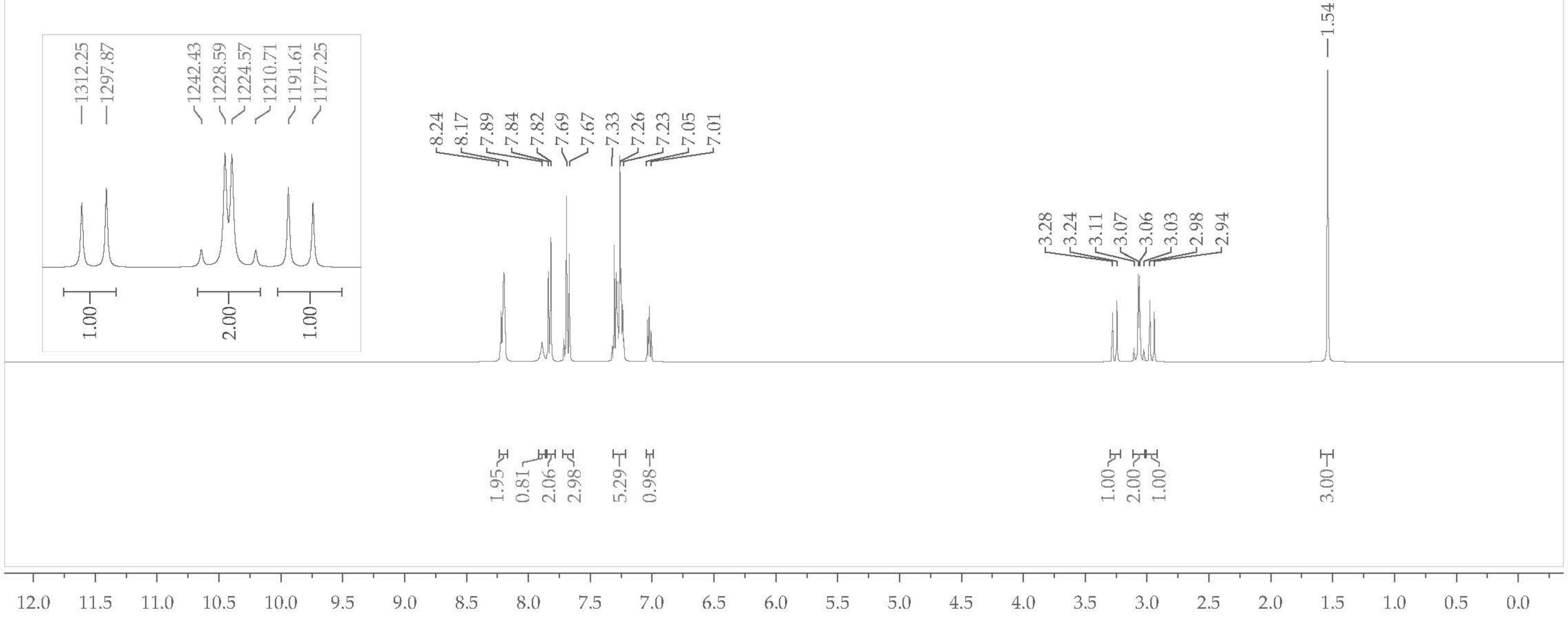

5-benzyl-2-(4-cyanophenyl)-5-methyl-N-(pyridin-2-yl)-4,5-dihydrofuran-3-carboxamide (5) - $C_{25}H_{21}N_3O_2$

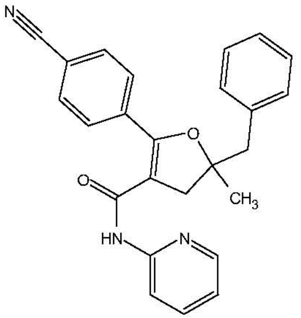

^{13}C NMR (100 MHz, $CDCl_3$): δ (ppm) 162.9 (C), 160.7 (C), 151.3 (C), 146.9 (CH_{Ar}), 139.1 (CH_{Ar}), 136.1 (C), 134.7 (C), 131.8 ($2CH_{Ar}$), 130.5 ($2CH_{Ar}$), 130.0 ($2CH_{Ar}$), 128.5 ($2CH_{Ar}$), 127.2 (CH_{Ar}), 119.7 (CH_{Ar}), 118.6 (C), 114.5 (CH_{Ar}), 113.8 (C), 106.2 (C), 88.2 (C), 46.8 (CH_2), 42.3 (CH_2), 27.0 (CH_3).

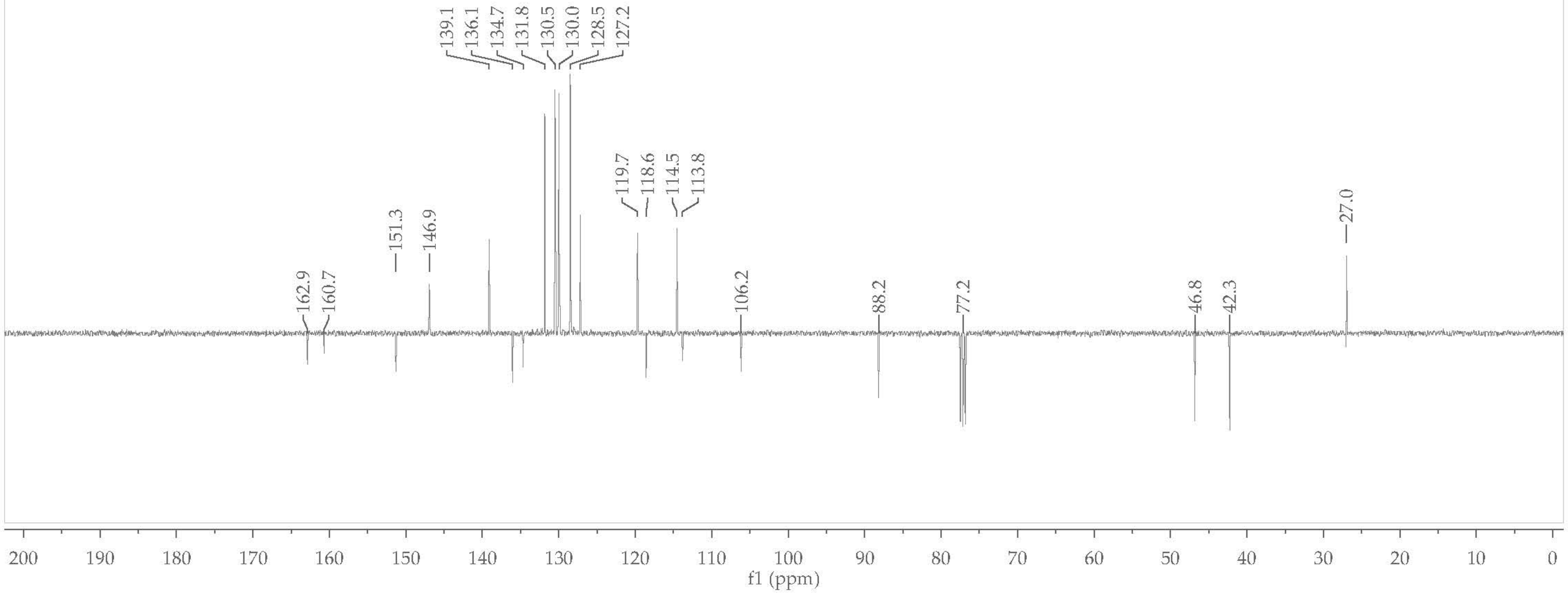

5-benzyl-2-(4-cyanophenyl)-5-methyl-*N*-(pyridin-4-yl)-4,5-dihydrofuran-3-carboxamide (6) - $C_{25}H_{21}N_3O_2$

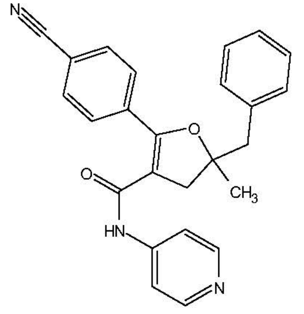

^{1}H NMR (400 MHz, $CDCl_3$): δ (ppm) 8.35 (d, $^3J_{H\text{-}H}$ = 6.5 Hz, 2H, $2CH_{Ar}$), 7.78 (d, $^3J_{H\text{-}H}$ = 8.6 Hz, 2H, $2CH_{Ar}$), 7.68 (d, $^3J_{H\text{-}H}$ = 8.6 Hz, 2H, $2CH_{Ar}$), 7.44 (d, $^3J_{H\text{-}H}$ = 6.5 Hz, 2H, $2CH_{Ar}$), 7.39 (br s, 1H, NH), 7.32-7.23 (m, 5H, $5CH_{Ar}$), 3.24 (d, $^2J_{H\text{-}H}$ = 14.5 Hz, 1H, H-(CH_2), 3.07 (d, $^2J_{H\text{-}H}$ = 13.9 Hz, 1H, H-(CH_2), 3.04 (d, $^2J_{H\text{-}H}$ = 13.9 Hz, 1H, H-(CH_2), 2.98 (d, $^2J_{H\text{-}H}$ = 14.5 Hz, 1H, H-(CH_2)), 1.55 (s, 3H, CH_3).

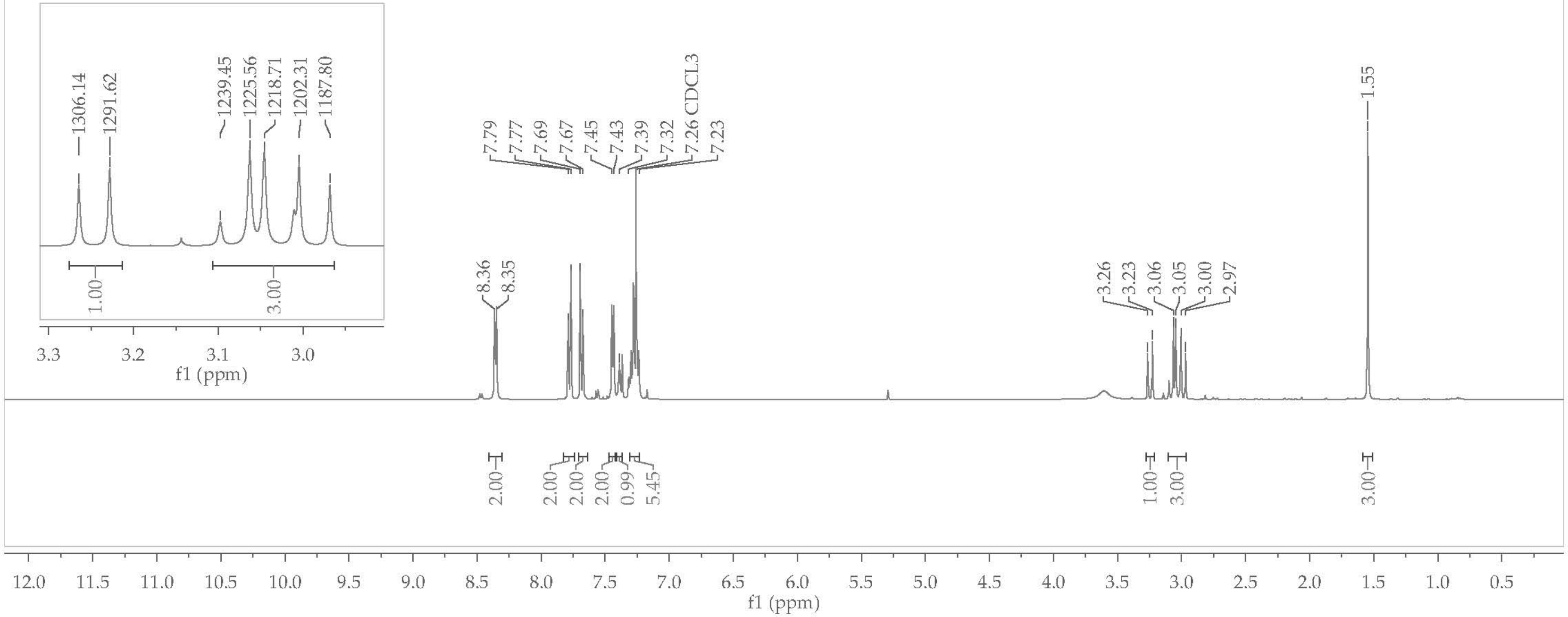

5-benzyl-2-(4-cyanophenyl)-5-methyl-*N*-(pyridin-4-yl)-4,5-dihydrofuran-3-carboxamide (6) - $C_{25}H_{21}N_3O_2$

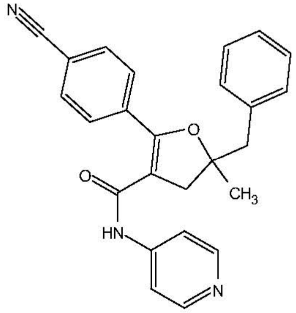

^{13}C NMR (100 MHz, $CDCl_3$): δ (ppm) 163.2 (C), 159.9 (C), 150.5 ($2CH_{Ar}$), 145.1 (C), 136.1 (C), 134.4 (C), 132.0 ($2CH_{Ar}$), 130.5 ($2CH_{Ar}$), 129.8 ($2CH_{Ar}$), 128.5 ($2CH_{Ar}$), 127.2 (CH_{Ar}), 118.4 (C), 114.1 (C), 113.5 ($2CH_{Ar}$), 106.5 (C), 88.3 (C), 46.9 (CH_2), 42.3 (CH_2), 27.1 (CH_3).

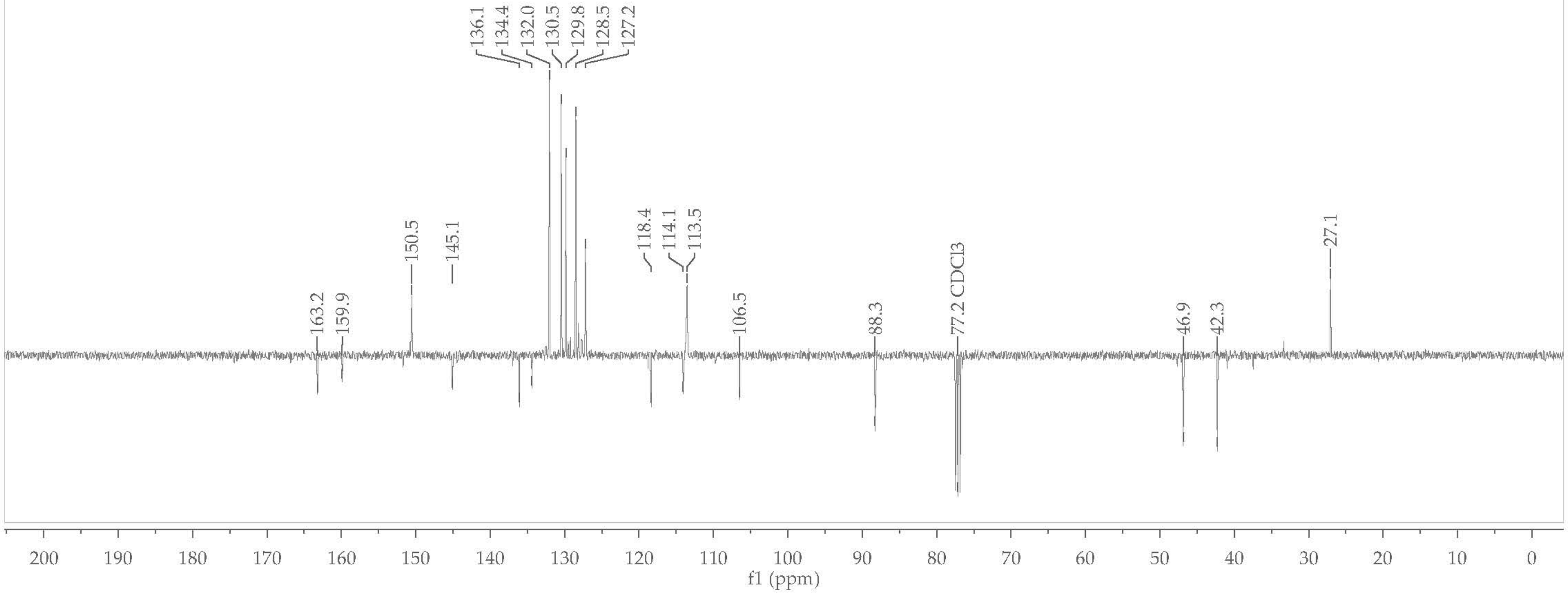

5-benzyl-2-(4-cyanophenyl)-5-methyl-*N*-phenyl-4,5-dihydrofuran-3-carboxamide (7) - $C_{26}H_{22}N_2O_2$

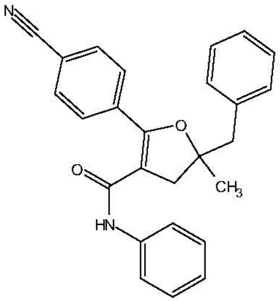

^{1}H NMR (400 MHz, $CDCl_3$): δ (ppm) 7.81 (d, $^3J_{H\text{-}H}$ = 8.4 Hz, 2H, 2CH_{Ar}), 7.67 (d, $^3J_{H\text{-}H}$ = 8.4 Hz, 2H, 2CH_{Ar}), 7.52-7.43 (m, 1H, CH_{Ar}), 7.34-7.27 (m, 8H, 8CH_{Ar}), 7.08 (t, $^3J_{H\text{-}H}$ = 7.2 Hz, 1H, CH_{Ar}), 6.70 (br s, 1H, NH), 3.19 (d, $^2J_{H\text{-}H}$ = 14.7 Hz, 1H, H-(CH_2), 3.08 (d, $^2J_{H\text{-}H}$ = 13.8 Hz, 1H, H-(CH_2), 3.03 (d, $^2J_{H\text{-}H}$ = 13.8 Hz, 1H, H-(CH_2), 2.95 (d, $^2J_{H\text{-}H}$ = 14.7 Hz, 1H, H-(CH_2), 1.55 (s, 3H, CH_3).

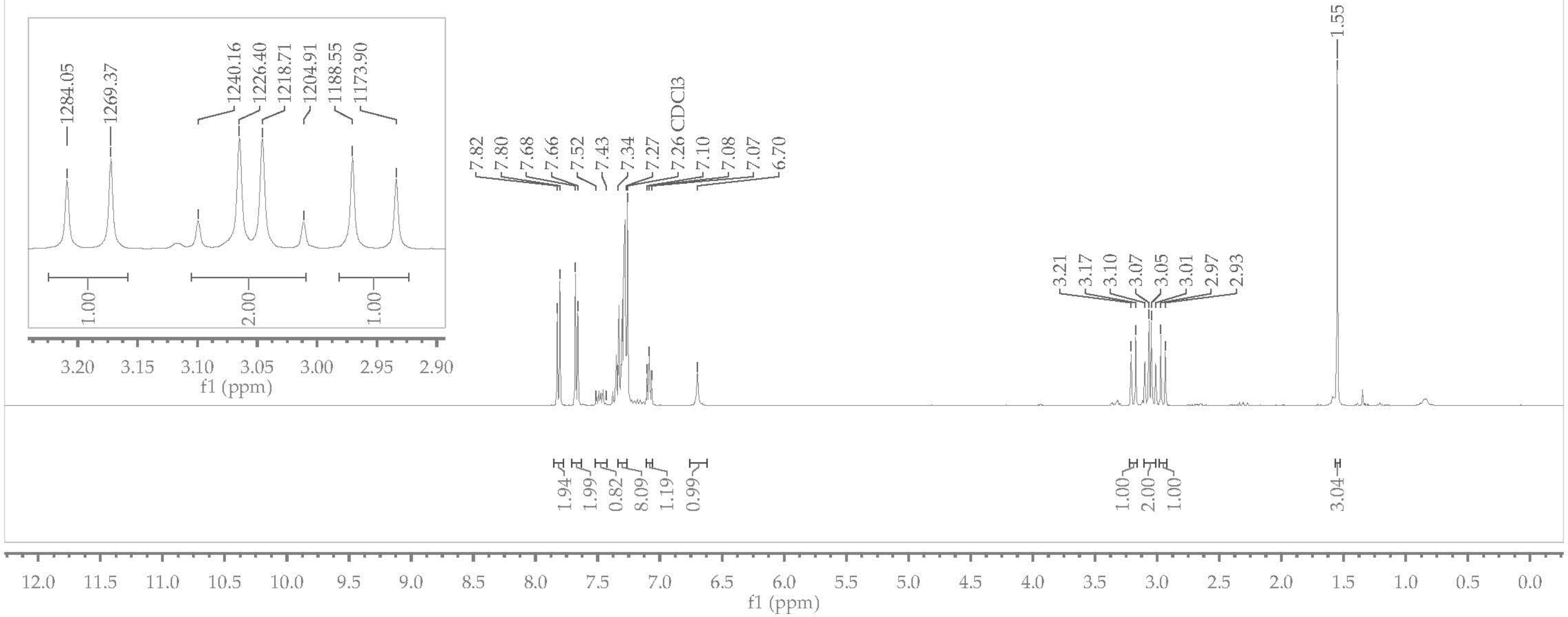

5-benzyl-2-(4-cyanophenyl)-5-methyl-*N*-phenyl-4,5-dihydrofuran-3-carboxamide (7) - $C_{26}H_{22}N_2O_2$

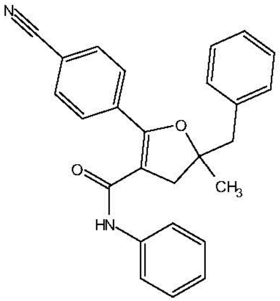

^{13}C NMR (100 MHz, $CDCl_3$): δ (ppm) 169.2 (C), 157.9 (C), 137.8 (C), 136.4 (C), 134.6 (C), 132.0 ($2CH_{Ar}$), 130.5 ($2CH_{Ar}$), 129.7 ($2CH_{Ar}$), 129.2 ($2CH_{Ar}$), 128.5 ($2CH_{Ar}$), 127.1 (CH_{Ar}), 124.5 (CH_{Ar}), 120.0 ($2CH_{Ar}$), 118.5 (C), 113.7 (C), 107.3 (C), 87.6 (C), 47.0 (CH_2), 42.8 (CH_2), 27.1 (CH_3).

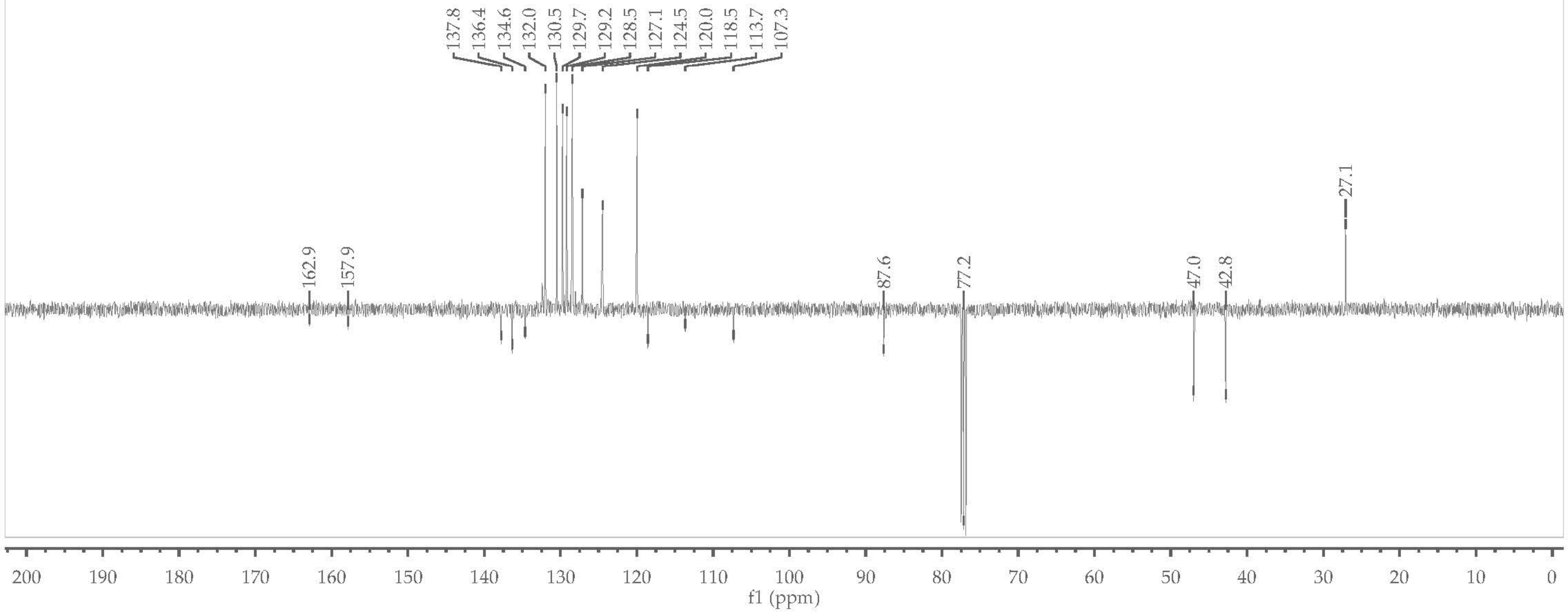

5-Benzyl-2-(4-cyanophenyl)-*N*-(4-fluorophenyl)-5-methyl-4,5-dihydrofuran-3-carboxamide (8) - $C_{26}H_{21}FN_2O_2$

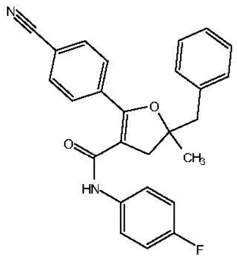

^{1}H NMR (400 MHz, $CDCl_3$): δ (ppm) 7.81 (d, $^3J_{H\text{-}H}$ = 8.6 Hz, 2H, 2CH_{Ar}), 7.67 (d, $^3J_{H\text{-}H}$ = 8.6 Hz, 2H, 2CH_{Ar}), 7.34-7.25 (m, 7H, 7CH_{Ar}), 7.02-6.95 (m, 2H, 2CH_{Ar}), 6.66 (br s, 1H, NH), 3.18 (d, $^2J_{H\text{-}H}$ = 14.5 Hz, 1H, H-(CH_2), 3.08 (d, $^2J_{H\text{-}H}$ = 13.7 Hz, 1H, H-(CH_2), 3.03 (d, $^2J_{H\text{-}H}$ = 13.7 Hz, 1H, H-(CH_2), 2.93 (d, $^2J_{H\text{-}H}$ = 14.5 Hz, 1H, H-(CH_2), 1.55 (s, 3H, CH_3).

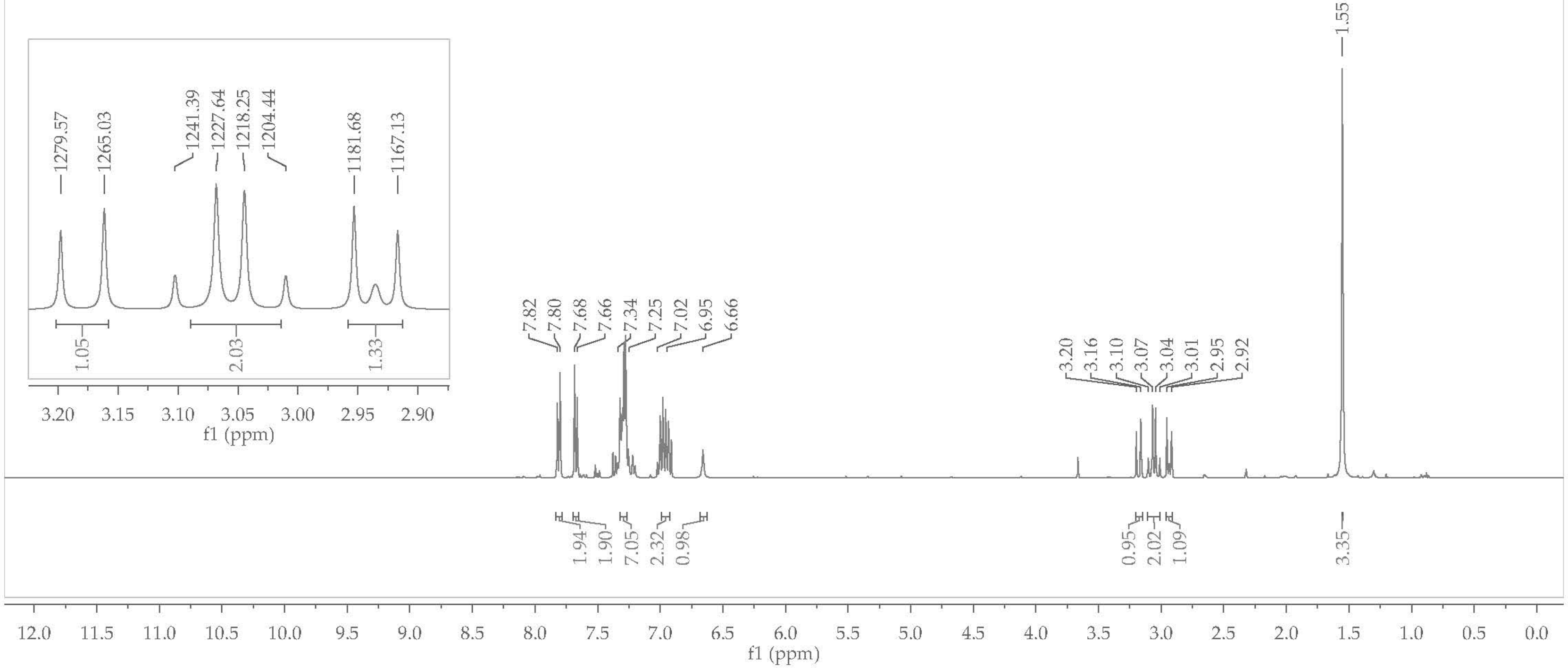

5-Benzyl-2-(4-cyanophenyl)-*N*-(4-fluorophenyl)-5-methyl-4,5-dihydrofuran-3-carboxamide (8) - $C_{26}H_{21}FN_2O_2$

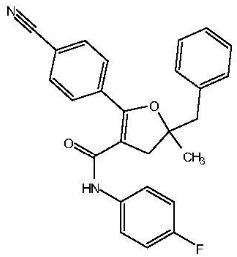

^{13}C NMR (100 MHz, $CDCl_3$): δ (ppm) 162.9 (C), 159.5 (d, $J_{C\text{-}F}$ = 246.3 Hz, C), 158.3 (C), 136.8 (C), 136.3 (C), 133.7 (d, $J_{C\text{-}F}$ = 3.1 Hz, C), 130.5 (2CH_{Ar}), 129.8 (2CH_{Ar}), 128.5 (2CH_{Ar}), 127.3 (2CH_{Ar}), 127.2 (CH_{Ar}), 121.1 (d, $J_{C\text{-}F}$ = 8.6 Hz, 2CH_{Ar}), 118.5 (C), 115.9 (d, $J_{C\text{-}F}$ = 21.6 Hz, 2CH_{Ar}), 113.7 (C), 106.9 (C), 87.7 (C), 47.0 (CH_2), 42.7 (CH_2), 27.1 (CH_3).

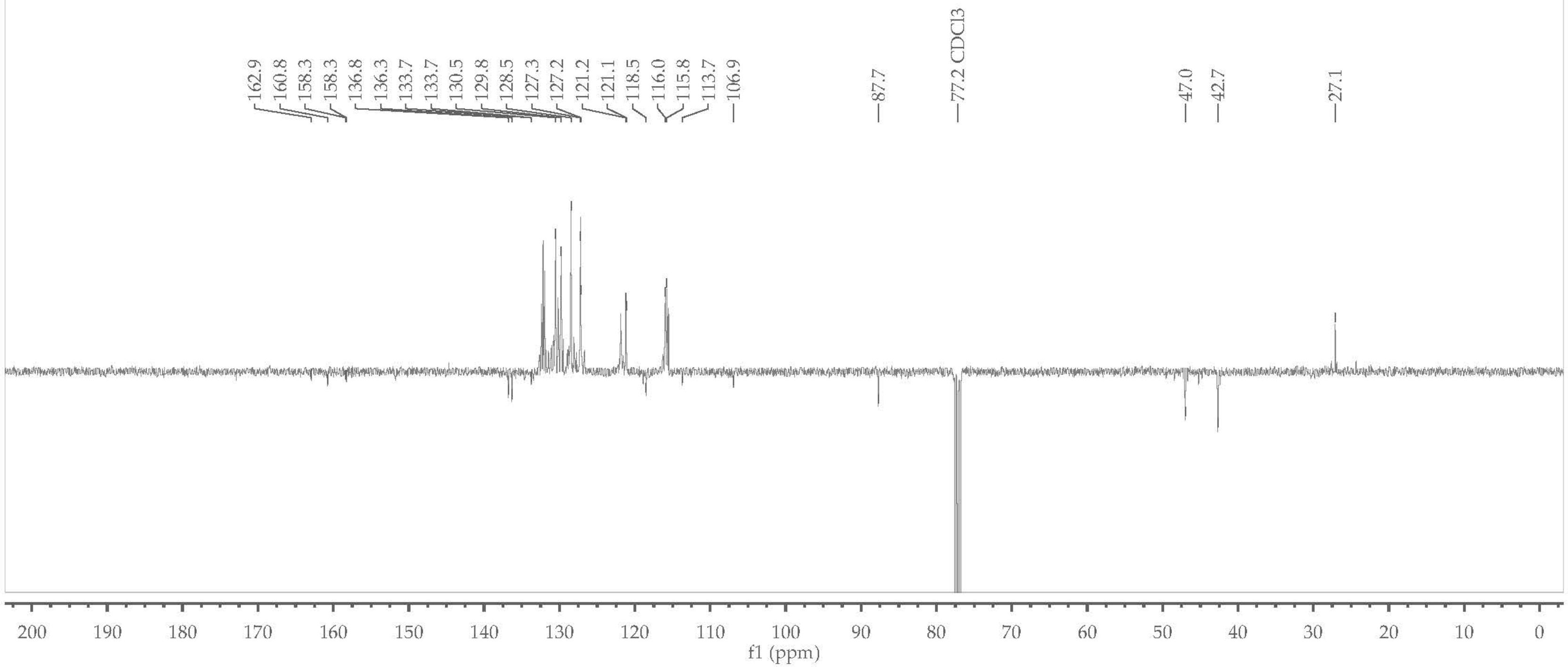

5-Benzyl-2-(4-cyanophenyl)-5-methyl-*N*-(4-(trifluoromethyl)benzyl)-4,5-dihydrofuran-3-carboxamide (9) - $C_{28}H_{23}F_3N_2O_2$

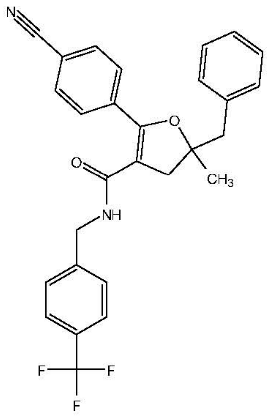

^{1}H NMR (400 MHz, $CDCl_3$): δ (ppm) 7.81 (d, $^3J_{H\text{-}H}$ = 8.4 Hz, 2H, $2CH_{Ar}$), 7.62 (d, $^3J_{H\text{-}H}$ = 8.4 Hz, 2H, $2CH_{Ar}$), 7.58 (d, $^3J_{H\text{-}H}$ = 8.1 Hz, 2H, $2CH_{Ar}$), 7.32 (d, $^3J_{H\text{-}H}$ = 8.1 Hz, 2H, $2CH_{Ar}$), 7.27-7.22 (m, 5H, $5CH_{Ar}$), 5.44 (br s, 1H, NH), 4.50-4.42 (m, 2H, CH_2), 3.07 (d, $^2J_{H\text{-}H}$ = 14.5 Hz, 1H, H-(CH_2)), 3.04 (d, $^2J_{H\text{-}H}$ = 13.8 Hz, 1H, H-(CH_2)), 2.99 (d, $^2J_{H\text{-}H}$ = 13.8 Hz, 1H, H-(CH_2)), 2.82 (d, $^2J_{H\text{-}H}$ = 14.5 Hz, 1H, H-(CH_2)), 1.51 (s, 3H, CH_3).

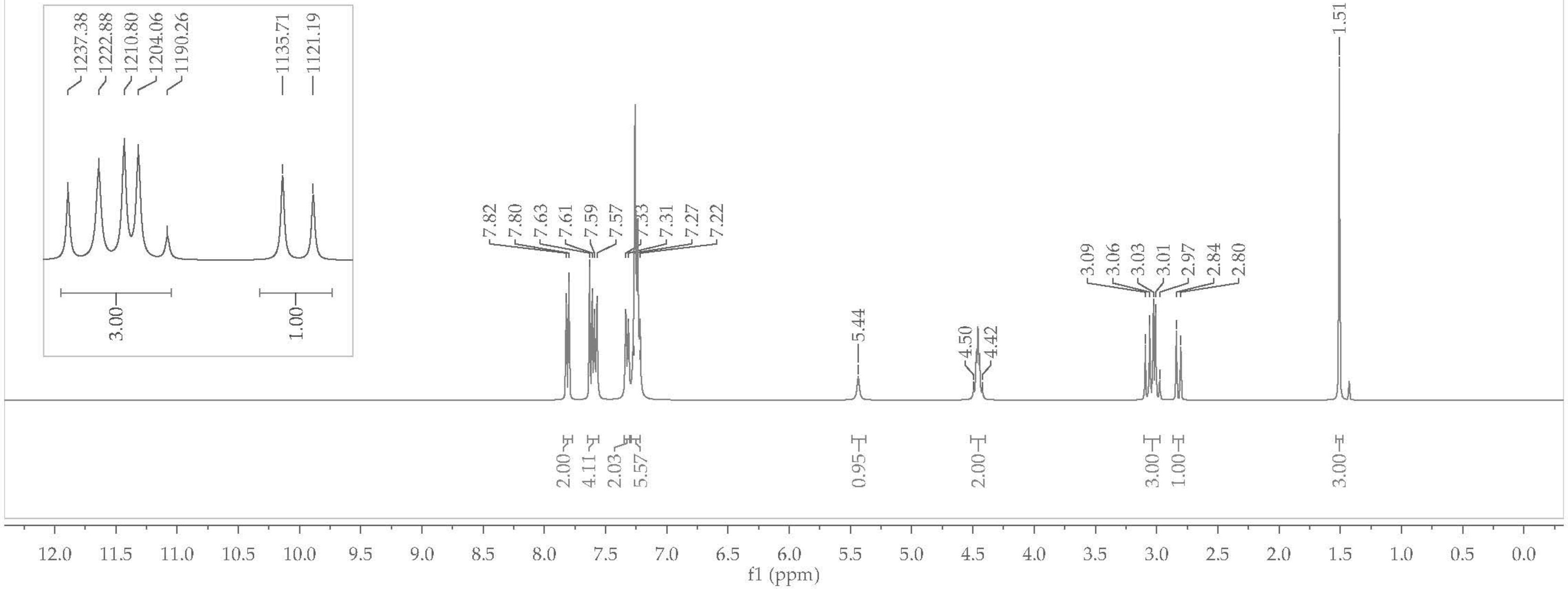

5-Benzyl-2-(4-cyanophenyl)-5-methyl-*N*-(4-(trifluoromethyl)benzyl)-4,5-dihydrofuran-3-carboxamide (9) - $C_{28}H_{23}F_3N_2O_2$

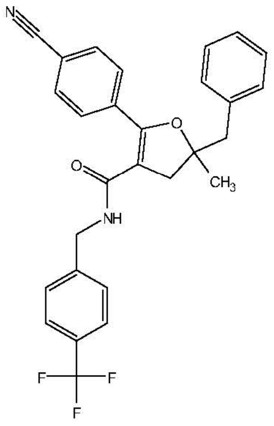

^{13}C NMR (100 MHz, $CDCl_3$): δ (ppm) 164.7 (C), 158.0 (C), 142.4 (C), 136.3 (C), 134.7 (C), 131.7 (2CH_{Ar}), 130.5 (2CH_{Ar}), 130.2 (q, $^2J_{C\text{-}F}$ = 34.6 Hz, C), 129.8 (2CH_{Ar}), 128.4 (2CH_{Ar}), 128.1 (2CH_{Ar}), 127.1 (CH_{Ar}), 125.8 (q, $^3J_{C\text{-}F}$ = 3.7 Hz, 2CH_{Ar}), 124.2 (q, $^1J_{C\text{-}F}$ = 271.8 Hz, CF_3), 118.6 (C), 113.5 (C), 106.1 (C), 87.4 (C), 47.0 (CH_2), 43.0 (CH_2), 42.7 (CH_2), 27.1 (CH_3).

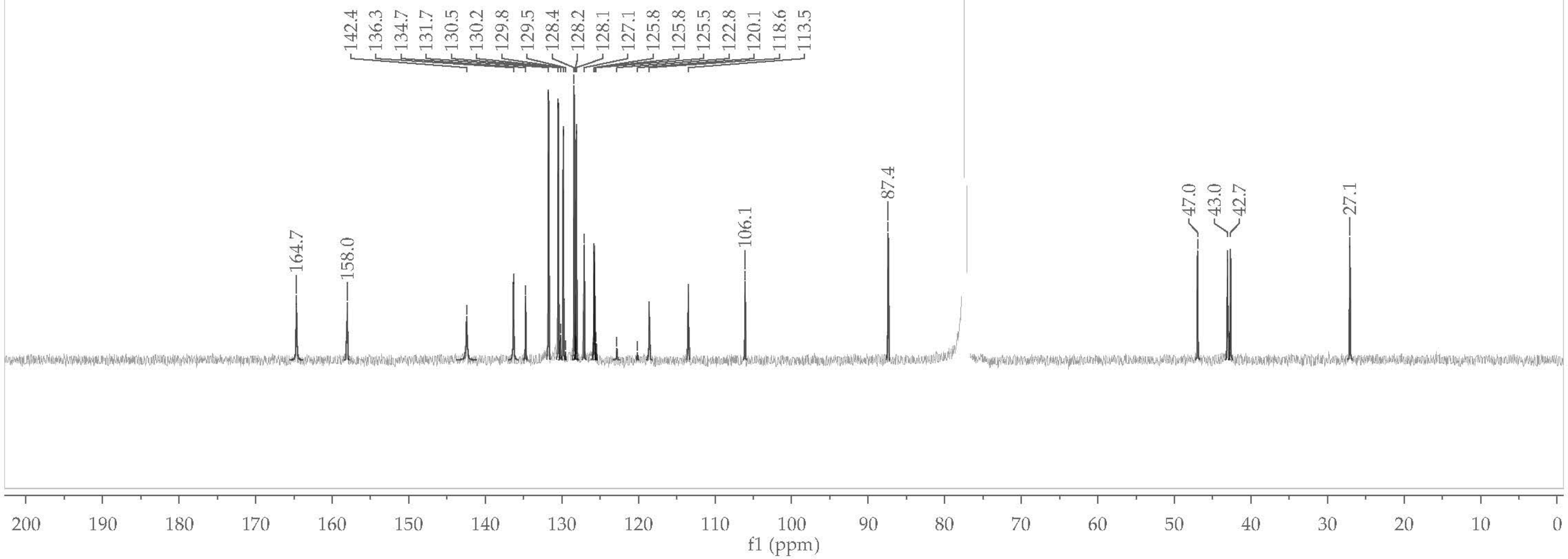

5-benzyl-2-(4-cyanophenyl)-5-methyl-N-(pyridazin-3-yl)-4,5-dihydrofuran-3-carboxamide (10) - $C_{24}H_{20}N_4O_2$

^{1}H NMR (400 MHz, $CDCl_3$): δ (ppm) 8.84 (d, $^{3}J_{H\text{-}H}$ = 12.1 Hz, 1H, CH_{Ar}), 8.68 (br s, 1H, NH), 8.50 (d, $^{3}J_{H\text{-}H}$ = 8.7 Hz, 1H, CH_{Ar}), 7.81 (d, $^{3}J_{H\text{-}H}$ = 8.4 Hz, 2H, $2CH_{Ar}$), 7.68 (d, $^{3}J_{H\text{-}H}$ = 8.4 Hz, 2H, $2CH_{Ar}$), 7.61 (dd, $^{3}J_{H\text{-}H}$ = 12.1 Hz, $^{3}J_{H\text{-}H}$ = 8.7 Hz, 1H, CH_{Ar}), 7.35-7.20 (m, 5H, $5CH_{Ar}$), 3.32 (d, $^{2}J_{H\text{-}H}$ = 14.3 Hz, 1H, H-(CH_2)), 3.10 (d, $^{2}J_{H\text{-}H}$ = 13.8 Hz, 1H, H-(CH_2)), 3.06 (d, $^{2}J_{H\text{-}H}$ = 13.8 Hz, 1H, H-(CH_2)), 3.02 (d, $^{2}J_{H\text{-}H}$ = 14.3 Hz, 1H, H-(CH_2)), 1.56 (s, 3H, CH_3).

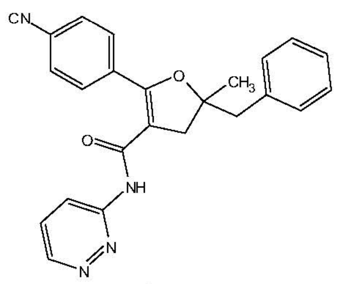

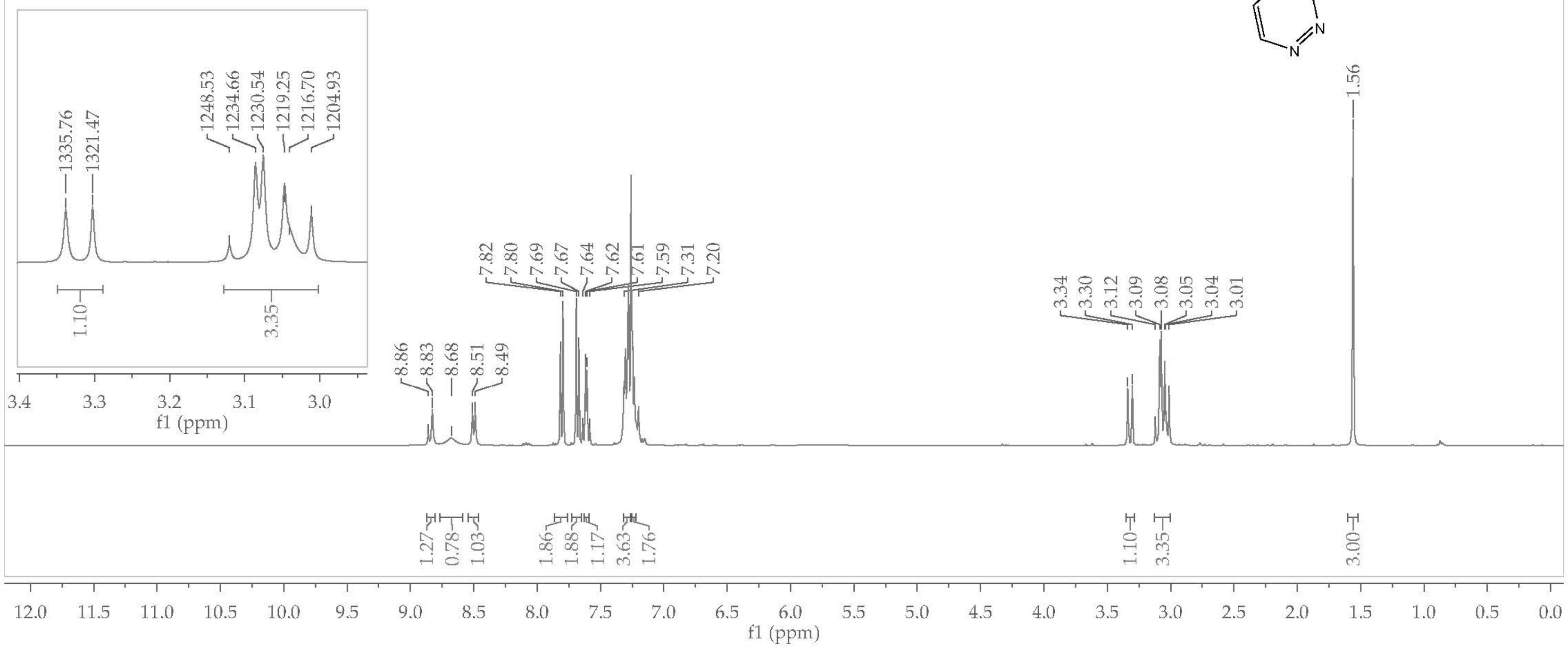

5-benzyl-2-(4-cyanophenyl)-5-methyl-N-(pyridazin-3-yl)-4,5-dihydrofuran-3-carboxamide (10) - $C_{24}H_{20}N_4O_2$

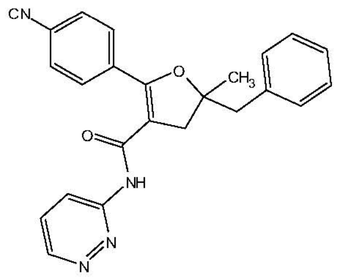

^{13}C NMR (100 MHz, $CDCl_3$): δ (ppm) 163.6 (C), 162.1 (C), 155.3 (C), 152.0 (C), 148.2 (CH_{Ar}), 135.9 (C), 134.5 (C), 131.8 ($2CH_{Ar}$), 130.5 ($2CH_{Ar}$), 130.0 ($2CH_{Ar}$), 128.5 ($2CH_{Ar}$), 128.1 (CH_{Ar}), 127.2 (CH_{Ar}), 119.7 (CH_{Ar}), 118.4 (C), 105.7 (C), 88.7 (C), 46.8 (CH_2), 41.9 (CH_2), 27.0 (CH_3).

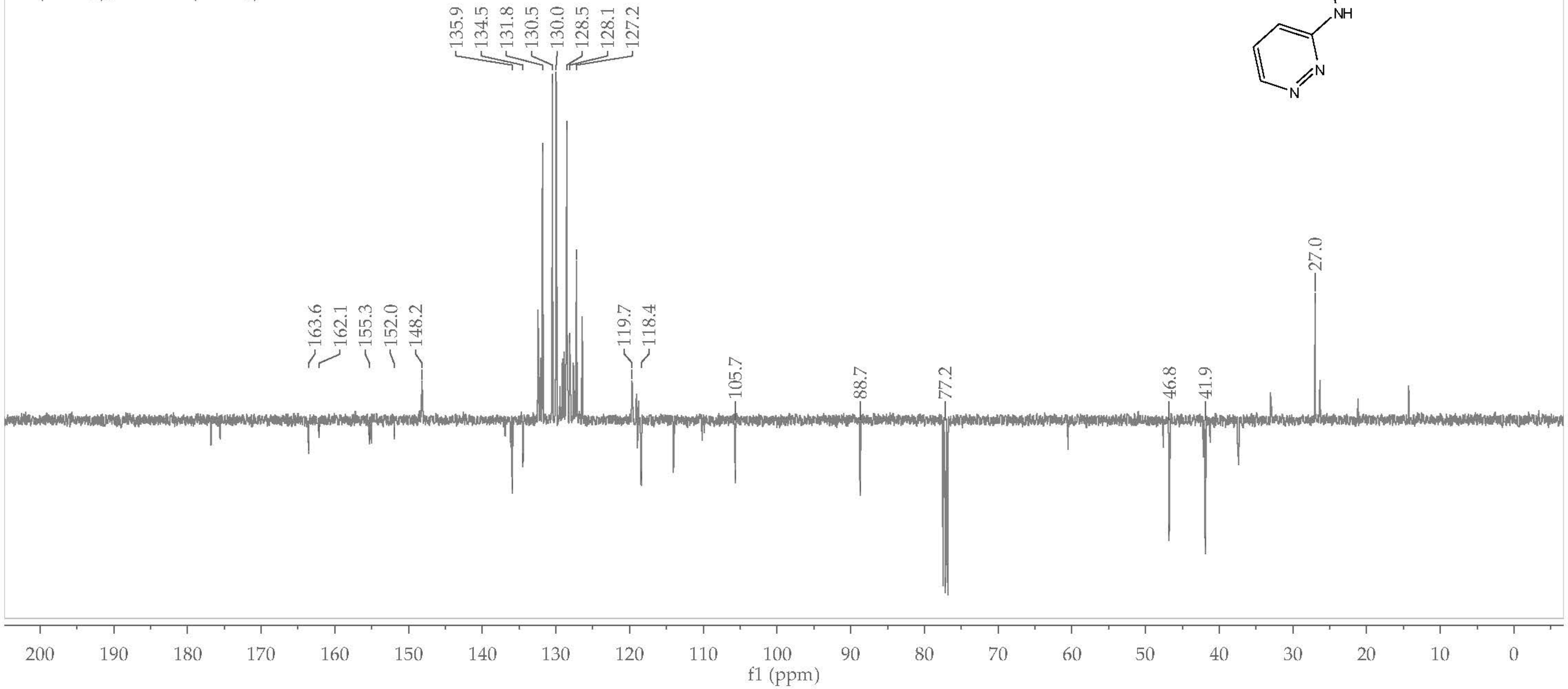

5-Benzyl-*N*-(6-chloropyridazin-3-yl)-2-(4-cyanophenyl)-5-methyl-4,5-dihydrofuran-3-carboxamide (11) - $C_{24}H_{19}ClN_4O_2$

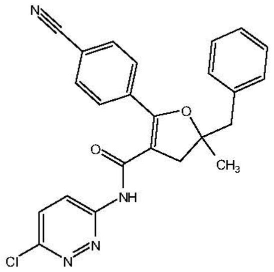

^{1}H NMR (400 MHz, $CDCl_3$): δ (ppm) 8.53 (d, $^3J_{H\text{-}H}$ = 9.5 Hz, 1H, CH_{Ar}), 7.80 (d, $^3J_{H\text{-}H}$ = 8.6 Hz, 2H, $2CH_{Ar}$), 7.69 (d, $^3J_{H\text{-}H}$ = 8.6 Hz, 2H, $2CH_{Ar}$), 7.48 (d, $^3J_{H\text{-}H}$ = 9.5 Hz, 1H, CH_{Ar}), 7.35-7.20 (m, 5H, $5CH_{Ar}$), 3.31 (d, $^2J_{\text{H-H}}$ = 14.3 Hz, 1H, H-(CH_2), 3.11 (d, $^2J_{\text{H-H}}$ = 13.9 Hz, 1H, H-(CH_2), 3.05 (d, $^2J_{\text{H-H}}$ = 13.9 Hz, 1H, H-(CH_2), 3.01 (d, $^2J_{\text{H-H}}$ = 14.3 Hz, 1H, H-(CH_2), 1.58 (s, 3H, CH_3). NH not obsrved.

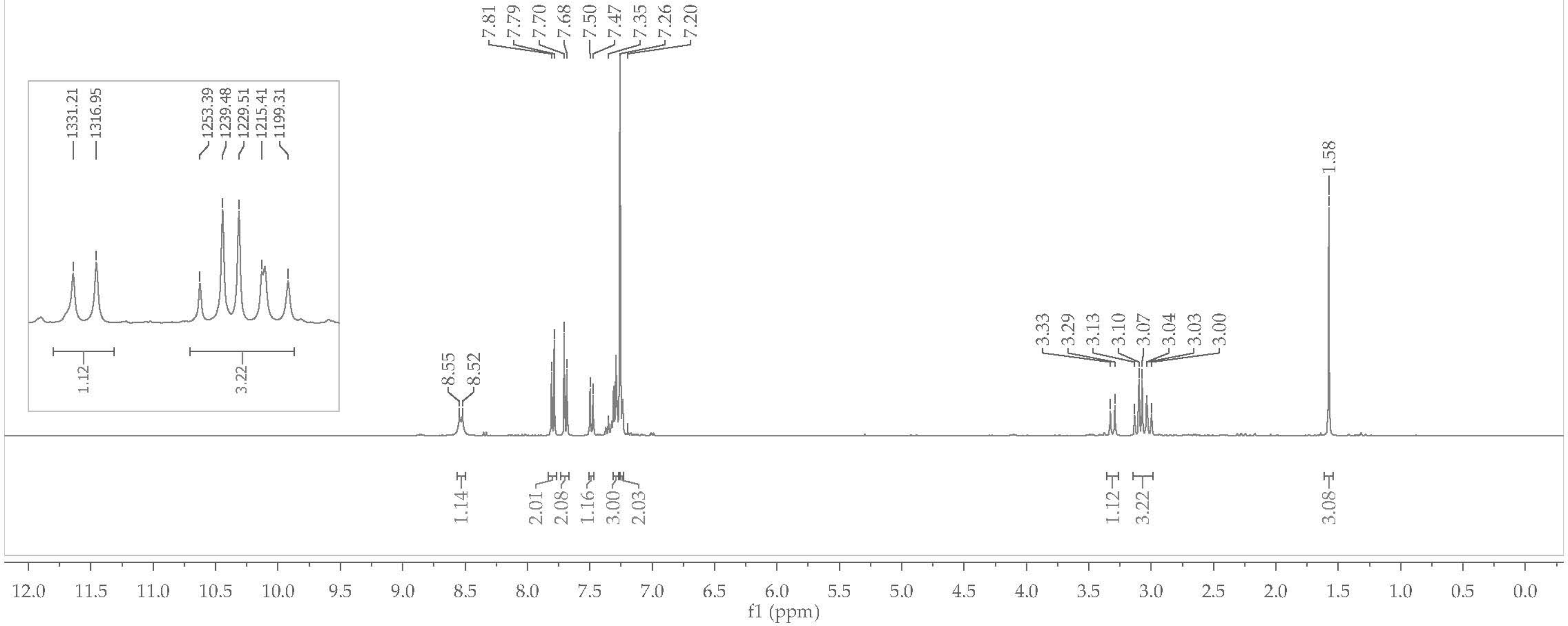

5-Benzyl-*N*-(6-chloropyridazin-3-yl)-2-(4-cyanophenyl)-5-methyl-4,5-dihydrofuran-3-carboxamide (11) - $C_{24}H_{19}ClN_4O_2$

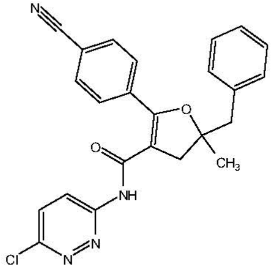

^{13}C NMR (100 MHz, $CDCl_3$): δ (ppm) 163.3 (C), 154.4 (C), 152.0 (C), 135.9 (2C), 134.4 (C), 131.9 ($2CH_{Ar}$), 130.6 (CH_{Ar}), 130.5 ($2CH_{Ar}$), 130.0 ($2CH_{Ar}$), 128.5 ($2CH_{Ar}$), 127.3 (CH_{Ar}), 122.0 (CH_{Ar}), 118.4 (C), 114.2 (C), 105.3 (C), 89.0 (C), 46.8 (CH_2), 41.7 (CH_2), 27.1 (CH_3).

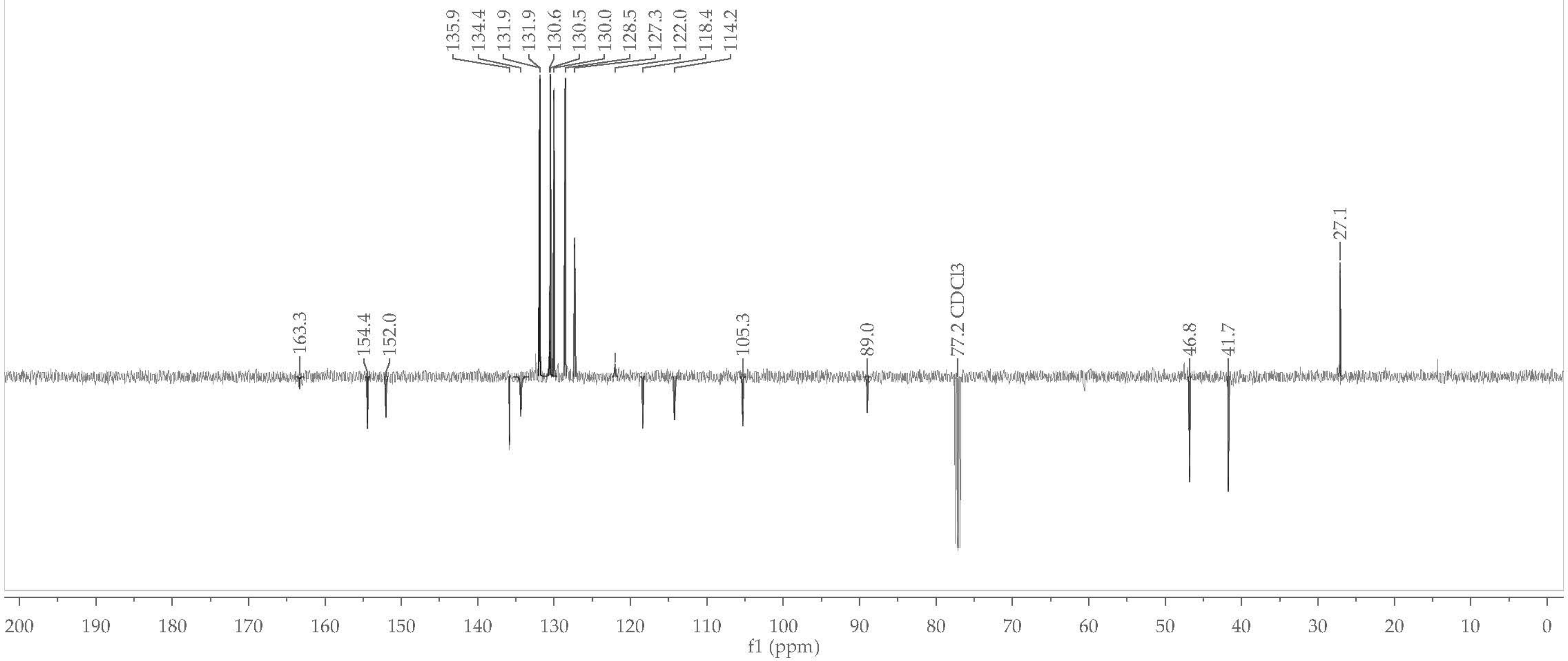

5-Benzyl-2-(4-cyanophenyl)-5-methyl-*N*-(pyrazin-2-yl)-4,5-dihydrofuran-3-carboxamide (12) - $C_{24}H_{20}N_4O_2$

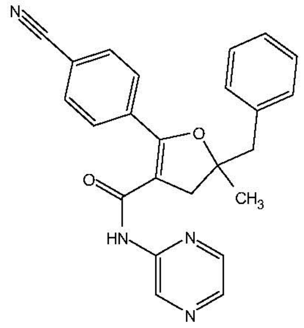

^{1}H NMR (400 MHz, $CDCl_3$): δ (ppm) 9.48 (s, 1H, NH), 8.28 (d, $^3J_{H\text{-}H}$ = 2.4 Hz, 1H, CH_{Ar}), 8.17 (d, $^3J_{H\text{-}H}$ = 2.4 Hz, 1H, CH_{Ar}), 7.82 (d, $^3J_{H\text{-}H}$ = 8.5 Hz, 2H, $2CH_{Ar}$), 7.70 (d, $^3J_{H\text{-}H}$ = 8.5 Hz, 2H, $2CH_{Ar}$), 7.49 (br s, 1H, CH_{Ar}), 7.34-7.20 (m, 5H, $5CH_{Ar}$), 3.24 (d, $^2J_{H\text{-}H}$ = 14.3 Hz, 1H, H-(CH_2)), 3.10 (d, $^2J_{H\text{-}H}$ = 13.8 Hz, 1H, H-(CH_2)), 3.04 (d, $^2J_{H\text{-}H}$ = 13.8 Hz, 1H, H-(CH_2)), 2.95 (d, $^2J_{H\text{-}H}$ = 14.3 Hz, 1H, H-(CH_2)), 1.56 (s, 3H, CH_3).

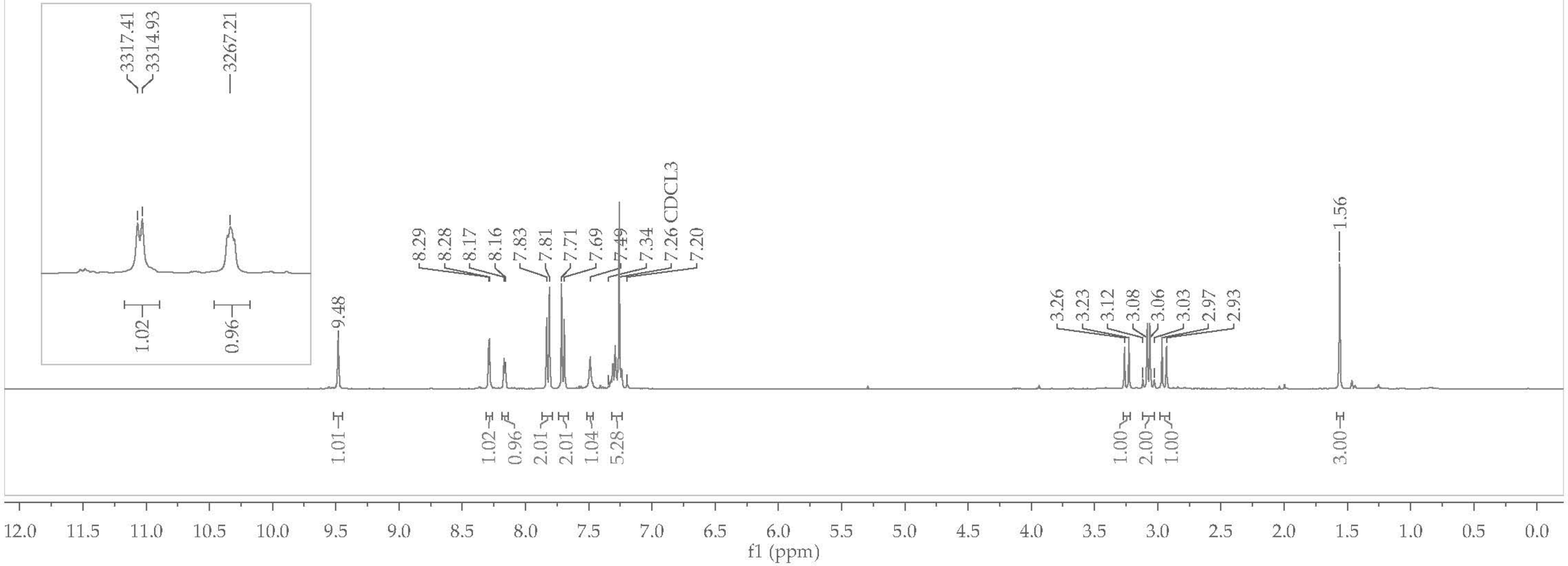

5-Benzyl-2-(4-cyanophenyl)-5-methyl-*N*-(pyrazin-2-yl)-4,5-dihydrofuran-3-carboxamide (12) - $C_{24}H_{20}N_4O_2$

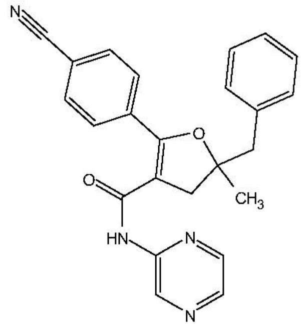

^{13}C NMR (100 MHz, $CDCl_3$): δ (ppm) 162.5 (C), 161.2 (C), 148.2 (C), 141.9 (CH_{Ar}), 140.0 (CH_{Ar}), 137.2 (CH_{Ar}), 135.9 (C), 134.4 (C), 132.0 ($2CH_{Ar}$), 130.5 ($2CH_{Ar}$), 130.0 ($2CH_{Ar}$), 128.5 ($2CH_{Ar}$), 127.3 (CH_{Ar}), 118.4 (C), 114.2 (C), 105.6 (C), 88.4 (C), 46.8 (CH_2), 42.1 (CH_2), 27.1 (CH_3).

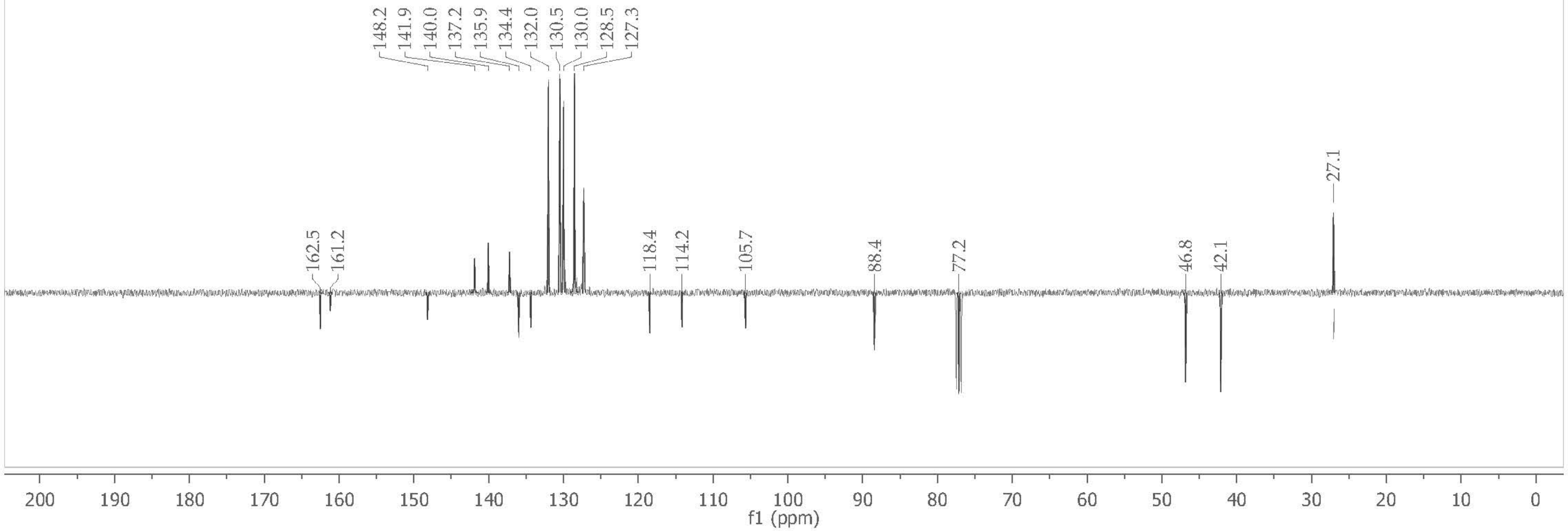

5-Benzyl-2-(4-cyanophenyl)-*N*-(1,5-dimethyl-1*H*-pyrazol-3-yl)-5-methyl-4,5-dihydrofuran-3-carboxamide (13) - $C_{25}H_{24}N_4O_2$

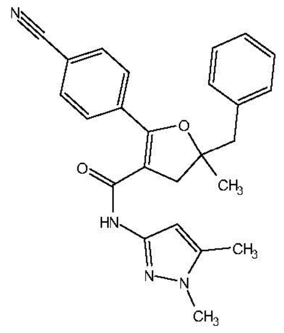

^{1}H NMR (400 MHz, $CDCl_3$): δ (ppm) 7.84 (d, $^3J_{H\text{-}H}$ = 8.4 Hz, 2H, 2CH_{Ar}), 7.64 (d, $^3J_{H\text{-}H}$ = 8.4 Hz, 2H, 2CH_{Ar}), 7.60 (br s, 1H, NH), 7.30-7.18 (m, 5H, 5CH_{Ar}), 6.43 (s, 1H, CH_{Ar}), 3.62 (s, 3H, CH_3), 3.15 (d, $^2J_{H\text{-}H}$ = 14.4 Hz, 1H, H-(CH_2), 3.04 (d, $^2J_{H\text{-}H}$ = 14.2 Hz, 1H, H-(CH_2), 3.00 (d, $^2J_{H\text{-}H}$ = 14.2 Hz, 1H, H-(CH_2), 2.85 (d, $^2J_{H\text{-}H}$ = 14.4 Hz, 1H, H-(CH_2), 2.21 (s, 3H, CH_3), 1.49 (s, 3H, CH_3).

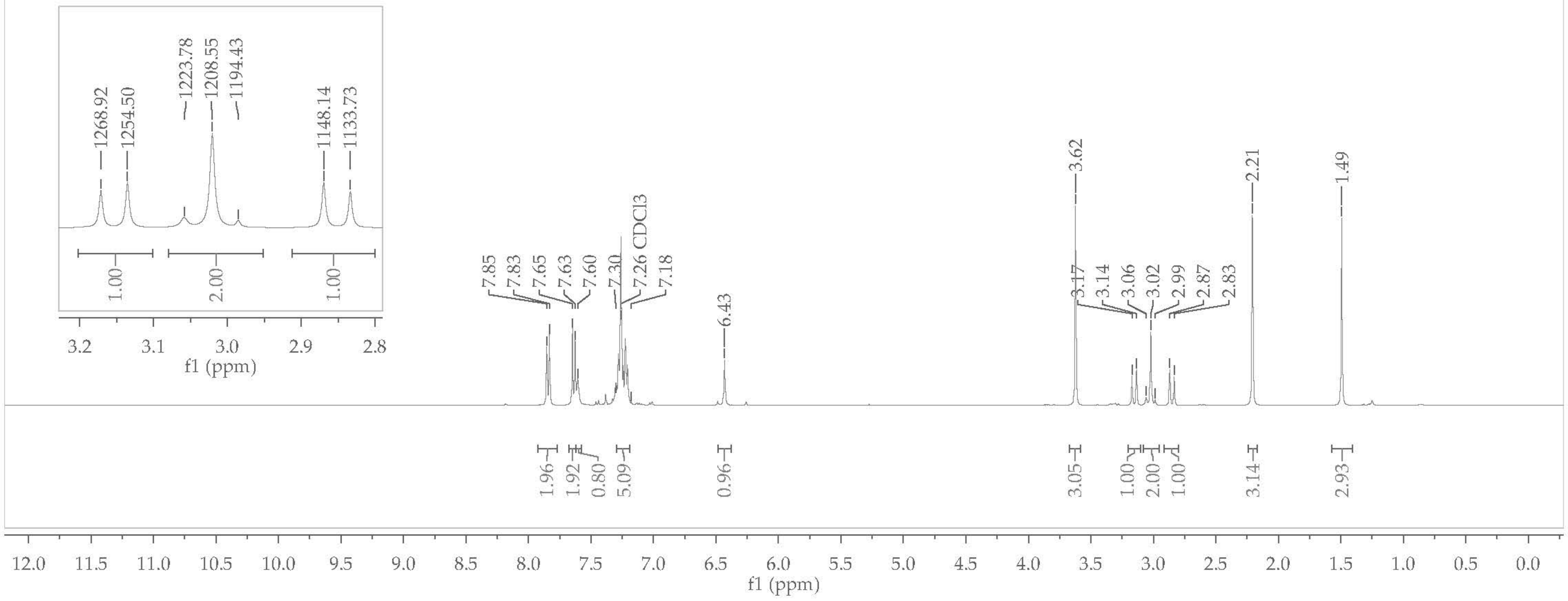

5-Benzyl-2-(4-cyanophenyl)-*N*-(1,5-dimethyl-1*H*-pyrazol-3-yl)-5-methyl-4,5-dihydrofuran-3-carboxamide (13) - $C_{25}H_{24}N_4O_2$

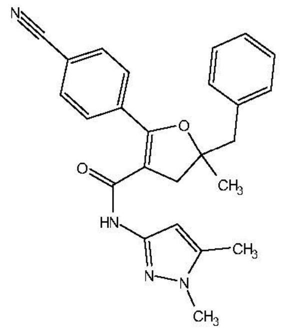

^{13}C NMR (100 MHz, $CDCl_3$) δ (ppm) 161.7 (C), 159.2 (C), 145.3 (C), 139.9 (C), 136.1 (C), 134.7 (C), 131.7 ($2CH_{Ar}$), 130.4 ($2CH_{Ar}$), 129.8 ($2CH_{Ar}$), 128.4 ($2CH_{Ar}$), 127.0 (CH_{Ar}), 118.6 (C), 113.4 (C), 106.0 (C), 97.4 (CH_{Ar}), 87.6 (C), 46.7 (CH_2), 42.4 (CH_2), 35.6 (CH_3), 26.9 (CH_3), 11.4 (CH_3).

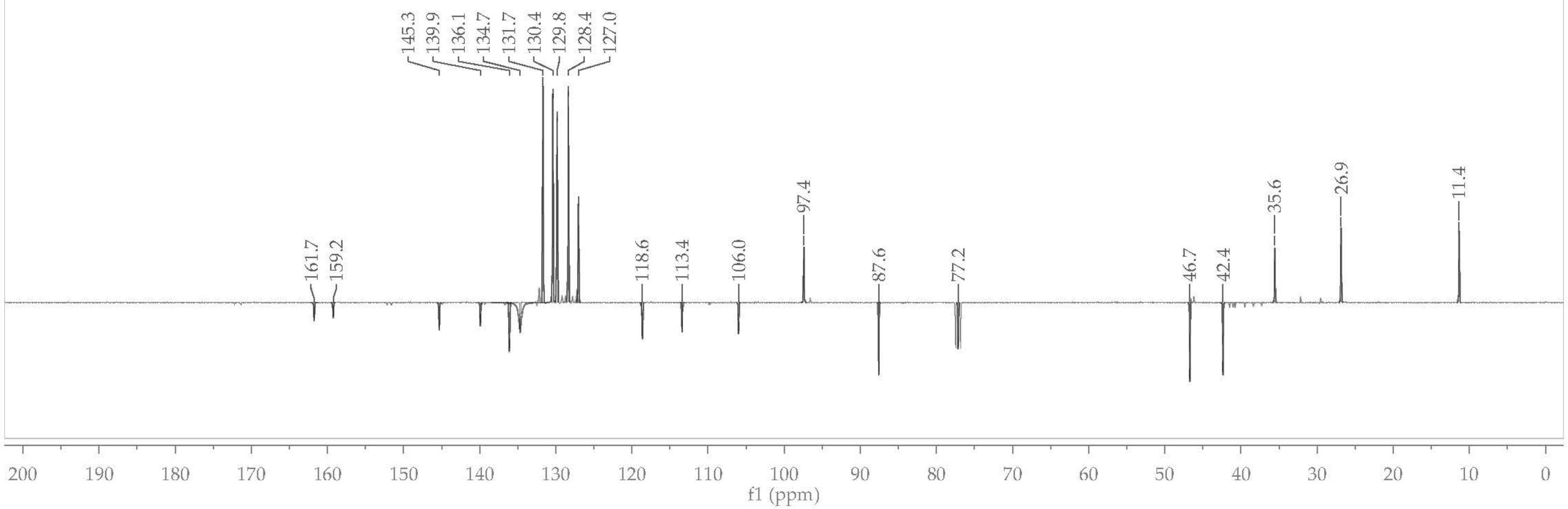

5-Benzyl-*N*-(5-bromopyridin-2-yl)-2-(4-(*N′*-hydroxycarbamimidoyl)phenyl)-5-methyl-4,5-dihydrofuran-3-carboxamide (14) - $C_{25}H_{23}BrN_4O_3$

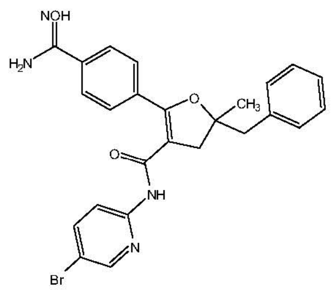

^{1}H NMR (400 MHz, $CDCl_3$): δ (ppm) 8.21 (d, $^4J_{H-H}$ = 2.5 Hz, 1H, CH_{Ar}), 8.12 (d, $^3J_{H-H}$ = 8.9 Hz, 1H, CH_{Ar}), 7.80 (br s, 1H, NH), 7.72 (dd, $^4J_{H-H}$ = 2.5 Hz, $^3J_{H-H}$= 8.9 Hz, 1H, CH_{Ar}), 7.69 (d, $^3J_{H-H}$ = 8.7 Hz, 2H, $2CH_{Ar}$), 7.66 (d, $^3J_{H-H}$ = 8.7 Hz, 2H, $2CH_{Ar}$), 7.33-7.19 (m, 5H, $5CH_{Ar}$), 5.10 (br s, 2H, NH_2), 3.17 (d, $^2J_{H-H}$ = 14.3 Hz, 1H, H-(CH_2), 2.99 (s, 2H, CH_2), 2.87 (d, $^2J_{H-H}$ = 14.3 Hz, 1H, H-(CH_2), 1.46 (s, 3H, CH_3). OH not observed.

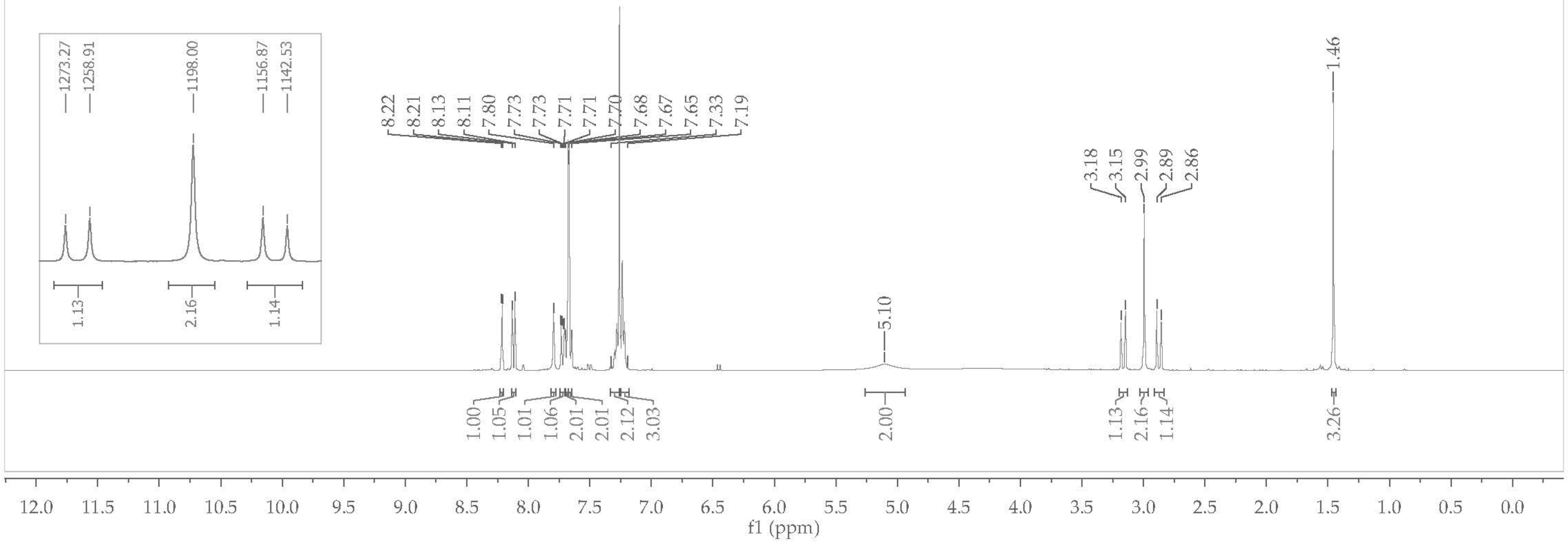

5-Benzyl-*N*-(5-bromopyridin-2-yl)-2-(4-(*N'*-hydroxycarbamimidoyl)phenyl)-5-methyl-4,5-dihydrofuran-3-carboxamide (14) - $C_{25}H_{23}BrN_4O_3$

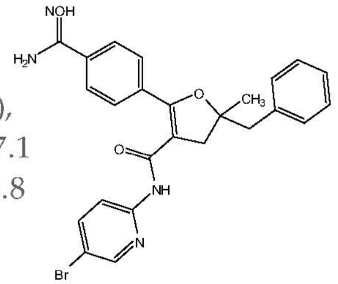

^{13}C NMR (100 MHz, $CDCl_3$): δ (ppm) 163.4 (C), 161.5 (C), 152.6 (C), 150.4 (C), 148.6 (CH_{Ar}), 140.8 (CH_{Ar}), 136.3 (C), 133.9 (C), 131.9 (C), 130.5 ($2CH_{Ar}$), 129.6 ($2CH_{Ar}$), 128.4 ($2CH_{Ar}$),127.1 (CH_{Ar}), 126.0 ($2CH_{Ar}$), 115.5 (CH_{Ar}), 114.2 (C), 105.2 (C), 87.8 (C), 46.8 (CH_2), 42.3 (CH_2), 26.8 (CH_3).

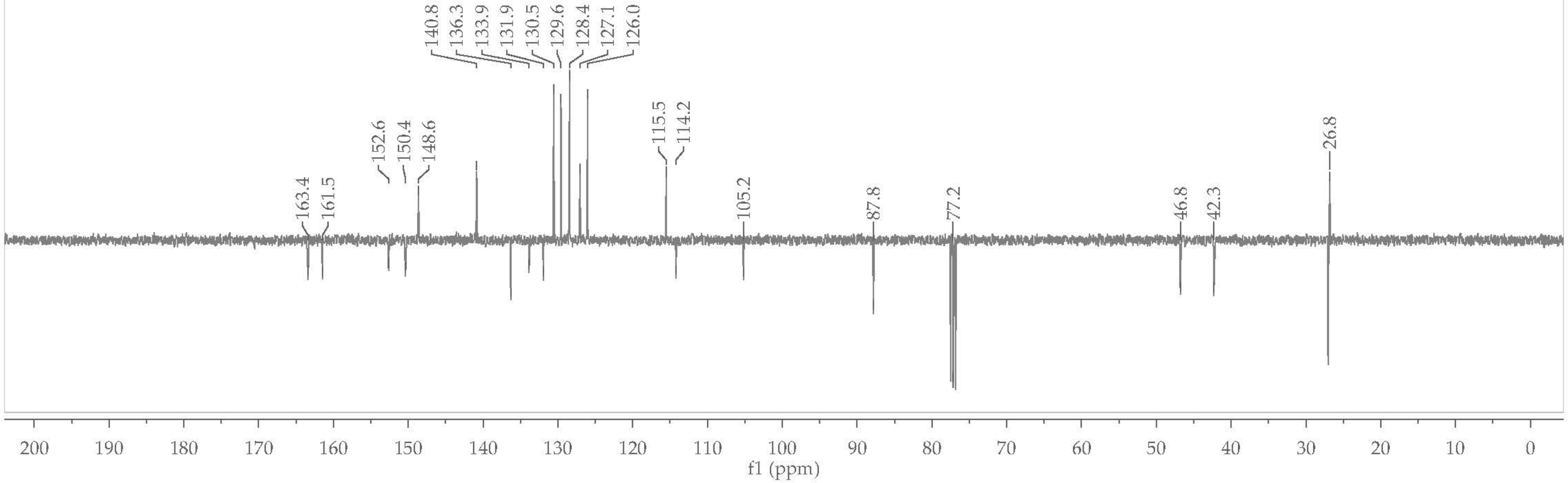

5-Benzyl-*N*-(5-chloropyridin-2-yl)-2-(4-(*N′*-hydroxycarbamimidoyl)phenyl)-5-methyl-4,5-dihydrofuran-3-carboxamide (15) - $C_{25}H_{23}ClN_4O_3$

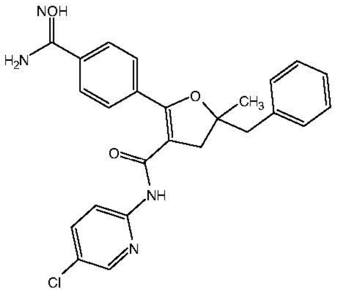

^{1}H NMR (400 MHz, $CDCl_3$): δ (ppm): 8.17 (d, $^3J_{H-H}$ = 8.9 Hz, 1H, CH_{Ar}), 8.12 (d, $^4J_{H-H}$ = 2.6 Hz, 1H, CH_{Ar}), 7.88 (br s, 1H, NH), 7.69 (d, $^3J_{H-H}$ = 8.9 Hz, 2H, $2CH_{Ar}$), 7.66 (d, $^3J_{H-H}$ = 8.9 Hz, 2H, $2CH_{Ar}$), 7.58 (dd, $^4J_{H-H}$ = 2.6 Hz, $^3J_{H-H}$= 8.9 Hz, 1H, CH_{Ar}), 7.31-7.18 (m, 5H, $5CH_{Ar}$), 5.06 (br s, 2H, NH_2), 3.15 (d, $^2J_{H-H}$ = 14.4 Hz, 1H, H-(CH_2), 3.00 (d, $^2J_{H-H}$ = 14.2 Hz, 1H, H-(CH_2), 2.95 (d, $^2J_{H-H}$ = 14.2 Hz, 1H, H-(CH_2), 2.86 (d, $^2J_{H-H}$ = 14.4 Hz, 1H, H-(CH_2), 1.43 (s, 3H, CH_3). OH not observed.

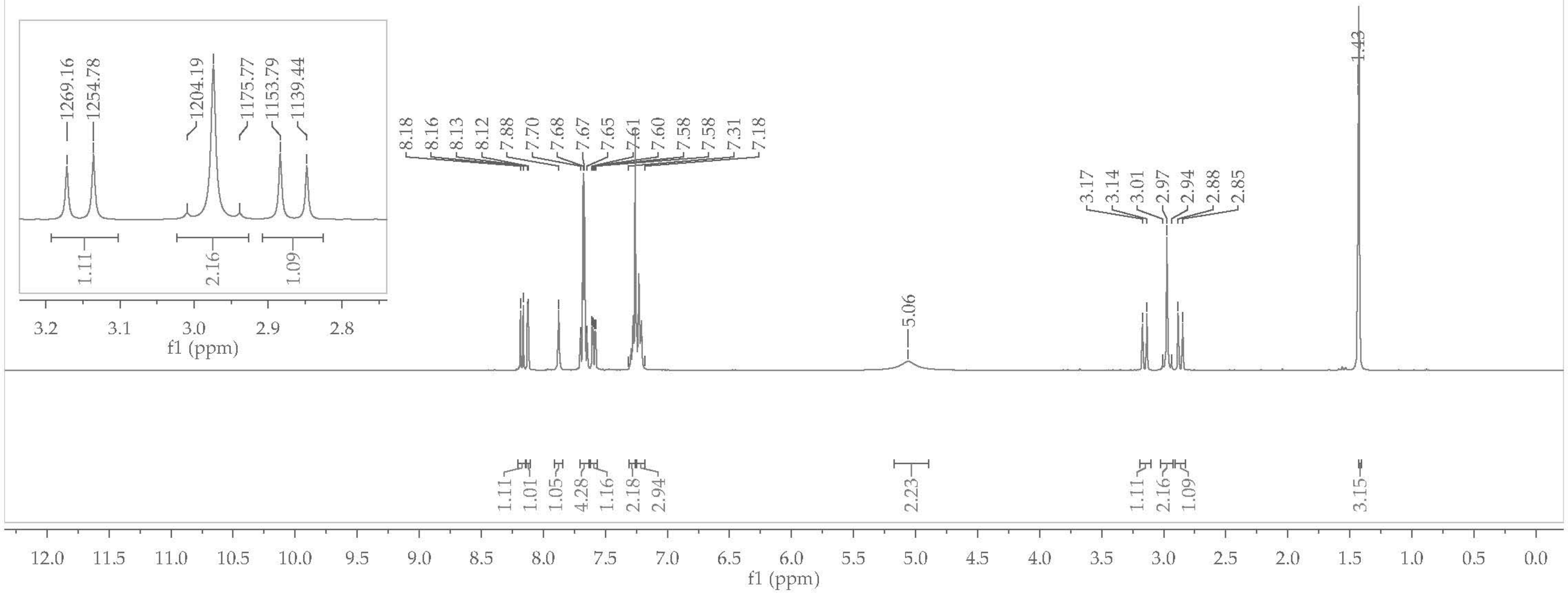

5-Benzyl-*N*-(5-chloropyridin-2-yl)-2-(4-(*N′*-hydroxycarbamimidoyl)phenyl)-5-methyl-4,5-dihydrofuran-3-carboxamide (15) - $C_{25}H_{23}ClN_4O_3$

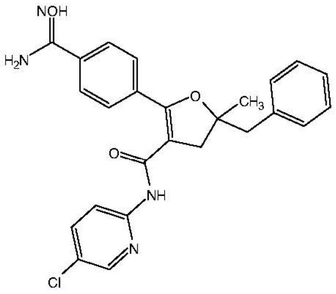

^{13}C NMR (100 MHz, $CDCl_3$): δ (ppm) 163.4 (C), 161.4 (C), 152.5 (C), 150.3 (C), 146.3 (CH_{Ar}), 138.1 (CH_{Ar}), 136.3 (C), 134.0 (C), 131.8 (C), 130.5 ($2CH_{Ar}$), 129.6 ($2CH_{Ar}$), 128.4 ($2CH_{Ar}$),127.0 (CH_{Ar}), 126.4 (C), 125.9 ($2CH_{Ar}$), 115.5 (CH_{Ar}), 105.1 (C), 87.8 (C), 46.8 (CH_2), 42.3 (CH_2), 26.8 (CH_3).

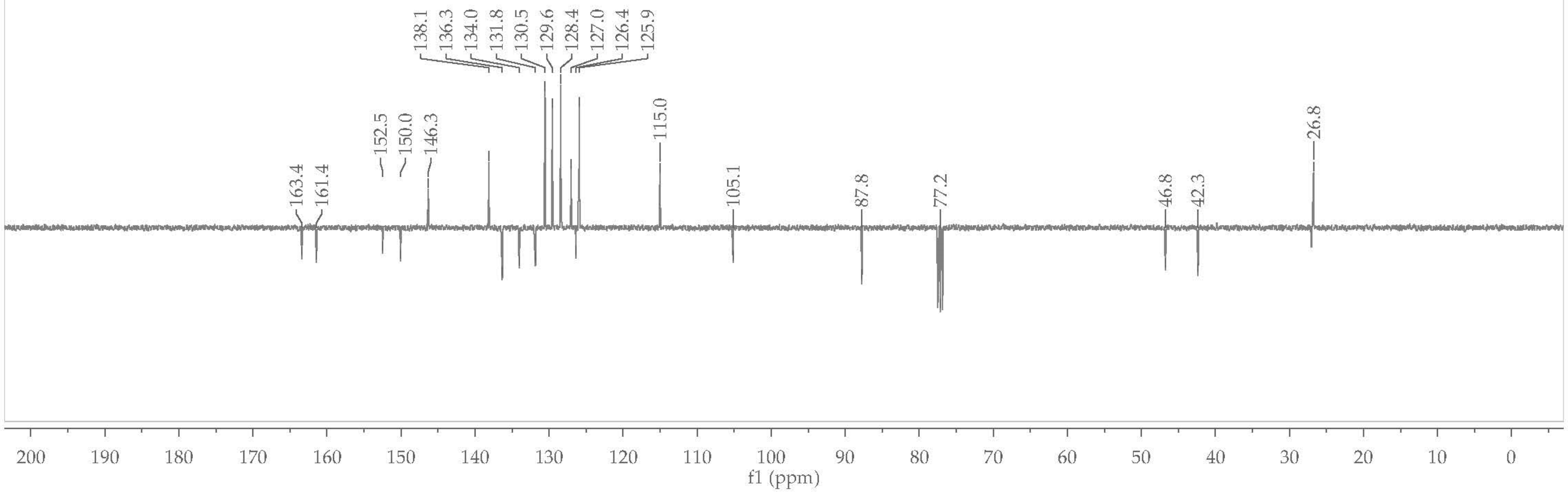

5-Benzyl-2-(4-(*N′*-hydroxycarbamimidoyl)phenyl)-5-methyl-N-(5-methylpyridin-2-yl)-4,5-dihydrofuran-3-carboxamide (16) - $C_{26}H_{26}N_4O_3$

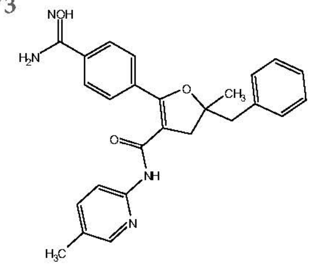

^{1}H NMR (400 MHz, $CDCl_3$) δ (ppm): 8.22-8.10 (m, 2H, 2CH_{Ar}), 8.04 (br s, 1H, NH), 7.74 (d, $^3J_{H-H}$ = 8.5 Hz, 2H, 2CH_{Ar}), 7.68 (d, $^3J_{H-H}$ = 8.5 Hz, 2H, 2CH_{Ar}), 7.49 (d, $^3J_{H-H}$ = 8.8 Hz, 1H, CH_{Ar}), 7.32-7.18 (m, 5H, 5CH_{Ar}), 4.98 (br s, 2H, NH_2), 3.15 (d, $^2J_{H-H}$ = 14.4 Hz, 1H, H-(CH_2)), 2.96 (d, $^2J_{H-H}$ = 13.8 Hz, 1H, H-(CH_2)), 2.88 (d, $^2J_{H-H}$ = 13.4 Hz, 1H, H-(CH_2)), 2.86 (d, $^2J_{H-H}$ = 14.4 Hz, 1H, H-(CH_2)), 2.28 (s, 3H, CH_3), 1.36 (s, 3H, CH_3). OH not observed.

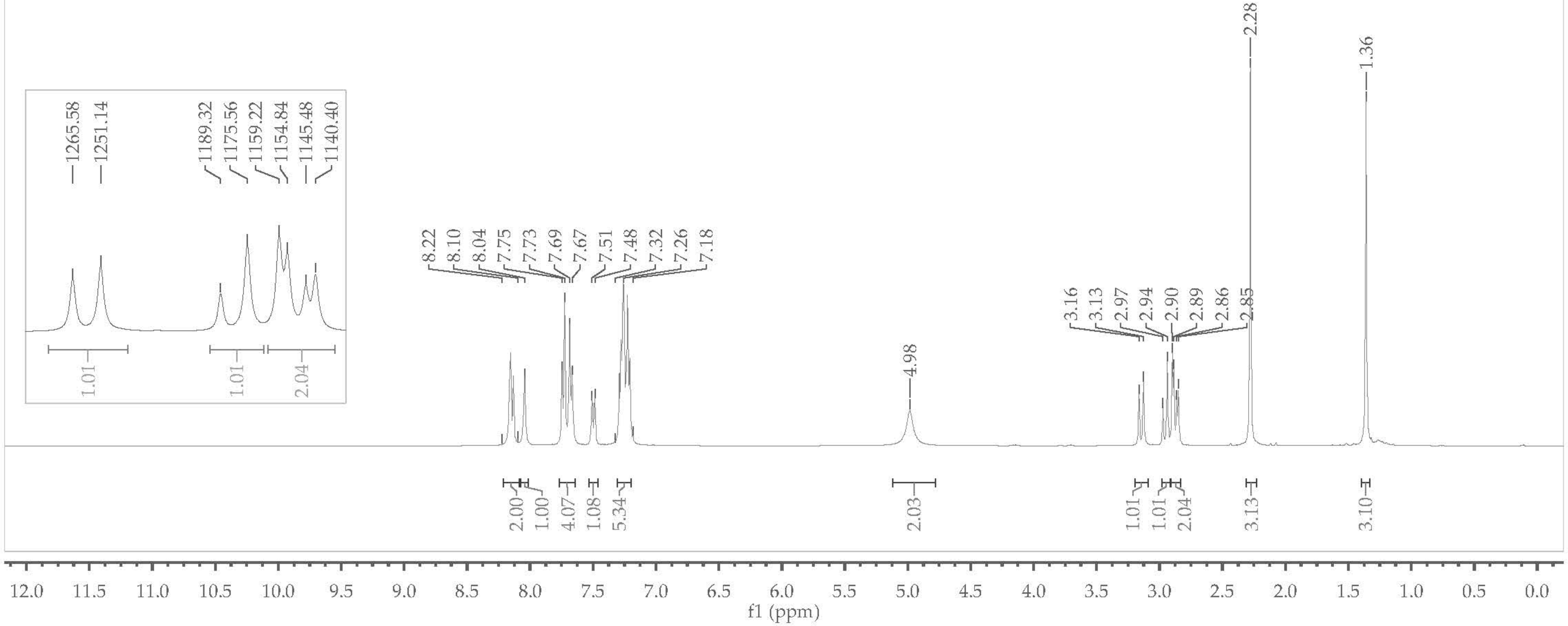

5-Benzyl-2-(4-(*N'*-hydroxycarbamimidoyl)phenyl)-5-methyl-N-(5-methylpyridin-2-yl)-4,5-dihydrofuran-3-carboxamide (16) - $C_{26}H_{26}N_4O_3$

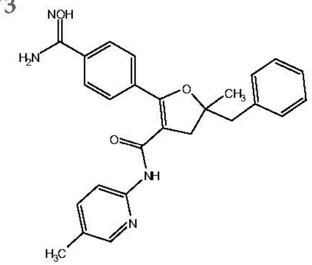

^{13}C NMR (100 MHz, $CDCl_3$) δ (ppm): 163.6 (C), 160.9 (C), 152.3 (C), 149.5 (C), 147.1 (CH_{Ar}), 139.3 (CH_{Ar}), 136.4 (C), 134.2 (C), 131.9 (C), 130.5 ($2CH_{Ar}$), 129.5 ($2CH_{Ar}$), 128.8 (C), 128.3 ($2CH_{Ar}$), 126.9 (CH_{Ar}), 125.8 ($2CH_{Ar}$), 114.2 (CH_{Ar}), 105.3 (C), 87.4 (C), 46.7 (CH_2), 42.6 (CH_2), 26.5 (CH_3), 17.9 (CH_3).

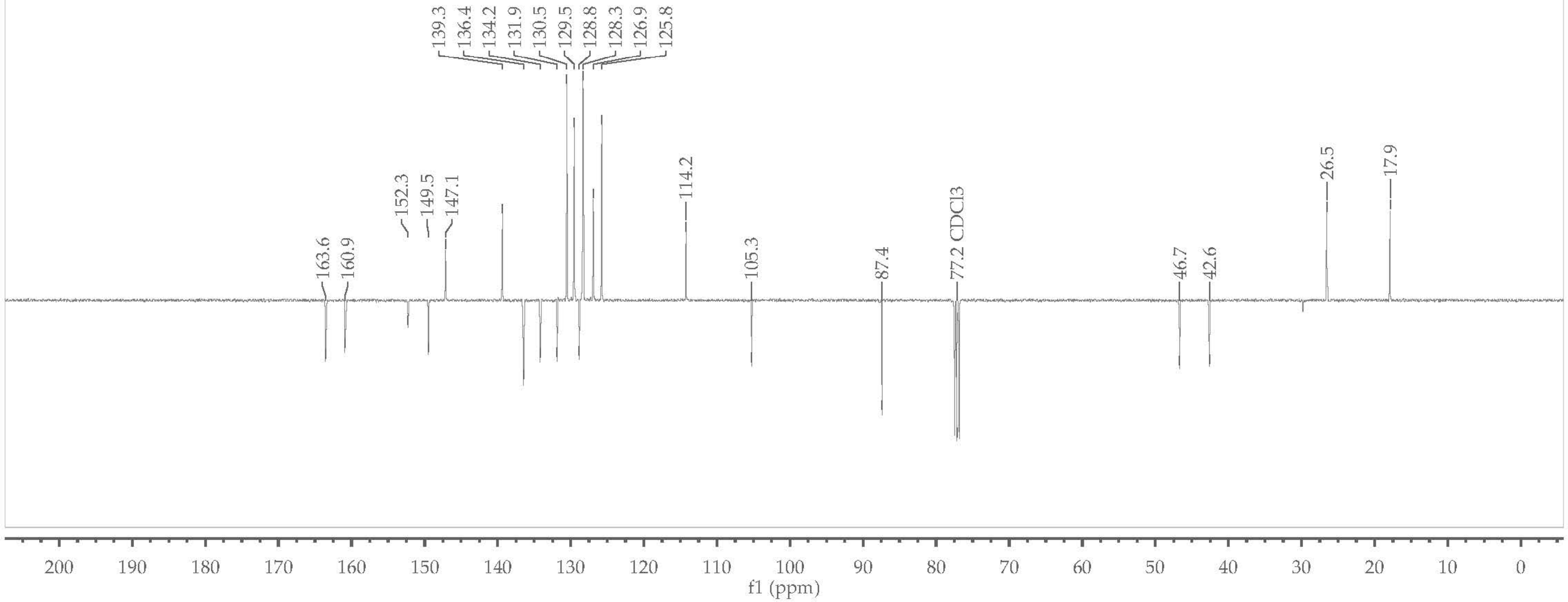

5-benzyl-2-(4-(*N'*-hydroxycarbamimidoyl)phenyl)-5-methyl-*N*-(pyridin-2-yl)-4,5-dihydrofuran-3-carboxamide (17) - $C_{25}H_{24}N_4O_3$

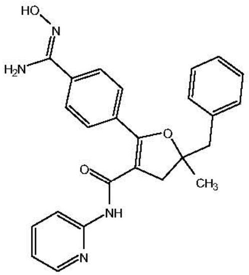

^{1}H NMR (400 MHz, $CDCl_3$): δ (ppm) 8.46 (br s, 1H, NH), 7.23 (d, $^3J_{H-H}$ = 8.6 Hz, 1H, CH_{Ar}), 8.17-8.14 (m, 1H, CH_{Ar}), 7.75-7.62 (m, 5H, $5CH_{Ar}$), 7.31-7.19 (m, 5H, $5CH_{Ar}$), 7.04-6.97 (m, 1H, CH_{Ar}), 5.65 (br s, 2H, NH_2), 3.24 (d, $^2J_{H-H}$ = 14.4 Hz, 1H, H-(CH_2)), 3.01 (d, $^2J_{H-H}$ = 14.2 Hz, 1H, H-(CH_2)), 2.98 (d, $^2J_{H-H}$ = 14.2 Hz, 1H, H-(CH_2)), 2.96 (d, $^2J_{H-H}$ = 14.4 Hz, 1H, H-(CH_2)), 1.45 (s, 3H, CH_3). OH not observed.

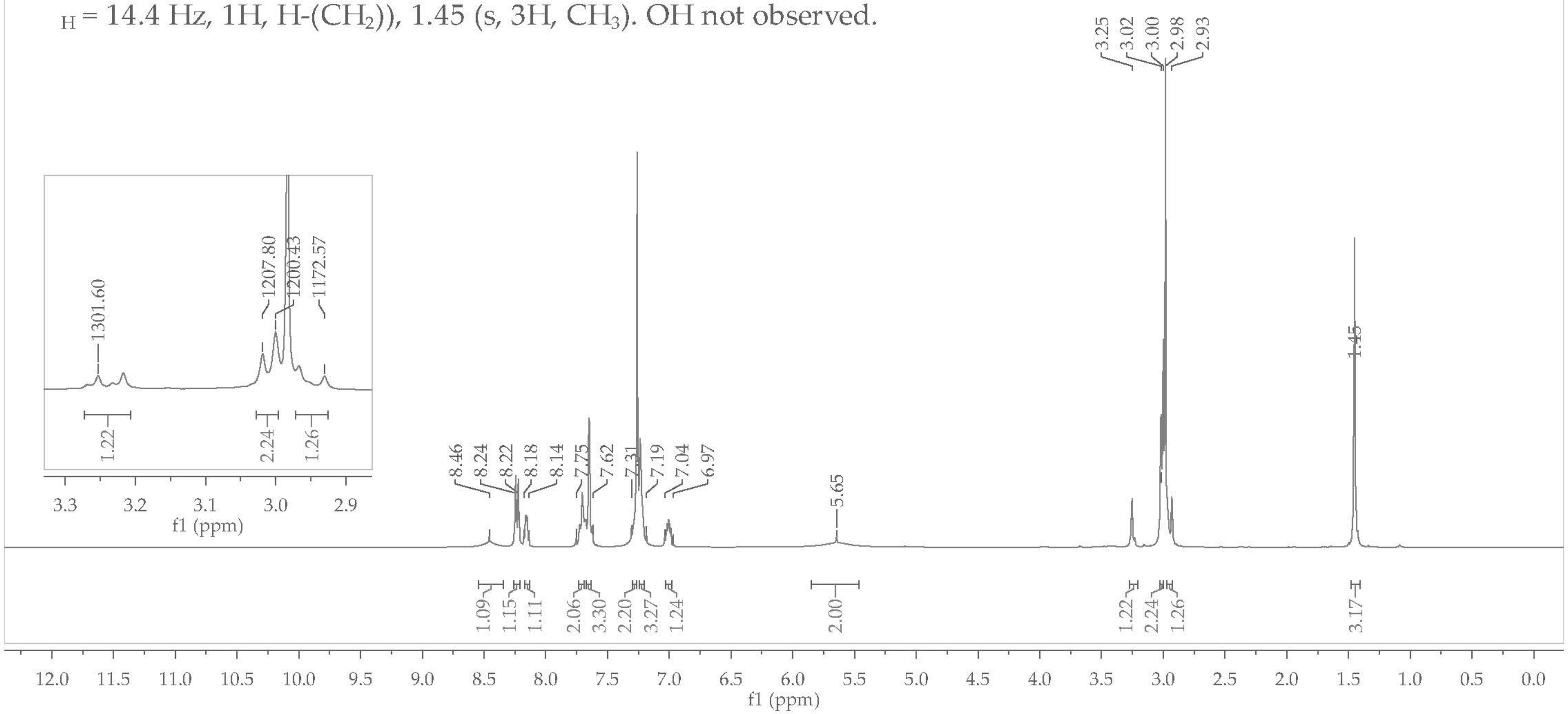

5-benzyl-2-(4-(*N'*-hydroxycarbamimidoyl)phenyl)-5-methyl-*N*-(pyridin-2-yl)-4,5-dihydrofuran-3-carboxamide (17) - $C_{25}H_{24}N_4O_3$

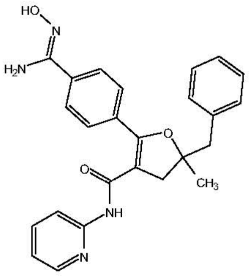

^{13}C NMR (100 MHz, $CDCl_3$): δ (ppm) 163.8 (C), 161.9 (C), 157.3 (C), 151.2 (C), 141.7 (C), 139.3 (CH_{Ar}), 136.3 (C), 132.3 (C), 130.6 (2CH_{Ar}), 129.7 (2CH_{Ar}), 128.4 (2CH_{Ar}), 127.0 (CH_{Ar}), 126.8 (CH_{Ar}), 126.3 (2CH_{Ar}), 119.5 (CH_{Ar}), 115.1 (CH_{Ar}), 105.2 (C), 88.1 (C), 46.8 (CH_2), 42.3 (CH_2), 26.8 (CH_3).

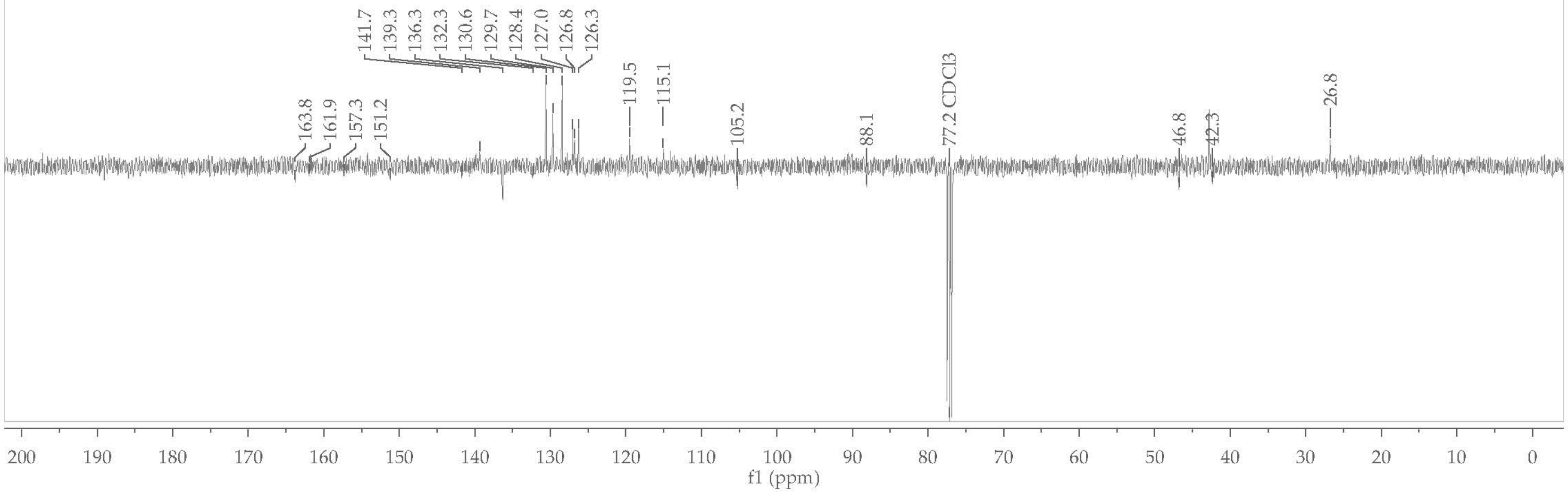

5-benzyl-2-(4-(*N′*-hydroxycarbamimidoyl)phenyl)-5-methyl-*N*-(pyridin-4-yl)-4,5-dihydrofuran-3-carboxamide (18) - $C_{25}H_{24}N_4O_3$

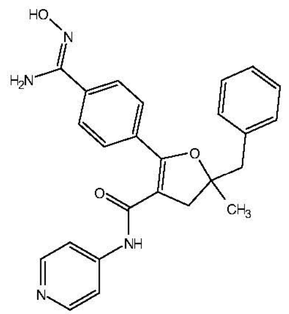

^{1}H NMR (400 MHz, $CDCl_3$): δ (ppm) 8.34 (d, $^3J_{H\text{-}H}$ = 6.4 Hz, 2H, 2CH_{Ar}), 7.72 (d, $^3J_{H\text{-}H}$ = 8.5 Hz, 2H, 2CH_{Ar}), 7.59 (d, $^3J_{H\text{-}H}$ = 8.5 Hz, 2H, 2CH_{Ar}), 7.34-7.22 (m, 5H, 5CH_{Ar}), 7.17 (d, $^3J_{H\text{-}H}$ = 6.4 Hz, 2H, 2CH_{Ar}), 7.08 (br s, 1H, NH), 4.95 (br s, 2H, NH_2), 3.19 (d, $^2J_{H\text{-}H}$ = 14.8 Hz, 1H, H-(CH_2), 3.06 (d, $^2J_{H\text{-}H}$ = 13.8 Hz, 1H, H-(CH_2), 3.01 (d, $^2J_{H\text{-}H}$ = 13.8 Hz, 1H, H-(CH_2), 2.93 (d, $^2J_{H\text{-}H}$ = 14.8 Hz, 1H, H-(CH_2)), 1.52 (s, 3H, CH_3). OH not observed.

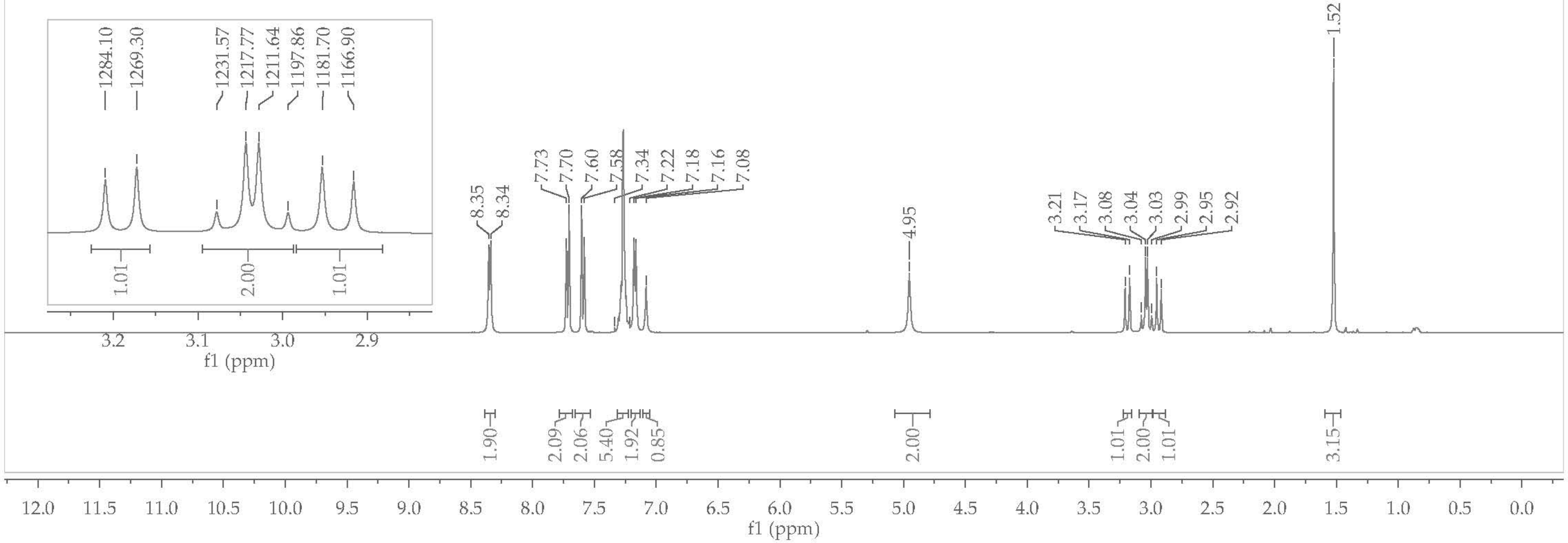

5-benzyl-2-(4-(*N′*-hydroxycarbamimidoyl)phenyl)-5-methyl-*N*-(pyridin-4-yl)-4,5-dihydrofuran-3-carboxamide (18) - $C_{25}H_{24}N_4O_3$

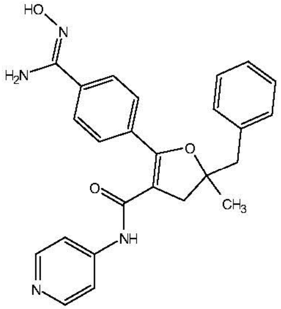

^{13}C NMR (100 MHz, $CDCl_3$): δ (ppm) 163.8 (C), 160.0 (C), 151.7 (C), 150.3 (2CH_{Ar}), 145.3 (C), 136.4 (C), 135.1 (C), 131.3 (C), 130.6 (2CH_{Ar}), 129.3 (2CH_{Ar}), 128.4 (2CH_{Ar}), 127.0 (CH_{Ar}), 126.2 (2CH_{Ar}), 113.3 (2CH_{Ar}), 106.5 (C), 88.1 (C), 46.9 (CH_2), 42.3 (CH_2), 27.0 (CH_3).

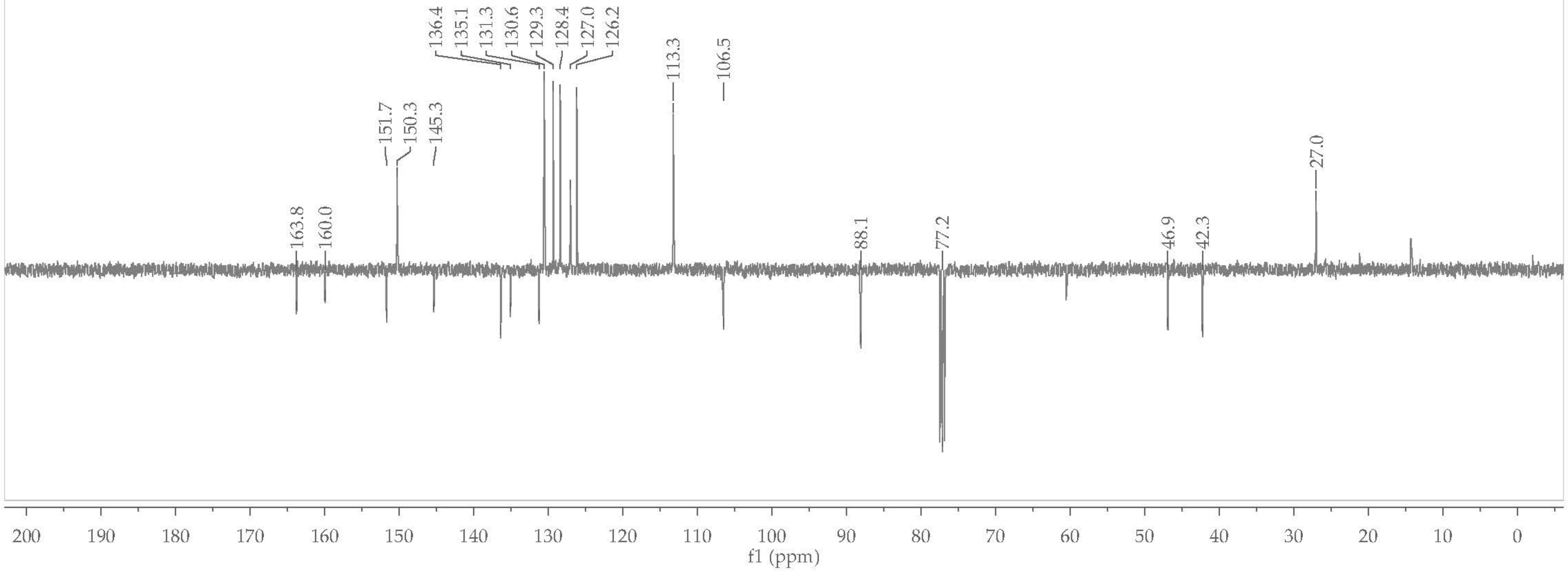

5-Benzyl-2-(4-(*N'*-hydroxycarbamimidoyl)phenyl)-5-methyl-*N*-phenyl-4,5-dihydrofuran-3-carboxamide (19) - $C_{26}H_{25}N_3O_3$

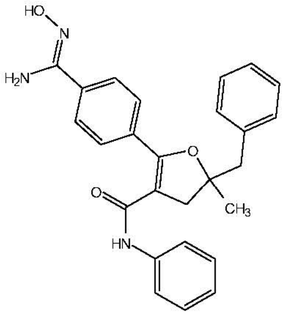

^{1}H NMR (400 MHz, $CDCl_3$): δ (ppm) 7.69 (d, $^3J_{H-H}$ = 8.4 Hz, 2H, 2CH_{Ar}), 7.67 (d, $^3J_{H-H}$ = 8.4 Hz, 2H, 2CH_{Ar}), 7.32-7.22 (m, 9H, 9CH_{Ar}), 7.04 (t, $^3J_{H-H}$ = 6.9 Hz, 1H, CH_{Ar}), 6.83 (br s, 1H, NH), 4.96 (s, 2H, NH_2), 3.19 (d, $^2J_{H-H}$ = 14.8 Hz, 1H, H-(CH_2), 3.06 (d, $^2J_{H-H}$ = 13.8 Hz, 1H, H-(CH_2), 3.03 (d, $^2J_{H-H}$ = 13.8 Hz, 1H, H-(CH_2), 2.95 (d, $^2J_{H-H}$ = 14.8 Hz, 1H, H-(CH_2), 1.53 (s, 3H, CH_3). OH not observed.

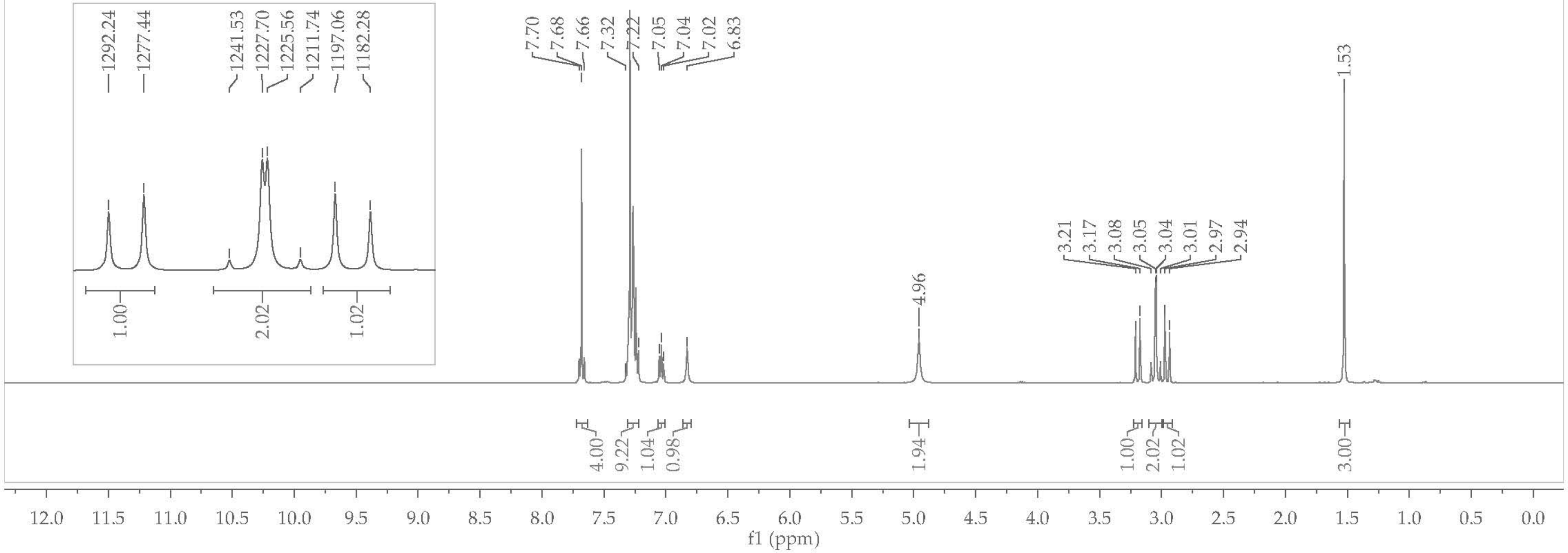

5-Benzyl-2-(4-(*N'*-hydroxycarbamimidoyl)phenyl)-5-methyl-*N*-phenyl-4,5-dihydrofuran-3-carboxamide (19) - $C_{26}H_{25}N_3O_3$

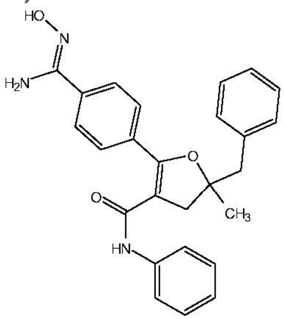

^{13}C NMR (100 MHz, $CDCl_3$) δ (ppm) 163.5 (C), 158.3 (C), 152.1 (C), 138.0 (C), 136.6 (C), 134.1 (C), 131.8 (C), 130.6 ($2CH_{Ar}$), 129.4 ($2CH_{Ar}$), 129.1 ($2CH_{Ar}$), 128.3 ($2CH_{Ar}$), 126.9 (CH_{Ar}), 126.0 ($2CH_{Ar}$), 124.0 (CH_{Ar}), 119.6 ($2CH_{Ar}$), 106.7 (C), 87.4 (C), 47.0 (CH_2), 42.7 (CH_2), 27.0 (CH_3).

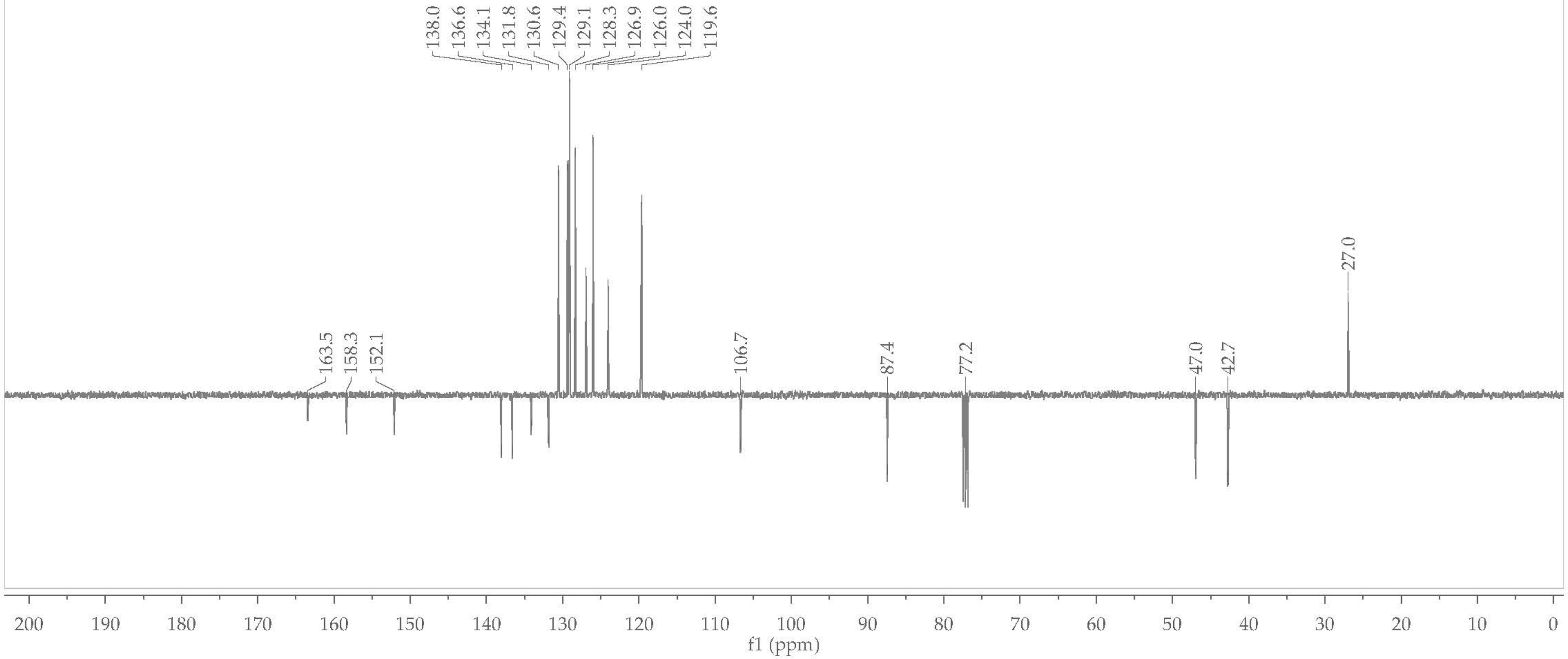

5-Benzyl-*N*-(4-fluorophenyl)-2-(4-(*N′*-hydroxycarbamimidoyl)phenyl)-5-methyl-4,5-dihydrofuran-3-carboxamide (20) - $C_{26}H_{24}FN_3O_3$

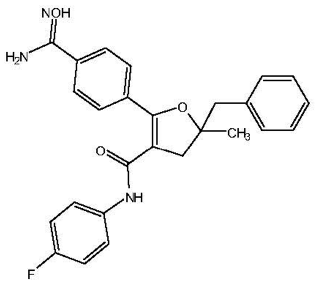

^{1}H NMR (400 MHz, $CDCl_3$): δ (ppm) 7.70-7.59 (m, 4H, 4CH_{Ar}), 7.29-7.17 (m, 7H, 7CH_{Ar}), 6.91 (t, $^{3}J_{H\text{-}H}$ = 8.6 Hz, 2H, 2CH_{Ar}), 6.73 (br s, 1H, NH), 4.92 (br s, 2H, NH_2), 3.15 (d, $^{2}J_{H\text{-}H}$ = 14.8 Hz, 1H, H-(CH_2), 3.04 (d, $^{2}J_{H\text{-}H}$ = 13.9 Hz, 1H, H-(CH_2), 2.99 (d, $^{2}J_{H\text{-}H}$ = 13.9 Hz, 1H, H-(CH_2), 2.91 (d, $^{2}J_{H\text{-}H}$ = 14.8 Hz, 1H, H-(CH_2), 1.50 (s, 3H, CH_3). OH not observed.

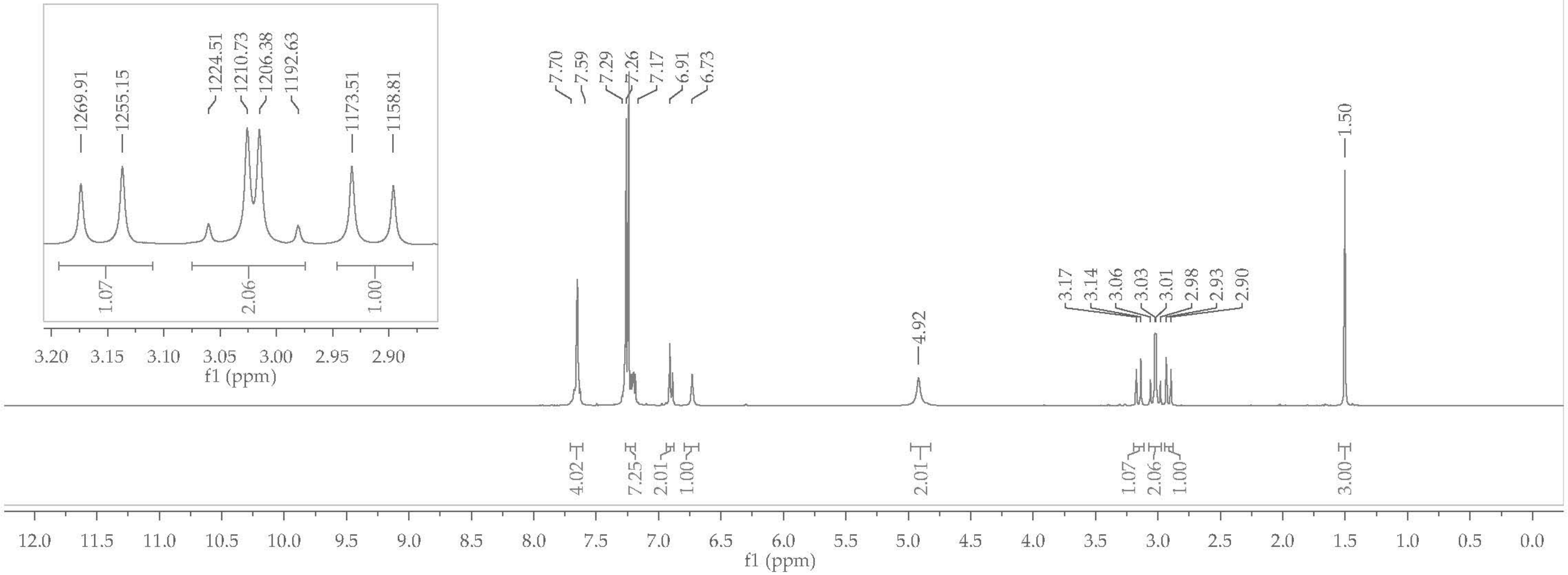

5-Benzyl-*N*-(4-fluorophenyl)-2-(4-(*N′*-hydroxycarbamimidoyl)phenyl)-5-methyl-4,5-dihydrofuran-3-carboxamide (20) - $C_{26}H_{24}FN_3O_3$

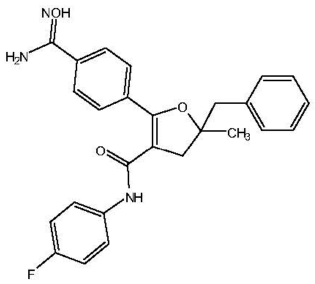

^{13}C NMR (100 MHz, $CDCl_3$): δ (ppm) 163.4 (C), 159.3 (d, $J_{C\text{-}F}$ = 243.0 Hz, C), 158.6 (C), 158.1 (C), 136.6 (C), 134.2 (C), 134.0 (d, $J_{C\text{-}F}$ = 2.8 Hz, C), 131.8 (C), 130.6 ($2CH_{Ar}$), 129.4 ($2CH_{Ar}$), 128.4 ($2CH_{Ar}$), 127.0 (CH_{Ar}), 126.0 ($2CH_{Ar}$), 121.4 (d, $J_{C\text{-}F}$ = 7.7 Hz, $2CH_{Ar}$), 115.7 (d, $J_{C\text{-}F}$ = 22.5 Hz, $2CH_{Ar}$), 106.4 (C), 87.5 (C), 47.0 (CH_2), 42.7 (CH_2), 27.0 (CH_3).

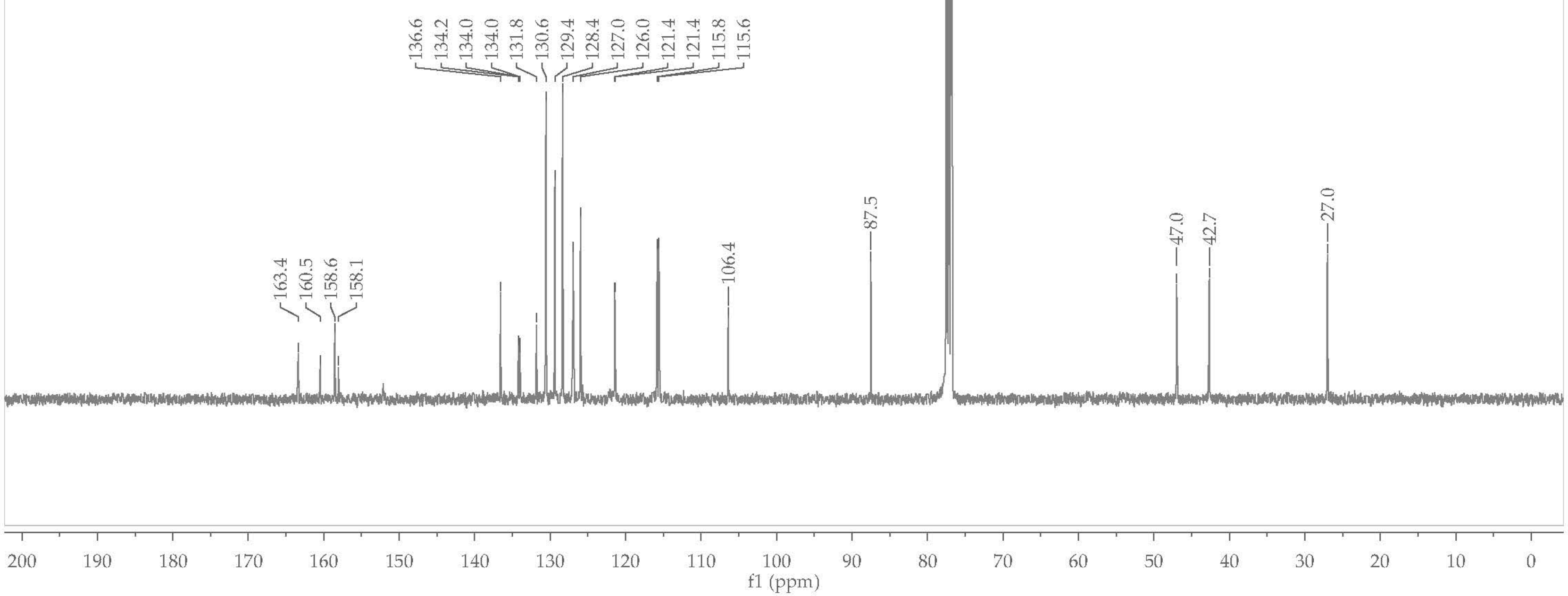

5-Benzyl-2-(4-(*N′*-hydroxycarbamimidoyl)phenyl)-5-methyl-N-(4-(trifluoromethyl)benzyl)-4,5-dihydrofuran-3-carboxamide (21) - $C_{28}H_{26}F_3N_3O_3$

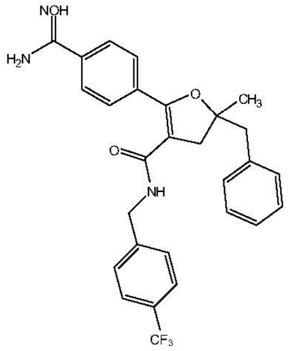

^{1}H NMR (400 MHz, $CDCl_3$): δ (ppm) 7.65-7.52 (m, 6H, 6CH_{Ar}), 7.33-7.16 (m, 7H, 7CH_{Ar}), 5.42 (t, $^3J_{H\text{-}H}$ = 5.8 Hz, 1H, NH), 4.87 (br s, 2H, NH_2), 4.46-4.35 (m, 2H, CH_2), 3.09 (d, $^2J_{H\text{-}H}$ = 14.7 Hz, 1H, H-(CH_2)), 3.03 (d, $^2J_{H\text{-}H}$ = 13.8 Hz, 1H, H-(CH_2)), 2.99 (d, $^2J_{H\text{-}H}$ = 13.8 Hz, 1H, H-(CH_2)), 2.85 (d, $^2J_{H\text{-}H}$ = 14.7 Hz, 1H, H-(CH_2)), 1.49 (s, 3H, CH_3). OH not observed.

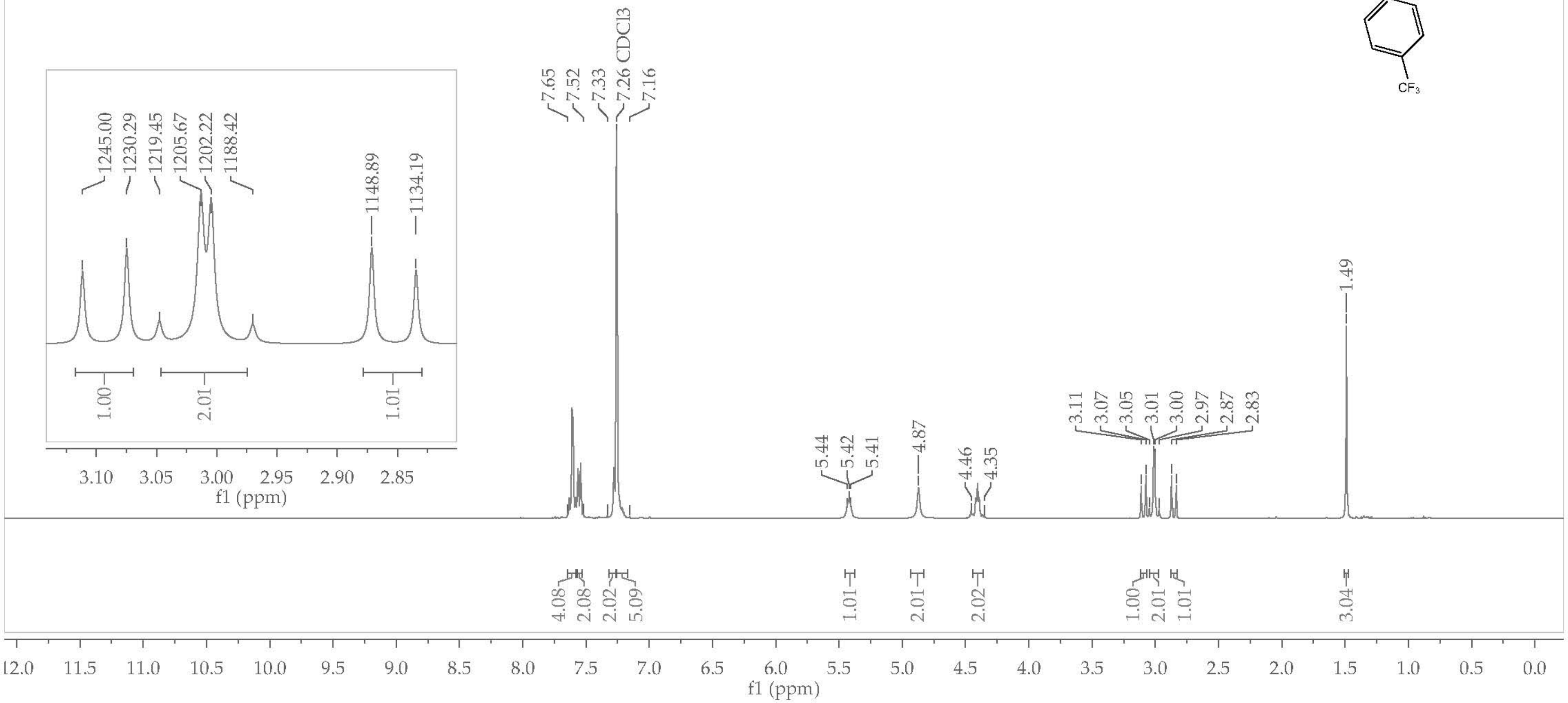

5-Benzyl-2-(4-(*N′*-hydroxycarbamimidoyl)phenyl)-5-methyl-N-(4-(trifluoromethyl)benzyl)-4,5-dihydrofuran-3-carboxamide (21) - $C_{28}H_{26}F_3N_3O_3$

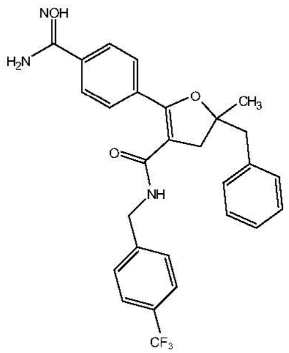

^{13}C NMR (100 MHz, $CDCl_3$): δ (ppm) 165.4 (C), 158.5 (C), 152.0 (C), 142.6 (C), 136.6 (C), 134.1 (C), 131.9 (C), 130.5 (2CH$_{Ar}$), 129.3 (2CH$_{Ar}$), 129.7 (q, $^2J_{C\text{-}F}$ = 32.5 Hz, C), 128.3 (2CH$_{Ar}$), 128.1 (2CH$_{Ar}$), 126.9 (CH$_{Ar}$), 125.7 (2CH$_{Ar}$), 125.6 (q, $^3J_{C\text{-}F}$ = 3.7 Hz, 2CH$_{Ar}$), 124.5 (q, $^1J_{C\text{-}F}$ = 271.5 Hz, CF_3), 105.4 (C), 82.7 (C), 46.9 (CH_2), 42.9 (CH_2), 42.7 (CH_2), 26.9 (CH_3).

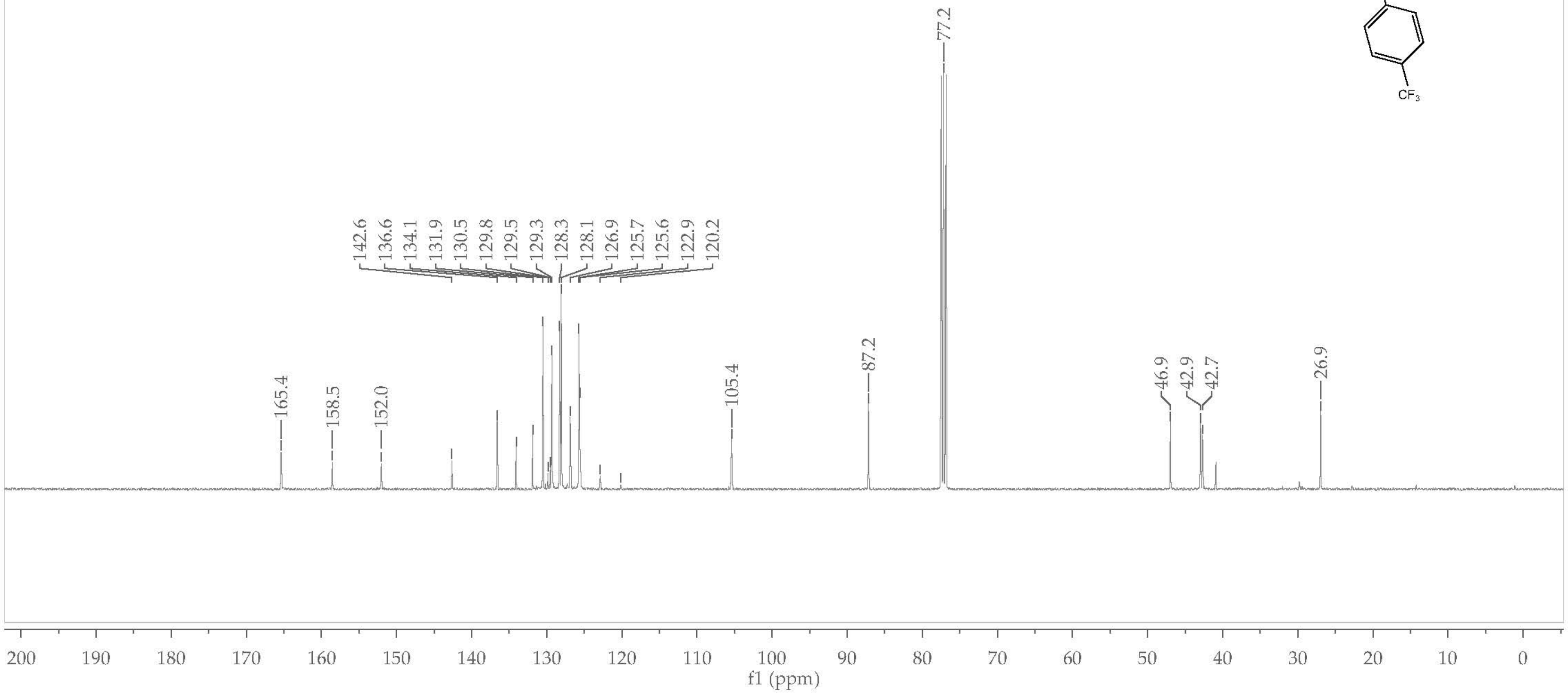

5-benzyl-2-(4-(*N′*-hydroxycarbamimidoyl)phenyl)-5-methyl-N-(pyridazin-3-yl)-4,5-dihydrofuran-3-carboxamide (22) - $C_{24}H_{23}N_5O_3$

^{1}H NMR (400 MHz, $CDCl_3$): δ (ppm) 8.80 (d, $^3J_{H\text{-}H}$ = 5.2 Hz, 1H, CH_{Ar}), 8.53 (br s, 1H, NH), 8.40 (d, $^3J_{H\text{-}H}$ = 9.6 Hz, 1H, CH_{Ar}), 7.67 (d, $^3J_{H\text{-}H}$ = 8.8 Hz, 2H, $2CH_{Ar}$), 7.67 (d, $^3J_{H\text{-}H}$ = 8.8 Hz, 2H, $2CH_{Ar}$), 7.38 (dd, $^3J_{H\text{-}H}$ = 9.6 Hz, $^3J_{H\text{-}H}$ = 5.2 Hz, 1H,CH_{Ar}), 7.28-7.19 (m, 5H, $5CH_{Ar}$), 5.17 (br s, 2H, NH_2), 3.19 (d, $^2J_{H\text{-}H}$ = 14.4 Hz, 1H, H-(CH_2)), 3.00 (d, $^2J_{H\text{-}H}$ = 14.3 Hz, 1H, H-(CH_2)), 2.96 (d, $^2J_{H\text{-}H}$ = 14.3 Hz, 1H, H-(CH_2)), 2.90 (d, $^2J_{H\text{-}H}$ = 14.4 Hz, 1H, H-(CH_2)), 1.43 (s, 3H, CH_3). OH not observed.

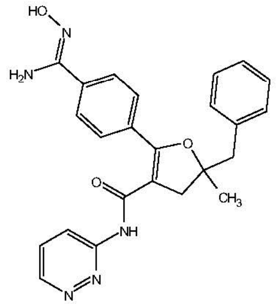

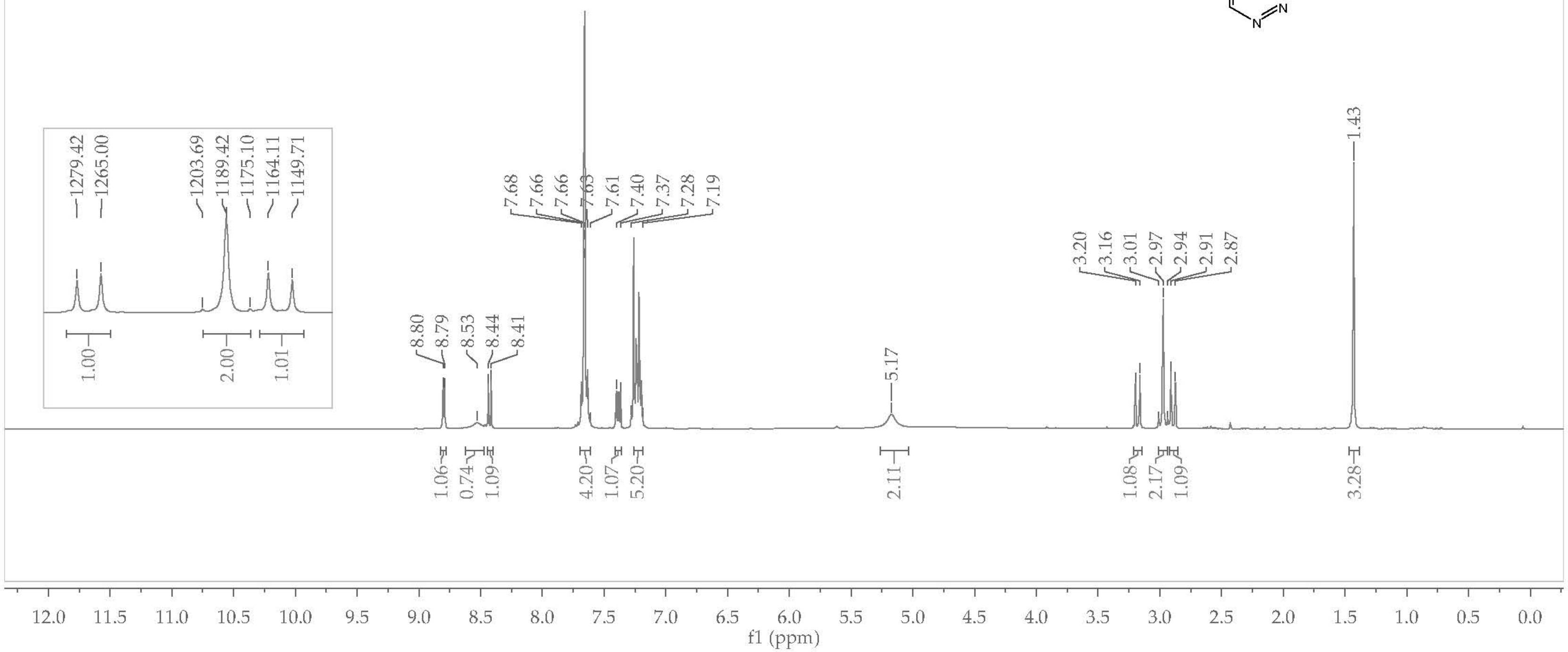

5-benzyl-2-(4-(*N′*-hydroxycarbamimidoyl)phenyl)-5-methyl-N-(pyridazin-3-yl)-4,5-dihydrofuran-3-carboxamide (22) - $C_{24}H_{23}N_5O_3$

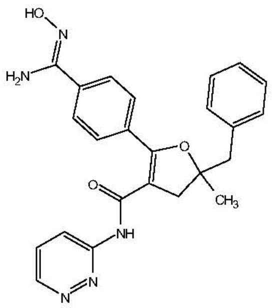

^{13}C NMR (100 MHz, $CDCl_3$): δ (ppm) 164.0 (C), 162.2 (C), 155.5 (C), 152.4 (C), 148.1 (CH_{Ar}), 136.2 (C), 134.5 (C), 131.5 (C), 130.5 (2CH_{Ar}), 129.4 (2CH_{Ar}), 128.4 (3CH_{Ar}), 127.0 (CH_{Ar}), 126.1 (2CH_{Ar}), 119.2 (CH_{Ar}), 104.9 (C), 88.0 (C), 46.7 (CH_2), 42.1 (CH_2), 27.6 (CH_3).

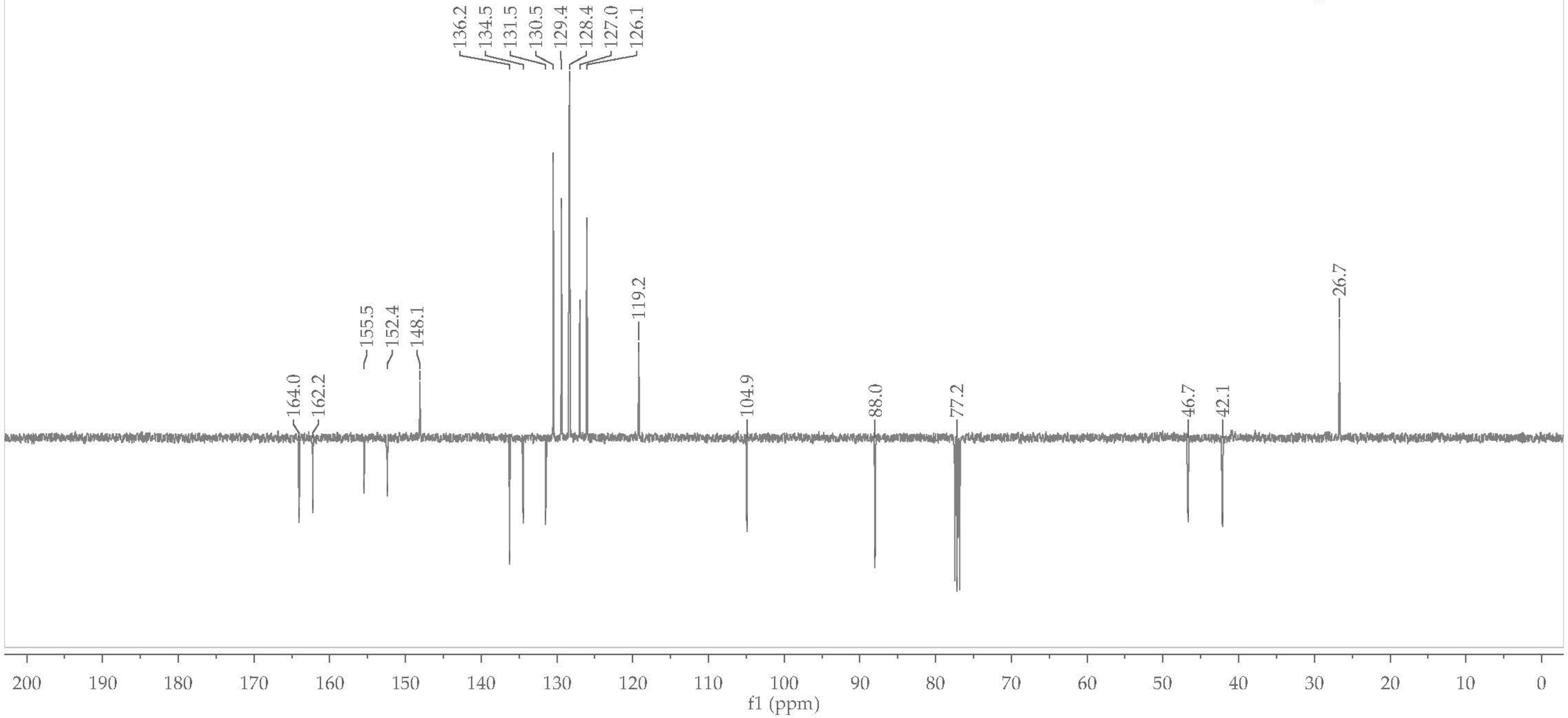

5-benzyl-*N*-(6-chloropyridazin-3-yl)-2-(4-(*N′*-hydroxycarbamimidoyl)phenyl)-5-methyl-4,5-dihydrofuran-3-carboxamide (23) - $C_{24}H_{22}ClN_5O_3$

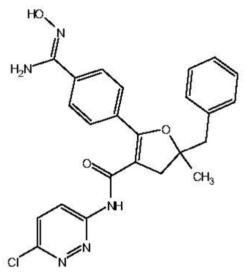

^{1}H NMR (400 MHz, $CDCl_3$): δ (ppm) 8.65 (s, 1H, NH), 8.47 (d, $^{3}J_{H\text{-}H}$ = 9.2 Hz, 1H, CH_{Ar}), 7.66 (s, 4H, 4CH_{Ar}), 7.42 (d, $^{3}J_{H\text{-}H}$ = 9.2 Hz, 1H, CH_{Ar}), 7.30-7.20 (m, 5H, 5CH_{Ar}), 5.08 (br s, 2H, NH_2), 3.19 (d, $^{2}J_{H\text{-}H}$ = 14.4 Hz, 1H, H-(CH_2)), 3.00 (s, 2H, CH_2), 2.90 (d, $^{2}J_{H\text{-}H}$ = 14.4 Hz, 1H, H-(CH_2)), 1.46 (s, 3H, CH_3). OH not observed.

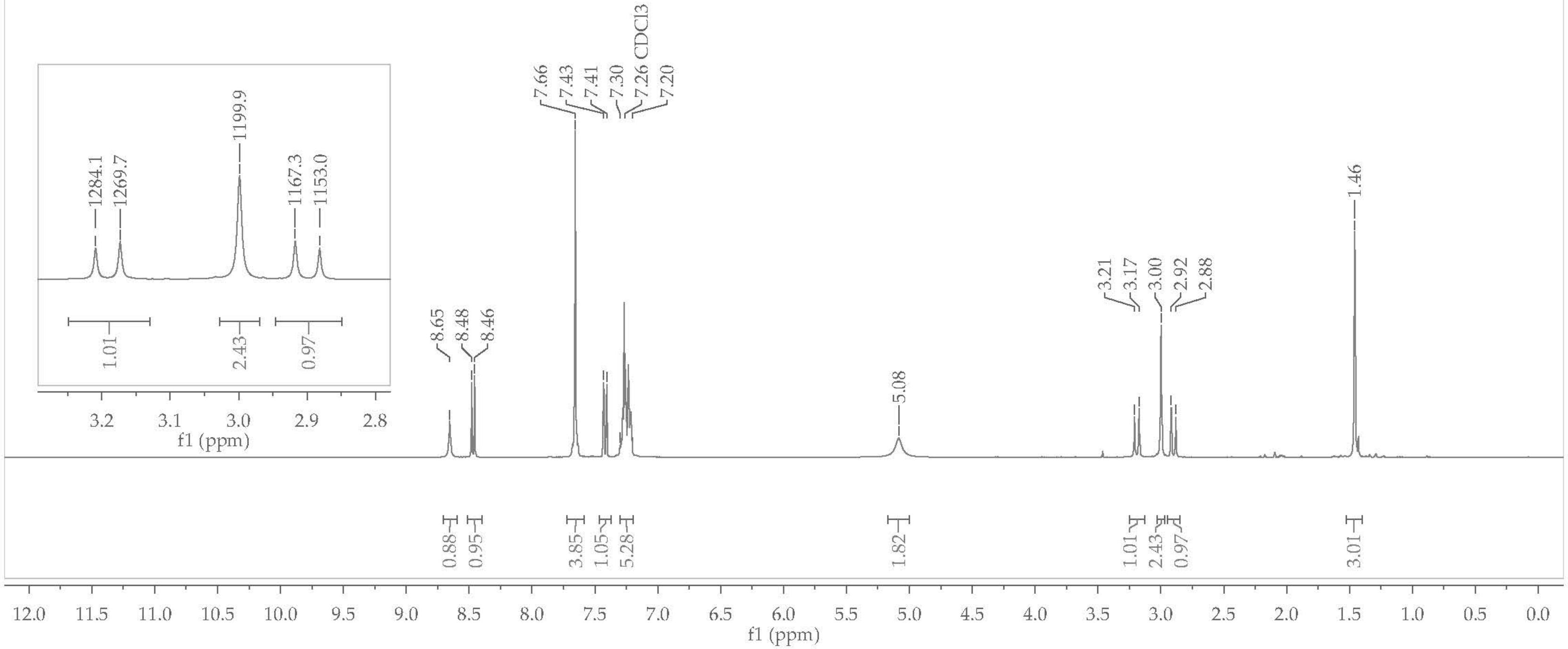

5-benzyl-*N*-(6-chloropyridazin-3-yl)-2-(4-(*N′*-hydroxycarbamimidoyl)phenyl)-5-methyl-4,5-dihydrofuran-3-carboxamide (23) - $C_{24}H_{22}ClN_5O_3$

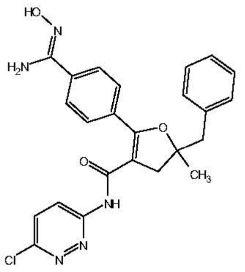

^{13}C NMR (100 MHz, $CDCl_3$): δ (ppm) 163.8 (C), 162.6 (C), 154.6 (C), 151.9 (C), 148.2 (C), 136.2 (C), 134.0 (C), 132.0 (C), 130.7 (2CH_{Ar}), 130.0 (CH_{Ar}), 129.6 (2CH_{Ar}), 128.5 (2CH_{Ar}), 127.2 (2CH_{Ar}), 126.4 (CH_{Ar}), 121.5 (CH_{Ar}), 105.0 (C), 88.4 (C), 46.8 (CH_2), 42.0 (CH_2), 26.9 (CH_3).

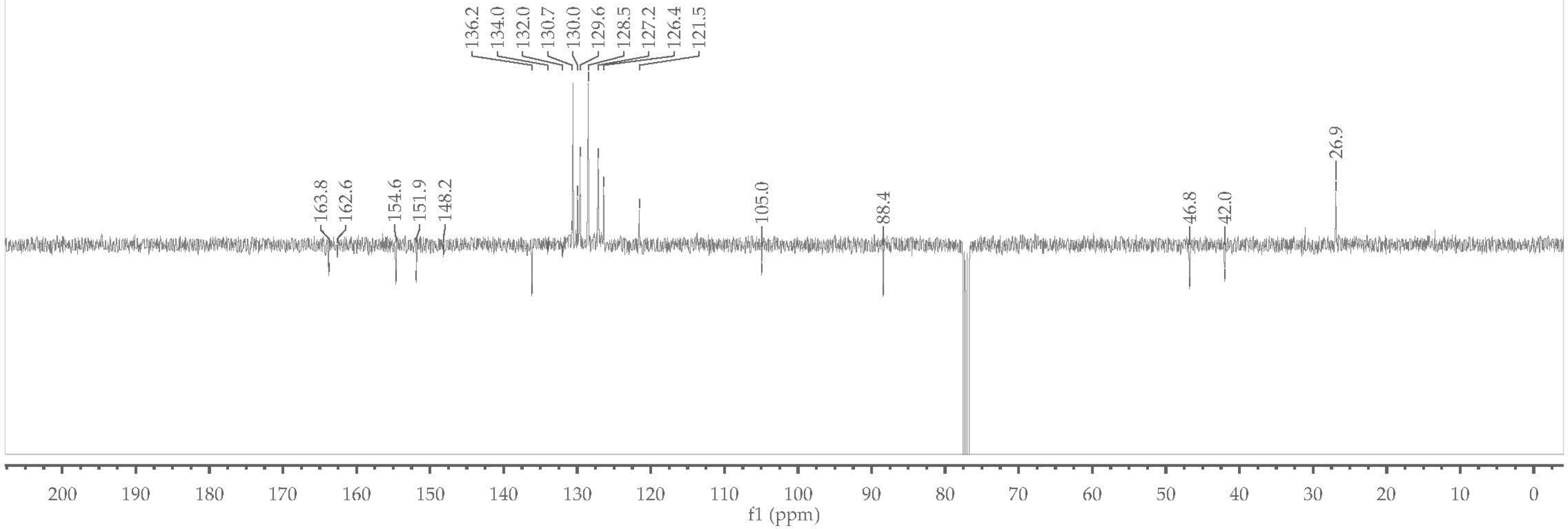

5-Benzyl-2-(4-(*N'*-hydroxycarbamimidoyl)phenyl)-5-methyl-*N*-(pyrazin-2-yl)-4,5-dihydrofuran-3-carboxamide (24) - $C_{24}H_{23}N_5O_3$

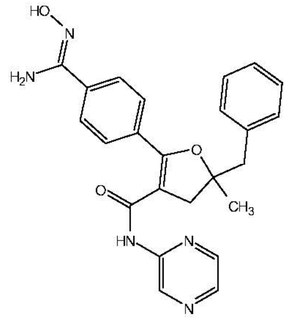

^{1}H NMR (400 MHz, $CDCl_3$): δ (ppm) 9.50 (s, 1H, NH), 8.27 (d, $^3J_{H\text{-}H}$ = 2.7 Hz, 1H, CH_{Ar}), 8.14 (d, $^3J_{H\text{-}H}$ = 2.7 Hz, 1H, CH_{Ar}), 7.78 (s, 1H, CH_{Ar}), 7.70 (s, 4H, $4CH_{Ar}$), 7.31-7.24 (m, 5H, $5CH_{Ar}$), 5.14 (br s, 2H, NH_2), 3.20 (d, $^2J_{H\text{-}H}$ = 14.4 Hz, 1H, H-(CH_2)), 3.02 (s, 2H, CH_2), 2.91 (d, $^2J_{H\text{-}H}$ = 14.4 Hz, 1H, H-(CH_2)), 1.48 (s, 3H, CH_3). OH not observed.

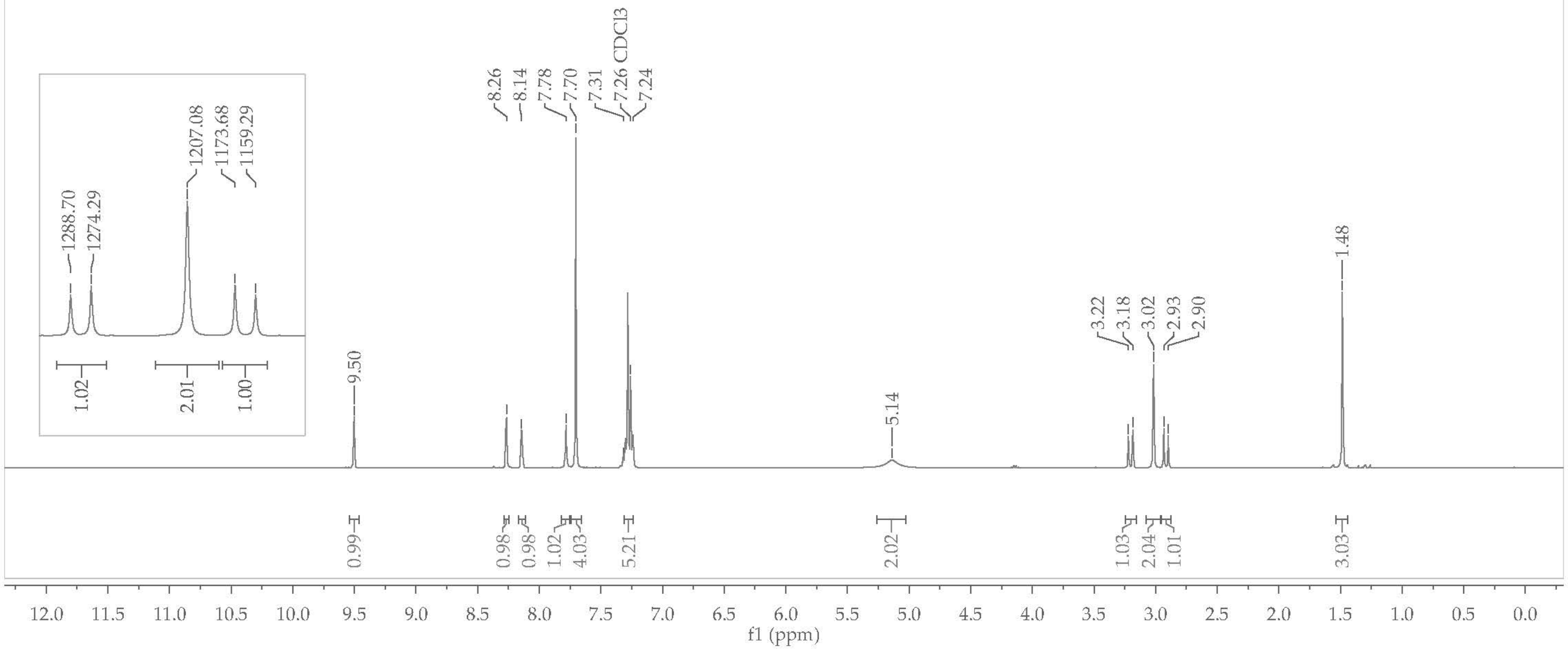

5-Benzyl-2-(4-(*N'*-hydroxycarbamimidoyl)phenyl)-5-methyl-*N*-(pyrazin-2-yl)-4,5-dihydrofuran-3-carboxamide (24) - $C_{24}H_{23}N_5O_3$

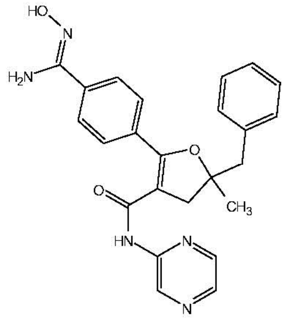

^{13}C NMR (100 MHz, $CDCl_3$): δ (ppm) 163.2 (C), 161.9 (C), 152.4 (C), 148.4 (C), 141.9 (CH_{Ar}), 139.7 (CH_{Ar}), 137.3 (CH_{Ar}), 136.2 (C), 134.2 (C), 131.7 (C), 130.5 ($2CH_{Ar}$), 129.6 ($2CH_{Ar}$), 128.4 ($2CH_{Ar}$), 127.1 (CH_{Ar}), 126.1 ($2CH_{Ar}$), 104.9 (C), 88.0 (C), 46.8 (CH_2), 42.3

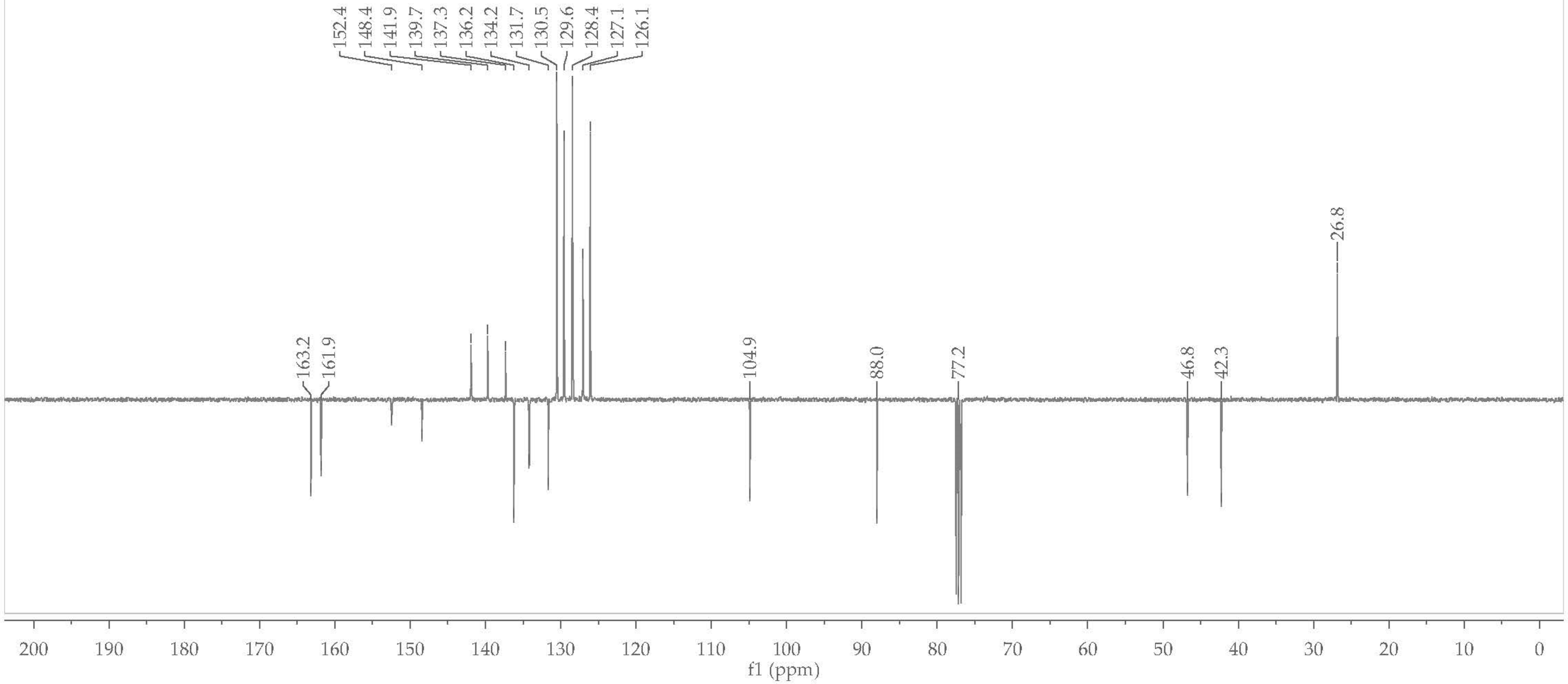

5-Benzyl-*N*-(1,5-dimethyl-1H-pyrazol-3-yl)-2-(4-(*N'*-hydroxycarbamimidoyl)phenyl)-5-methyl-4,5-dihydrofuran-3-carboxamide (25) - $C_{25}H_{27}N_5O_3$

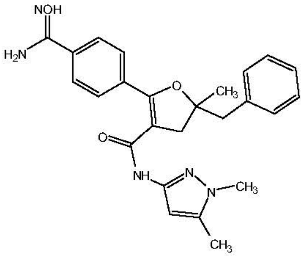

^{1}H NMR (400 MHz, $CDCl_3$): δ (ppm) 7.90 (s, 1H, NH), 7.71 (d, $^{3}J_{H\text{-}H}$ = 8.4 Hz, 2H, $2CH_{Ar}$), 7.64 (d, $^{3}J_{H\text{-}H}$ = 8.4 Hz, 2H, $2CH_{Ar}$), 7.30-7.18 (m, 5H, $5CH_{Ar}$), 6.47 (s, 1H, CH_{Ar}), 5.00 (br s, 2H, NH_2), 3.63 (s, 3H, CH_3), 3.06 (d, $^{2}J_{H\text{-}H}$ = 14.5 Hz, 1H, H-(CH_2)), 2.93 (d, $^{2}J_{H\text{-}H}$ = 13.7 Hz, 1H, H-(CH_2)), 2.86 (d, $^{2}J_{H\text{-}H}$ = 13.7 Hz, 1H, H-(CH_2)), 2.78 (d, $^{2}J_{H\text{-}H}$ = 14.5 Hz, 1H, H-(CH_2)), 2.21 (s, 3H, CH_3), 1.33 (s, 3H, CH_3). OH not observed.

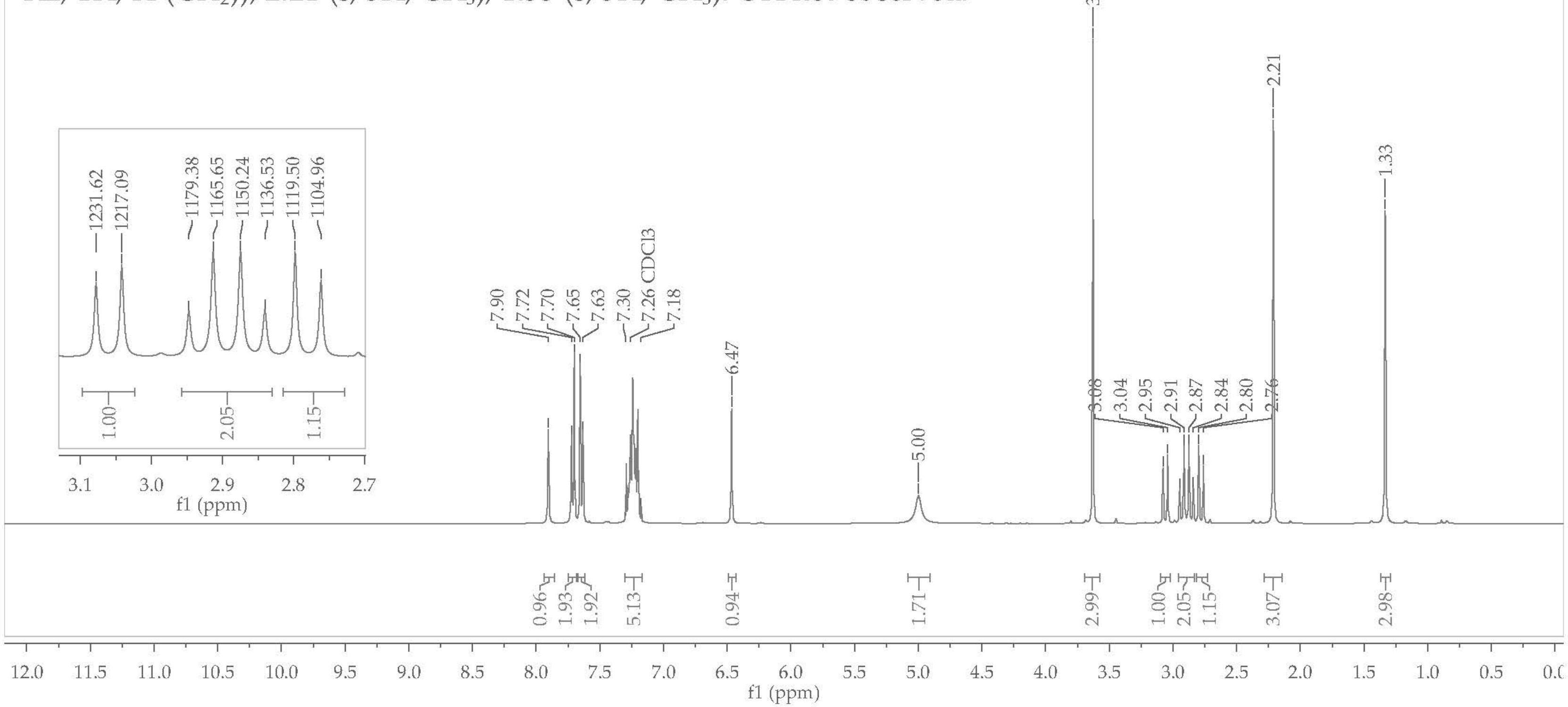

5-Benzyl-*N*-(1,5-dimethyl-1H-pyrazol-3-yl)-2-(4-(*N'*-hydroxycarbamimidoyl)phenyl)-5-methyl-4,5-dihydrofuran-3-carboxamide (25) - $C_{25}H_{27}N_5O_3$

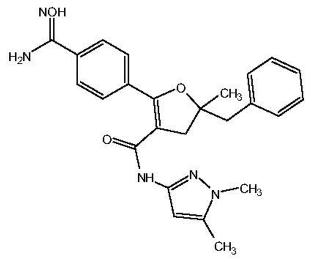

^{13}C NMR (100 MHz, $CDCl_3$): δ (ppm) 162.5 (C), 159.7 (C), 152.2 (C), 145.8 (C), 139.5 (C), 136.5 (C), 134.2 (C), 131.7 (C), 130.5 ($2CH_{Ar}$), 129.3 ($2CH_{Ar}$), 128.2 ($2CH_{Ar}$), 126.8 (CH_{Ar}), 125.7 ($2CH_{Ar}$), 105.0 (C), 97.5 (CH_{Ar}), 87.0 (C), 46.6 (CH_2), 42.7 (CH_2), 35.5 (CH_3), 26.5 (CH_3), 11.4 (CH_3).

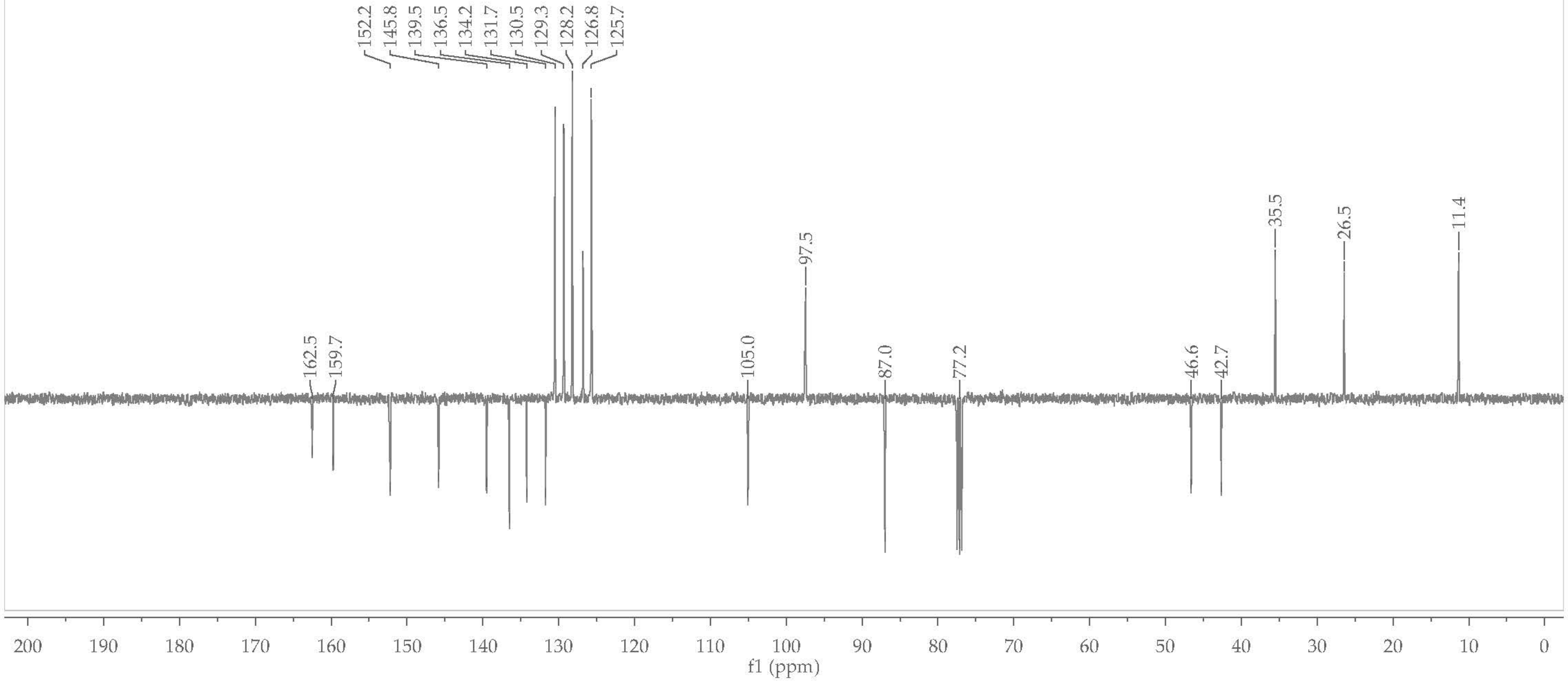

Supplement: Supplementary file 1 [file molecules-29-05469-s001.zip › molecules-3296746-supplementary.pdf]
